# Electron-deficient pyridinium salts/thiourea cooperative catalyzed *O*-glycosylation via activation of *O*-glycosyl trichloroacetimidate donors

**DOI:** 10.3762/bjoc.13.236

**Published:** 2017-11-09

**Authors:** Mukta Shaw, Yogesh Kumar, Rima Thakur, Amit Kumar

**Affiliations:** 1 Department of Chemistry, Indian Institute of Technology Patna, Bihta 801106, Bihar, India; 2 Department of Chemistry, National Institute of Technology Patna, Patna 800005, Bihar, India

**Keywords:** cooperative catalysis, electron-deficient pyridinium salts, *O*-glycoside, regioselectivity, thiourea

## Abstract

The glycosylation of *O*-glycosyl trichloroacetimidate donors using a synergistic catalytic system of electron-deficient pyridinium salts/aryl thiourea derivatives at room temperature is demonstrated. The acidity of the adduct formed by the 1,2-addition of alcohol to the electron-deficient pyridinium salt is increased in the presence of an aryl thiourea derivative as an hydrogen-bonding cocatalyst. This transformation occurs under mild reaction conditions with a wide range of *O*-glycosyl trichloroacetimidate donors and glycosyl acceptors to afford the corresponding *O*-glycosides in moderate to good yields with predictable selectivity. In addition, the optimized method is also utilized for the regioselective *O*-glycosylation by using a partially protected acceptor.

## Introduction

The glycosidic linkage is the principal bond present in a crucial class of biomolecules such as oligosaccharides and glycoconjugates, where one sugar unit is linked with another sugar unit or any other molecules (aglycons) [1–4]. Owing to their high importance, several efficient protocols have been developed for the stereoselective glycosylation in the past few decades [5–10]. However, the synthesis of fundamental glycosidic bonds with high efficiency and selectivity yet remains one of the major challenges for organic chemists, in particular, carbohydrate chemists.

**Scheme 1:**
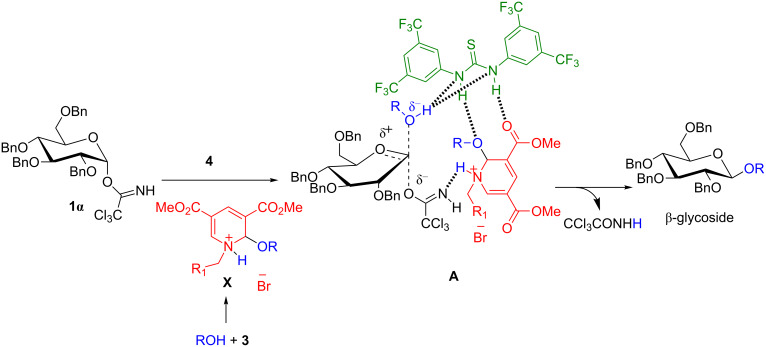
Mechanistic hypothesis for work.

Nature extensively employs small organic molecules as catalysts for the acceleration of many important biochemical reactions, such as glycosyltransferase reactions, hydrolysis of strong amide bonds and others [11–13]. Taking inspiration from nature, in the last few decades chemists around the world have utilized organic molecules to accelerate many imperative organic transformations [14–17]. One of the major applications of organocatalysis lies in the field of enantioselective synthesis, where organocatalysts are considered as fundamental tools in the catalysis toolbox [18–22]. Moreover, the reactivity and selectivity of organocatalysts can be further amplified in the presence of other cocatalysts known as “cooperative catalysis” [23]. In particular, cooperativity between Brønsted acids and hydrogen-bonding cocatalysts such as thiourea derivatives has attracted much interest [24–29]. Despite the broad application of cooperative catalysis, it is still uncommonly employed in the area of carbohydrate chemistry, especially for glycosylation reactions, due to the prerequisite of having both catalysts being compatible under the reaction conditions. The Schmidt group has successfully applied the synergistic catalysts (thiourea derivatives with phosphorus acids) for stereoselective *O*-glycoside bond formation [30]. Similarly, Galan et al. reported a method for the preparation of 2-deoxyglycosides from glycals under the influence of cooperative catalysis (chiral phosphoric acids/thiourea derivatives) [31]. Encouraged by these reports and our own research interest in developing stereoselective glycosylation methods, we decided to focus our attention on the synthesis of glycosides via cooperative catalysis. A highly reactive glycosyl donor for instance, *O*-glycosyl trichloroacetimidate, generally requires a p*K*_a_ value less than 5 for activation at room temperature [32–36]. It is known from the literature that pyridinium salts exhibit p*K*_a_ values of about 5.2 [37]. Of late, Berkessel et al. disclosed an elegant method, where different electron deficient pyridinium salts (expected p*K*_a_ values less than 5) were used as a catalyst for the activation of glycals to provide stereoselective 2-deoxyglycosides with high yields [38]. Based on this fact, we anticipated that for electron-deficient pyridinium salts the p*K*_a_ value would be further diminished in the presence of hydrogen-bonding cocatalysts such as thiourea derivatives. The presence of Schreiner′s thiourea in the reaction medium enhances the acidity of the ammonium salt due to doubling their dual hydrogen bonding ability with the carboxylate and the alkoxy group of the ammonium salt. A thiourea derivative also enhances the nucleophilicity of the glycosyl acceptor by imparting a partial negative charge on it. Hence, the application of the synergistic catalyst system consisting of electron-deficient pyridinium salts/thiourea derivatives for glycosidic bond formation will be an exciting addition to the literature (Scheme 1).

## Results and Discussion

**Figure 1:**
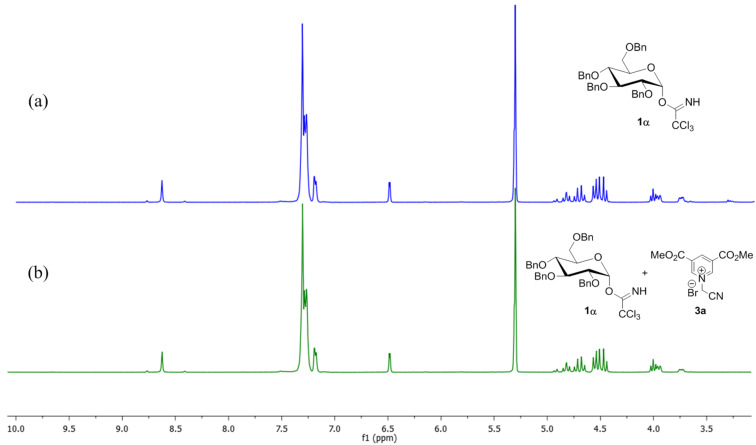
^1^H NMR (a) glycosyl donor **1α** and (b) a mixture of **1α** and 10 mol % **3a** in CD_2_Cl_2_ at room temperature.

**Figure 2:**
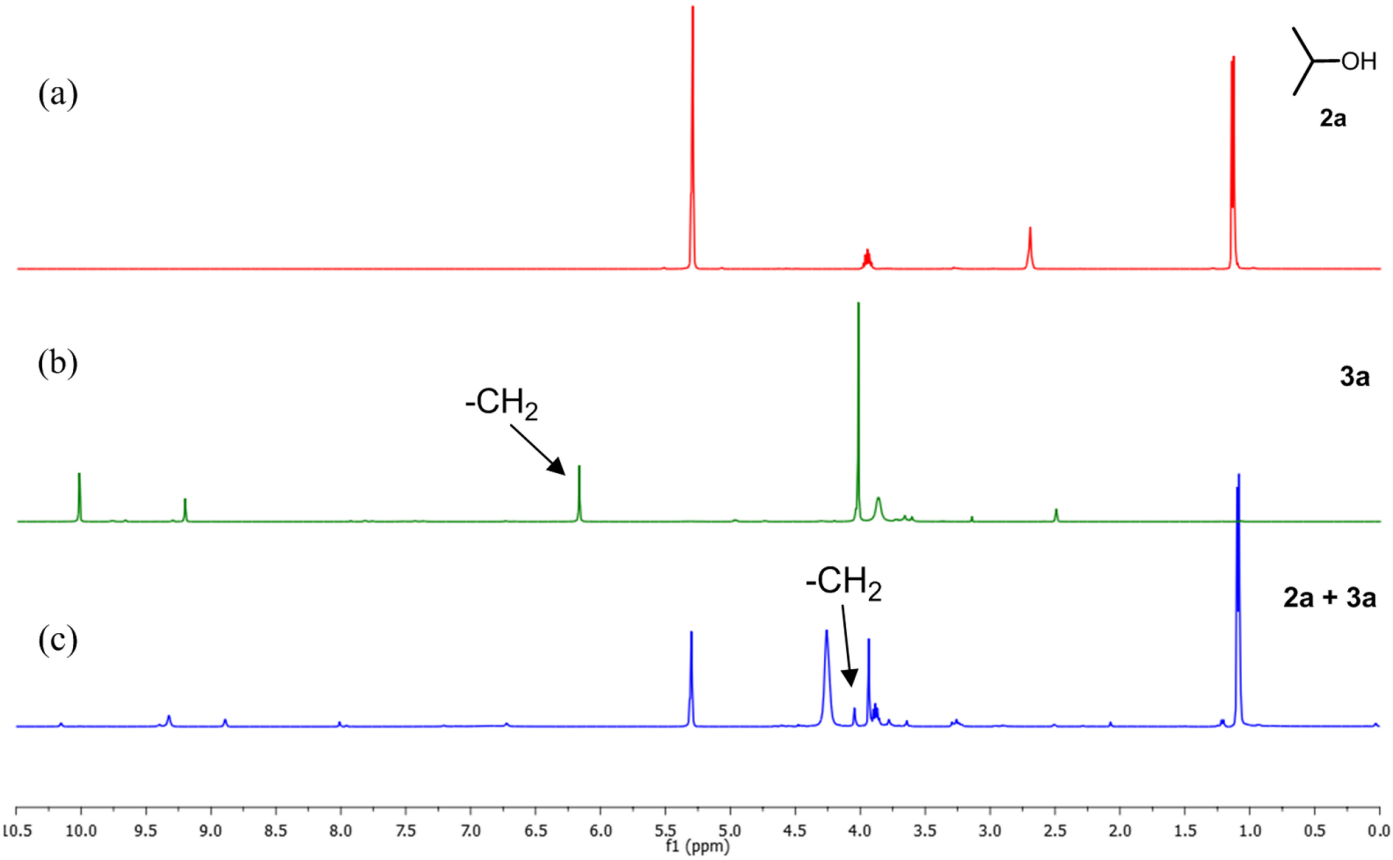
^1^H NMR (a) glycosyl acceptor **2a**, (b) pyridinium salt **3a** (in DMSO-*d*_6_) and (c) a mixture of **2a** and **3a** in 1:1 ratio in CD_2_Cl_2_ at room temperature.

To check our hypothesis, a series of ^1^H NMR spectroscopic studies were conducted by selecting commonly used *O*-glucopyranosyl trichloroacetimidate **1α** [39–41] as glycosyl donor and 3,5-di(methoxycarbonyl)-*N*-(cyanomethyl)pyridinium bromide (**3a**) as a catalyst. For example, when glycosyl donor **1α** was treated with catalyst **3a** (10 mol %) at room temperature for 4 h (Table 1, entry 1) in CD_2_Cl_2_ solvent, it was observed that there is neither any interaction of **1α** with catalyst **3a** nor decomposition of **1α** (Figure 1b) as the peak position of **1α** remained unchanged. The salt remains insoluble in CD_2_Cl_2_ and hence did not show any peak in the ^1^H NMR spectra. However, when the mixture of electron-deficient pyridinium salt **3a** and glycosyl acceptor **2a** (1:1) was dissolved in CD_2_Cl_2_ and investigated by ^1^H NMR, an upfield shift of the -CH_2_- peak of **3a** from δ 6.17 to δ 4.03 (Figure 2c) was observed. This result clearly supports the possible formation of 1,2-adduct **X**, on the reaction of **3a** with **2a** which is eventually responsible for the loss of aromaticity of the pyridinium ring.

Based on the outcomes of ^1^H NMR spectroscopic studies, we started optimizing the reaction conditions. Upon treatment of glycosyl donor **1α** and glycosyl acceptor **2a** in 1:1.1 molar ratio with 10 mol % of **3a** in dry DCM at room temperature, the desired *O*-glycoside **5a** was isolated in 56% yield and with poor selectivity (Table 1, entry 2). The use of 25 mol % of **3a** was required to drive the reaction to completion with 86% yield (Table 1, entry 3). This result, as envisaged, was indeed interesting and encouraging, which clearly indicates the ability of the electron-deficient pyridinium salt to activate the trichloroacetimidate donor. However, it took longer reaction time and required higher catalyst loading (up to 25 mol %). This outcome can be attributed to lower acidity of the ammonium salt formed by 1,2-addition of the acceptor to the pyridinium salt. The conjugate base formed after the release of a proton from the ammonium salt may be quite stable to impart a negative charge to the acceptor oxygen.

**Table 1:** Optimization of reaction conditions^a^.

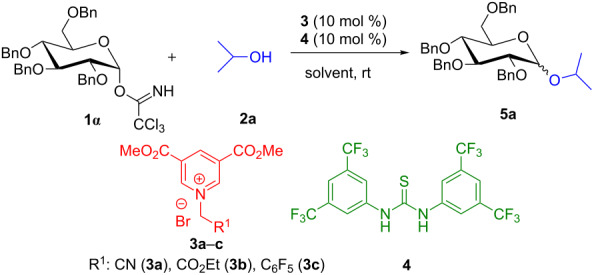					

entry	catalyst	cocatalyst **4**	solvent	reaction time	yield^b^ (α/β ratio^c^)

1^d^	**3a**	–	DCM	4 h	n.r., n.d.^e^
2	**3a**	–	DCM	24 h	56% (1:1)^f^
3^g^	**3a**	–	DCM	4 h	86% (1.1:1)
**4**	**3a**	**+**	**DCM**	**2 h**	**90% (2.2:1)**
5	**3b**	+	DCM	6 h	72% (2:1)
6	**3c**	+	DCM	8 h	64% (1.7:1)
7^h^	**3a**	+	DCM	5 h	86% (2.1:1)
8	**3a**	+	ACN	4 h	56 % (2.1:1)
9	**3a**	+	THF	7 h	37% (1:1)
10	**3a**	+	toluene	24 h	trace
11	**3a**	+	DCE	3 h	80% (1.4:1)
12^i^	**3a**	+	DCM	5 h	82%(2:1)
13	HBr	–	DCM	8 h	trace^j^
14^k^	**3a**	–	DCM	4 h	n.r.

^a^Reaction conditions: **1α** (0.15 mmol), **2a** (0.165 mmol), **3a–c** (10 mol %), **4** (10 mol %), solvent (3 mL), at room temperature under nitrogen atmosphere. ^b^Yield of isolated product. ^c^Anomeric ratios were determined by ^1^H NMR spectroscopy. ^d^**1α** was stirred with 10 mol % **3a** for 4 h at room temperature. ^e^n.r. – no reaction, n.d. – no decomposition. ^f^Reaction was not completed. ^g^25 mol % of **3a** was used. ^h^Performed at 0 °C. ^i^Inverse addition condition. ^j^A trace amount of glucosyl bromide was also formed. ^k^25 mol % of 2,4,6-trimethylpyridine was added.

**Figure 3:**
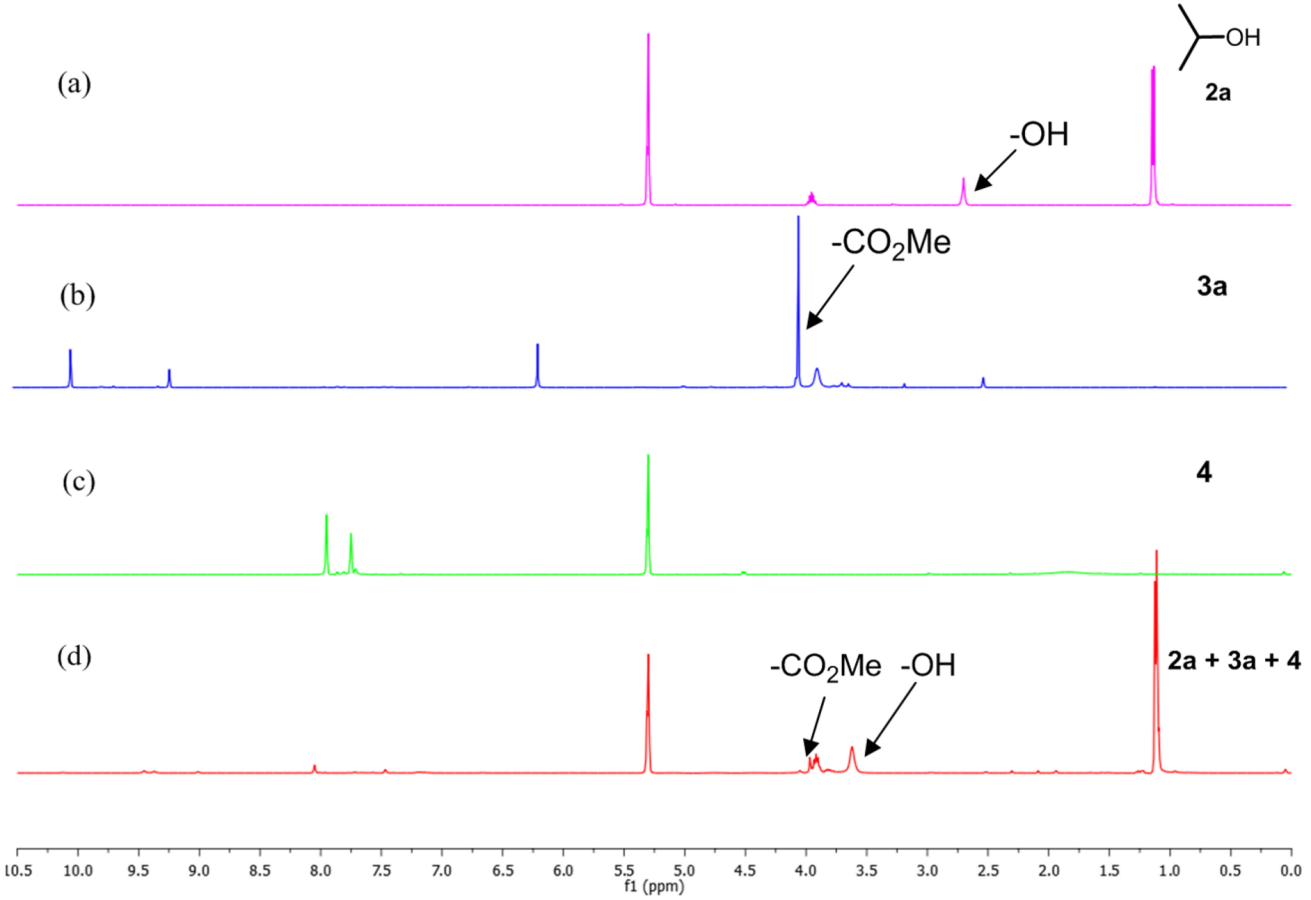
^1^H NMR (a) glycosyl acceptor **2a**, (b) pyridinium salt **3a** (in DMSO-d_6_), (c) aryl thiourea and (d) a mixture of **2a**, **3a** (10 mol %) and **4** (10 mol %) in CD_2_Cl_2_ at room temperature.

The acidity of the ammonium salt may be enhanced by the introduction of a cocatalyst such as an aryl thiourea derivative, which has the ability to form a dual hydrogen bond with the carboxylate and the alkoxy group of the ammonium salt [42–44]. To ensure our postulation, a ^1^H NMR spectroscopic study was carried out with a mixture of glycosyl acceptor **2a**, pyridinium salt **3a** (10 mol %) and aryl thiourea **4** (10 mol %) in CD_2_Cl_2_ at room temperature (Figure 3). The -Me peak of -CO_2_Me of the ammonium salt shifted from δ 4.09 to δ 3.97, which indirectly confirms the presence of hydrogen bonding between -NH of the thiourea and the carbonyl carbon of -CO_2_Me of **3a** (Figure 3d). Also, the magnification of the nucleophilicity of the glycosyl acceptor by the thiourea derivative results in shifting of the -OH peak from δ 2.70 to δ 3.62 (Figure 3d).

Once this understanding was gained from ^1^H NMR studies, aryl thiourea cocatalyst **4** (10 mol %) was added with 10 mol % of **3a** to the reaction mixture of **1α** and **2a** and pleasingly the reaction was completed within a short span of time, glycoside **5a** was obtained with improved yield (90%) and selectivity (α:β 2.2:1 ratio, Table 1, entry 4). Furthermore, several other catalysts were also screened for glycosylation, such as 3,5-di(methoxycarbonyl)-*N*-[(ethoxycarbonyl)methyl]pyridinium bromide (**3b**, Table 1, entry 5), 3,5-di(methoxycarbonyl)-*N*-[(pentafluorophenyl)methyl]pyridinium bromide (**3c**, Table 1, entry 6). However, the results were not up to our expectation and the desired glycoside was obtained in low yield. Once, we fixed the catalyst for glycosylation, further parameters were also optimized. Lowering the reaction temperature (room temperature to 0 °C), the product formation rate slowed down and the selectivity remains unchanged (Table 1, entry 7). Similarly, changing the solvent system to acetonitrile, tetrahydrofuran, toluene, and dichloroethane had an adverse effect on the reaction rate, yield and selectivity (Table 1, entries 8–11). Performing the reaction under inverse addition conditions had no impact on the selectivity of glycoside formation (Table 1, entry 12) [39–40]. Aware of the fact that HBr, which is eventually generated in situ during the course of reaction from ammonium salt **X**, might be the potential catalyst for the activation of the glycosyl trichloroacetimidate donor, we conducted an additional experiment. Glycosyl donor **1α** was treated with 25 mol % of HBr instead of pyridinium salt **3a** (Table 1, entry 13) where only a trace of glycoside **5a** and some glycosyl bromide were formed along with the hydrolyzed product. Hence, it could be concluded that HBr is not the real catalyst in this cooperative catalysis. Further, the addition of acid scavenger such as 2,4,6-trimethylpyridine inhibits the formation of the glycoside, which indirectly supports that the pyridinium salt is a decisive catalyst for the activation of the glycosyl trichloroacetimidate donor (Table 1, entry 14). Therefore, the optimal reaction conditions for *O*-glycosylation are the following: the use of 3,5-di(methoxycarbonyl)-*N*-(cyanomethyl)pyridinium bromide (**3a**) as catalyst (10 mol %), thiourea derivative **4** as cocatalyst (10 mol %) and dichloromethane as solvent at room temperature (Table 1, entry 4).

With the optimized conditions in hand, our emphasis was then focused on the exploration of the synergistic catalytic system on glycosylation of **1α** with several acceptors (Table 2). In all cases, reactions proceeded smoothly within 2–6 h and in good yields with moderate to good selectivity, as determined by the ^1^H and ^13^C NMR spectra. Glycosylation with less sterically hindered primary alcohols, e.g., allyl alcohol (**2b**), benzyl alcohol (**2c**), 4-methoxy benzyl alcohol (**2d**), and secondary alcohols, e.g., cyclohexanol (**2e**), cyclopentanol (**2f**) produced their corresponding glucosides **5b–f** in 78–88% yields and with moderate α-selectivity (Table 2, entries 1–5). It is important to note that, when a halogenated primary alcohol such as 2-bromoethanol (**2g**) was treated with glycosyl donor **1α**, it gave exclusively β*-*glycoside **5g** in 72% yield (Table 2, entry 6). However, 3-chloropropanol (**2h**) as acceptor procured glucoside **5h** in 70% yield with marginal selectivity (Table 2, entry 7). Similarly, the reaction with the bulky primary alcohol, 1-adamantanemethanol (**2i**) produced the desired glucoside **5i** in 75% yield and moderate selectivity (Table 2, entry 8). Further, reactions with hindered acceptors, for instance, 1-adamantanol (**2j**), (+)-menthol (**2k**), (–)-menthol (**2l**), cholesterol (**2m**) required longer reaction times with high catalyst loading and produced the desired glucosides **5j–m,** respectively, in moderate to good yields (43–67%) and with good α-selectivity (Table 2, entries 9–12). As expected, on treatment with a sugar-based acceptor such as 1,2:3,4-di-*O*-isopropylidene-D-galactose (**2n**), the corresponding glycoside **5n** was produced in 72% yield with moderate selectivity (Table 2, entry 13) and the acid sensitive group survived well [34].

**Table 2:** Acceptor scope in glycosylation reaction with donor **1α**^a^.

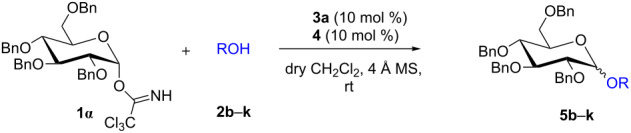					

entry	ROH	product	time (h)	yield^b^	α/β ratio^c^

1	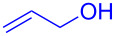 **2b**	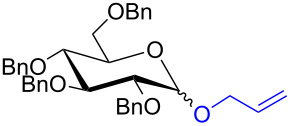 **5b**	2	88%	1.2:1
2	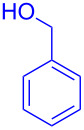 **2c**	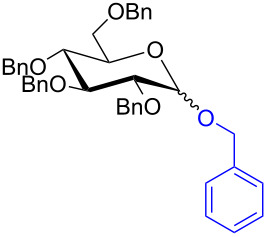 **5c**	2	88%	2:1
3	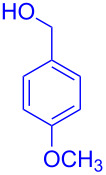 **2d**	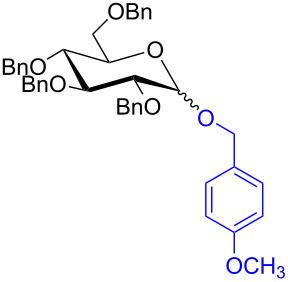 **5d**	2	82%	3.3:1
4	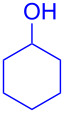 **2e**	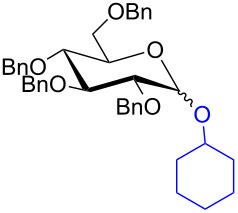 **5e**	2	78%	2.1:1
5	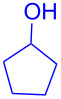 **2f**	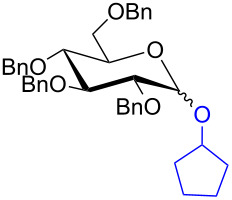 **5f**	2	85%	2.5:1
6	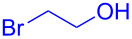 **2g**	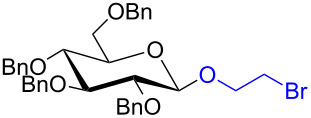 **5g**	2	72%	β only
7	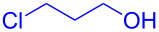 **2h**	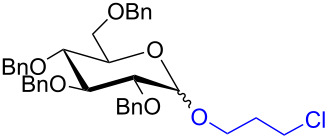 **5h**	2	70%	1.1:1
8	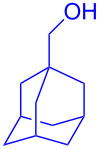 **2i**	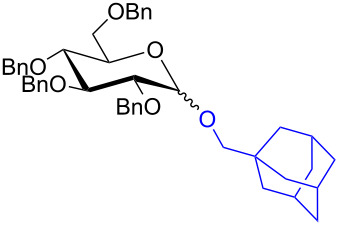 **5i**	6	75%	3.3:1
9^d^	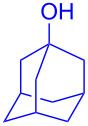 **2j**	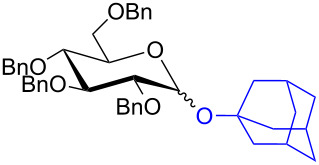 **5j**	5	43%	2:1
10^d^	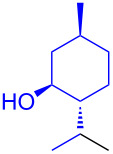 **2k**	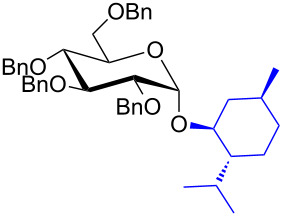 **5k**	4	61%	α only
11^d^	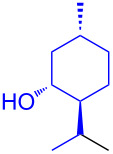 **2l**	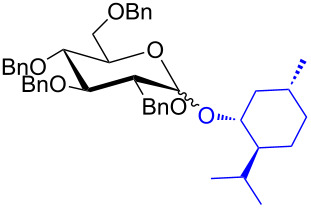 **5l**	4	67%	5:1
12^d^	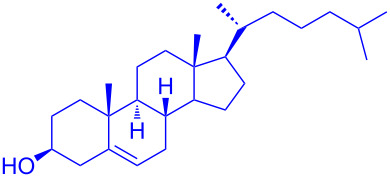 **2m**	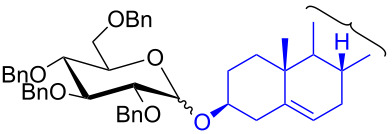 **5m**	6	60%	10.1:1
13	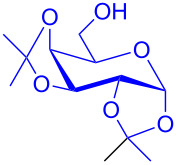 **2n**	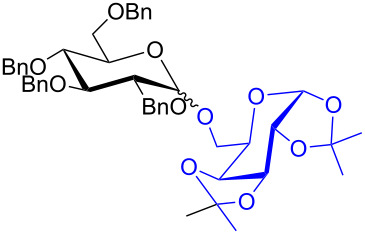 **5n**	3	72%	1:1.3

^a^Reaction conditions: **1α** (0.15 mmol), **2a**–**n** (0.165 mmol), **3a** (10 mol %), **4** (10 mol %), solvent (3 mL), at room temperature under nitrogen atmosphere. ^b^Yield of isolated product. ^c^Anomeric ratios were determined by ^1^H NMR spectroscopy. ^d^20 mol % of **3a** and 20 mol % of **4** was used.

To further demonstrate the efficacy of this method other important glycosides were synthesized with different donors as tabulated in Table 3. Glycosylation of D-galactopyranosyl trichloroacetimidate **6α** with a variety of glycosyl acceptors, e.g., **2c**, **2g**, **2h**, **2i** and **2n** under the optimized reaction conditions gave their corresponding galactosides **9**–**13**, respectively, with moderate selectivity (Table 3, entry 1–5) [40–41]. Similarly, the reaction of D-mannopyranosyl trichloroacetimidate **7α** with glycosyl acceptors **2c**, **2g**, **2h,** and **2n** produces their corresponding mannosides **14**–**18** (61–78% yields) with moderate selectivity (Table 3, entries 6–10) [40–41]. Gratifyingly, the highest stereoselective outcome was observed when 4,6-*O*-benzylidine-2,3-di-*O*-benzyl-α-D-glucopyranosyl trichloroacetimidate **8α** was used as a glycosyl donor. For example, when **8α** was treated with different donors such as **2i**, **2m**, **2n** under the optimized conditions, glucosides **19**–**21** were procured in good yields with excellent α-selectivity (Table 3, entry 11–13) [45].

**Table 3:** Glycosylation of donors **6α**–**8α** with variety of acceptors^a^.

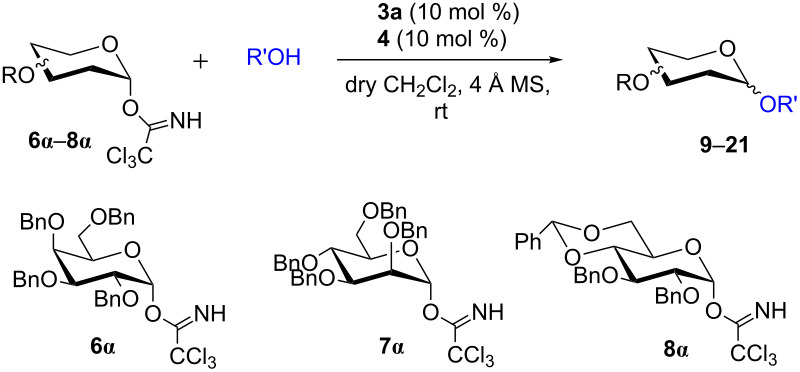						

entry	donor	acceptor	product	time (h)	yield^b^	α/β ratio^c^

1	**6α**	2c	9	2	91%	1.7:1
2		2g	10	2	74%	2.1:1
3		2h	11	2	81%	1:4.6
4		2i	12	6	73%	2.6:1
5		2n	13	3	62%	1.4:1
6	**7α**	2c	14	2	74%	1.2:1
7		2g	15	2	78%	3:1
8		2h	16	2	77%	1.9:1
9^d^		2m	17	7	68%	1:1.9
10		2n	18	3	61%	α only
11	**8α**	2i	19	6	61%	α only
12^d^		2m	20	8	54%	α only
13		2n	21	4	61%	α only

^a^Reaction conditions: donor (0.15 mmol), acceptor (0.165 mmol), **3a** (10 mol %), **4** (10 mol %), solvent (3 mL), at room temperature under nitrogen atmosphere. ^b^Yield of isolated product. ^c^Anomeric ratios were determined by ^1^H NMR spectroscopy. ^d^20 mol % of **3a** and 20 mol% of **4** was used.

**Scheme 2:**
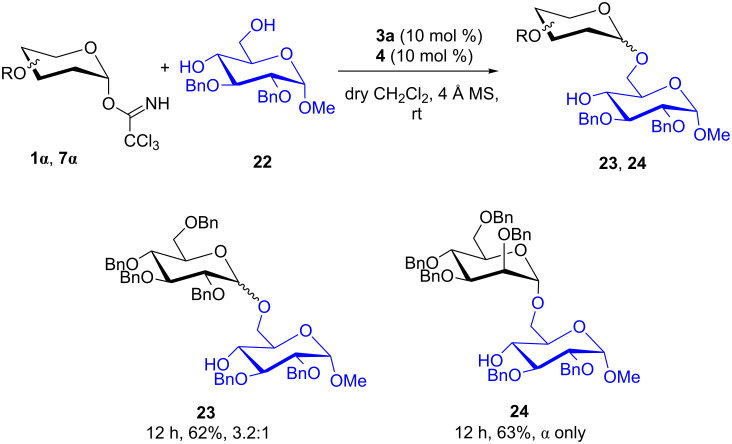
Synergistic electron-deficient pyridinium salt/aryl thiourea-catalyzed regioselective *O*-glycosylation.

The regioselective glycosylation is an important aspect in carbohydrate chemistry. It is pleasing to note that on reaction with partially protected acceptor **22** with glycosyl donors **1α** and **7α** under the optimized conditions lead to the regioisomeric products **23** and **24** [46–47] in moderate yields with good selectivity (Scheme 2).

### Plausible mechanism

**Figure 4:**
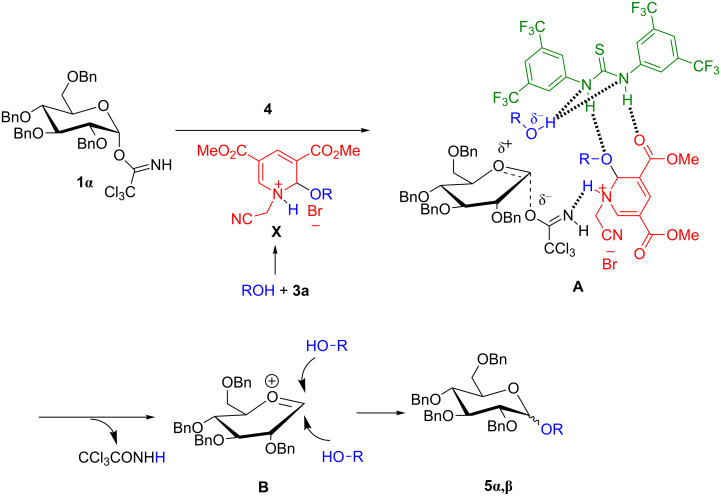
Plausible reaction mechanism.

To confirm the reaction pathway, few additional control experiments were carried out (for details see Supporting Information File 1). When the reaction was performed with 1.0 equiv of **3a** and acceptor **2a** under standard inverse procedure conditions, the desired *O*-glycoside **5a** was procured in 36% yield and 56% of **1α** was recovered through column chromatography. Therefore, we conclude that the reaction would have followed an intermolecular glycosylation reaction through an oxocarbenium ion. Combining all of these observations and results from earlier literature reports, a plausible reaction mechanism for the electron-deficient pyridinium salt/thiourea cocatalyzed glycosidation is outlined in Figure 4. It is presumed that at first electron-deficient pyridinium salt **3a** undergoes 1,2-addition with the acceptor to produce ammonium salt **X**. The addition of the thiourea derivative as hydrogen-bonding cocatalyst could activate the glycosyl donor by increasing the acidity of ammonium salt **X** to form an oxocarbenium intermediate **B**. Further, the nucleophilic attack of the acceptor to the oxocarbenium ion **B** would produce the desired glycoside **5**. Higher α-selectivity may be attributed to the anomeric effect.

## Conclusion

In conclusion, we have disclosed an efficient and general protocol for the glycosylation of trichloroacetimidate glycosyl donors using the concept of cooperativity between an electron-deficient pyridinium salt and an aryl thiourea derivative. ^1^H NMR studies divulge that a 1,2-adduct formation between the electron-deficient pyridinium salt and the glycosyl acceptor plays a crucial role for the activation of the trichloroacetimidate donors. The presence of thiourea derivatives further enhances the reaction rate and selectivity due to its dual hydrogen bonding ability. The reaction proceeds smoothly at room temperature with good to excellent yields and α-selectivity and is applicable to a wide range of glycosyl donors as well as acceptors. The advantage of this methodology lies in the usage of an environmentally benign catalyst, mild reaction conditions and the regioselective formation of *O*-glycosides.

## Supporting Information

File 1 Experimental procedures and analytical data.
