# Supplementary material for: Electron-deficient pyridinium salts/thiourea cooperative catalyzed O-glycosylation via activation of O-glycosyl trichloroacetimidate donors
Source: Beilstein J Org Chem. 2017 Nov 9;13:2385–95. doi: 10.3762/bjoc.13.236 (PMC5687052; doi:10.3762/bjoc.13.236)

Supporting Information

for

Electron-deficient pyridinium salts/thiourea cooperative catalyzed *O*-glycosylation via activation of *O*-glycosyl trichloroacetimidate donors

Mukta Shaw[1], Yogesh Kumar[1], Rima Thakur[2] and Amit Kumar*[1]

Address: [1]Department of Chemistry, Indian Institute of Technology Patna, Bihta 801106, Bihar, India and [2]Department of Chemistry, National Institute of Technology Patna, Patna 800005, Bihar, India

Email: Amit Kumar* - amitkt@iitp.ac.in

*Corresponding author

Experimental procedures and analytical data

Table of contents

Experimental section S1–S21

Controlled experiments S20

References……………...S21

NMR spectra………………………………………………………………………..……..S22

^{1}H, ^{13}C NMR of pyridinium salts **3a**–**3c** S24–S27

^{1}H, ^{13}C NMR of thiourea co-catalyst **4** S28

^{1}H, ^{13}C, COSY and HSQC NMR of compounds **5a**–**5n** S29–S55

^{1}H, ^{13}C, COSY and HSQC NMR of compounds **9**–**21** S56–S79

^{1}H, ^{13}C, COSY and HSQC NMR of compounds **23**,**24** S80–S83

Experimental section

1. General procedure

All chemicals were purchased as reagent grade and used without further purification, unless otherwise mentioned. Solvents were purified by standard procedures. All reactions were carried out under nitrogen atmosphere with freshly distilled solvents, unless otherwise mentioned. Glass wares, used for water-free reactions were dried for 12 h at 120 °C before use and allowed to cool either in desiccators or under reduced pressure. Molecular sieves (4 Å) were flame dried before use. Reactions were monitored by analytical thin-layer chromatography on silica gel 60 F_{254} precoated on aluminum plates (Merck). TLC plates were visualized by spraying 10% H_2SO_4 in EtOH and heating until spots appeared or under UV light (254 nm). Column chromatography was performed using silica gel (100–200 mesh). NMR spectra were recorded on an Avance III HD Bruker-400 spectrometer at 25 °C (^{1}H NMR: 400 MHz, ^{13}C NMR: 100 MHz). Chemical shifts (δ) are reported in parts per million (ppm) relative to tetra-methylsilane or solvent residual signals (^{1}H NMR: solvent $CDCl_3$, δ = 7.26 ppm; DMSO-d_6, δ = 2.50 ppm; CD_2Cl_2, δ = 5.30 ppm; ^{13}C NMR: solvent $CDCl_3$, δ = 77.22 ppm; DMSO-d_6, δ = 39.58 ppm). Mass spectra were recorded on an Agilent 6520 Q-Tof (positive mode ESIMS) mass spectrometer. Melting points were recorded on a Stuart digital melting point apparatus.

2. General procedure for glycosylation

(a) Normal procedure: To a stirred suspension of trichloroacetimidate donor (0.15 mmol, 1.0 equiv), acceptor (0.165 mmol, 1.1 equiv) and 4 Å MS (200 mg) in dry DCM (3 ml) was added aryl thiourea **4** (0.015 mmol, 0.1 equiv) at room temperature. The mixture was stirred for 10 min at room temperature under a nitrogen atmosphere. Then, electron-deficient pyridinium salt

(0.015 mmol, 0.1 equiv) was added and the resulting reaction mixture was stirred at room temperature until total consumption of glycosyl donor monitored by TLC. After completion of reaction, the mixture was concentrated in vacuo and purified by column chromatography using different fractions of acetone in hexane as eluting solvent to afford the desired glycosides.

(b) Inverse Procedure: To a stirred suspension of glycosyl acceptor (0.165 mmol, 1.1 equiv), aryl thiourea **4** (0.015 mmol, 0.1 equiv) and 4 Å MS (200 mg) in dry DCM (2 ml) was added electron deficient pyridinium salt (0.015 mmol, 0.1 equiv). After stirring for 10 min at room temperature, glycosyl donor (0.15mmol, 1.0 equiv) dissolved in dry DCM (1 ml) was slowly added to the reaction mixture. The resulting reaction mixture was stirred at room temperature until total consumption of glycosyl donor monitored by TLC. After completion of reaction, the mixture was concentrated in vacuo and purified by column chromatography using different fractions of acetone in hexane as eluting solvent to afford the desired glycosides.

Donors (**1*α*** [1,2], **6*α*** [2], **7*α*** [2] and **8*α*** [2]), acceptors (**2n** [3] and **22** [4]), electron-deficient pyridinium salts (**3a–c**) [5] and 1,3-bis-(3,5-bis(trifluoromethyl)phenyl)thiourea **4** [6] were prepared using the procedures already published in literature. NMR data of known compounds were consistent with the literature.

Synthesis of pyrdinium salts:

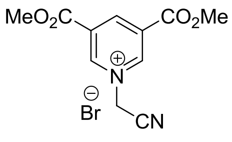

3a

Following the literature procedure [5], compound **3a** was synthesized from dimethyl pyridine-3,5-dicarboxylate (585 mg, 3 mmol, 1 equiv) and 1-bromoacetonitrile (836 µl, 12 mmol, 4 equiv) as a yellow crystalline solid (yield: 602 mg, 85%), mp: 147 °C (decomp.). ^{1}H NMR (400 MHz, DMSO-d_6) δ 10.02 (d, J = 1.2 Hz, 2H), 9.21 (d, J = 1.2 Hz, 1H), 6.17 (s, 2H), 4.02

(s, 6H); ^{13}C NMR (100 MHz, DMSO-d_6) δ 161.7, 150.2, 146.2, 130.6, 114.3, 54.4, 48.7.

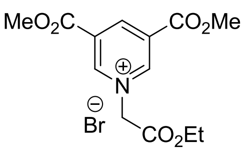

3b

Following the literature procedure [5], compound **3b** was synthesized from dimethyl pyridine-3,5-dicarboxylate (390 mg, 2 mmol, 1 equiv) and ethyl bromoacetate (856 µl, 8 mmol, 4 equiv) as a yellow crystalline solid (yield: 637 mg, 88%), mp: 158 °C (decomp.), ^{1}H NMR (400 MHz, DMSO-d_6) δ 9.92 (d, *J* = 1.6 Hz, 2H), 9.23 (t, *J* = 1.6 Hz, 1H), 5.89 (s, 2H), 4.25 (q, *J* = 7.2 Hz, 2H), 4.02 (s, 6H), 1.27 (t, *J* = 7.2 Hz, 3H); ^{13}C NMR (100 MHz, DMSO-d_6) δ 166.4, 161.8, 150.8, 145.9, 130.0, 63.0, 61.3, 54.3, 14.4.

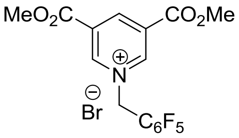

3c

Following the literature procedure [5], compound **3c** was synthesized from dimethyl pyridine-3,5-dicarboxylate (390 mg, 2 mmol, 1 equiv) and pentafluorobenzyl bromide (849 µl, 6 mmol, 3 equiv) as a yellow crystalline solid (yield: 526 mg, 58%), mp: 140 °C (decomp.), ^{1}H NMR (400 MHz, DMSO-d_6) δ 9.74 (d, *J* = 1.6 Hz, 2H), 9.20 (t, *J* = 1.6 Hz, 1H), 6.37 (s, 2H), 4.02 (s, 6H); ^{13}C NMR (100 MHz, DMSO-d_6) δ 161.8, 149.8, 145.7, 130.7, 54.4, 52.9; ^{19}F NMR (376 MHz, DMSO-d_6) δ -139.20 (dd, *J* = 23.8, 6.6 Hz, 2F), -152.05 (t, *J* = 22.2 Hz, 1F), -161.55 – -162.03 (m, 2F).

Synthesis of 1,3-dis-(3,5-bis(trifluoromethyl)phenyl)thiourea 4:

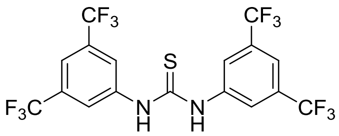

4

Following the literature procedure [6], compound **4** was obtained as a white crystalline solid, mp: 173 °C (decomp.), ^{1}H NMR (400 MHz, DMSO-d_6) δ 10.65 (s, 2H), 8.21 (s, 4H), 7.85 (s, 2H); ^{13}C

NMR (100 MHz, DMSO-d_6) δ 181.0 , 141.6, 131.0 (q, $J_{C\text{-}F}$ = 33 Hz), 124.5, 123.1 (q, $J_{C\text{-}F}$ = 271 Hz),118.1.

Isopropyl 2,3,4,6-tetra-*O*-benzyl-α/β-D-glucopyranoside (5a) [7]**:** The product was isolated from the reaction between glycosyl imidate **1*α*** (103 mg, 0.15 mmol, 1 equiv) and isopropanol **2a** (13 µl, 0.165 mmol, 1.1 equiv) following the general procedure 2(a) and was obtained as semi solid (yield 79 mg, 90%, α/β 2.2:1). R_f: 0.3 (acetone/hexane 1:12 (v/v)), $[\alpha]_D^{29}$ = +42.36 (c = 0.2, CH_2Cl_2); ^1H NMR (400 MHz, $CDCl_3$): δ 7.39– 7.23 (m, 26.2H), 7.17 (dd, J = 7.1, 2.0 Hz, 1.0H), 7.13 (dd, J = 7.0, 2.1 Hz, 1.9H), 5.02 – 4.90 (m, 1.9H), 4.87 (d, J = 3.6 Hz, 1.0H), 4.85 – 4.74 (m, 3.8H), 4.70 (d, J = 10.9 Hz, 0.5H), 4.66 (s, 0.6H), 4.62 (d, J = 2.9 Hz, 0.9H), 4.59 (d, J = 1.7 Hz, 1.0H), 4.56 – 4.52 (m, 1.1H), 4.48 (s, 1.3H), 4.45 (d, J = 3.3 Hz, 1.1H), 4.06 – 3.96 (m, 1.5H), 3.92 – 3.81 (m, 2.0H), 3.75 (d, J = 3.2 Hz, 0.6H), 3.72 (d, J = 3.0, 0.9H), 3.67 – 3.60 (m, 2.8H), 3.57 – 3.51 (m, 1.5H), 3.47 – 3.40 (m, 1.0H), 1.31 (d, J = 6.2 Hz, 1.4H), 1.24 (dd, J = 11.5, 4.6 Hz, 4.7H), 1.17 (d, J = 6.1 Hz, 3.0H); ^{13}C NMR (100 MHz, $CDCl_3$) δ 139.0, 138.7, 138.5, 138.3, 138.2, 138.1, 138.0, 128.6, 128.5, 128.4, 128.3, 128.2, 128.0, 127.9, 127.7, 127.6, 102.2, 94.8, 84.9, 82.3, 82.2, 78.0, 77.9, 75.7, 75.2, 75.0, 74.9, 74.8, 73.5, 73.2, 72.4, 70.0, 69.2, 69.0, 68.5, 23.8, 23.2, 22.3, 21.2.

Allyl 2,3,4,6-tetra-*O*-benzyl-α/β-D-glucopyranoside (5b) [7]**:** The product was isolated from the reaction between glycosyl imidate **1*α*** (103 mg, 0.15 mmol, 1 equiv) and allyl alcohol **2b** (12 µl, 0.165 mmol, 1.1 equiv) following the general procedure 2(a) and was obtained as semi solid (yield: 77 mg, 88%, α/β 1.2:1). R_f: 0.3 (acetone/hexane 1:12 (v/v), $[\alpha]_D^{29}$ = +49.51 (c = 0.1, CH_2Cl_2); ^1H NMR (400 MHz, $CDCl_3$): δ 7.37 – 7.23 (m, 22.6H), 7.18 – 7.10 (m, 2.5H), 6.03 – 5.87 (m, 1.2H), 5.33 (dt, J = 15.6, 1.6 Hz, 1.2H), 5.21 (dd, J = 10.4, 1.3 Hz, 1.1H), 5.02 – 4.91 (m, 1.7H), 4.84 (s, 0.3H), 4.83 (d, J = 1.6 Hz, 1.0H), 4.82 – 4.71 (m, 3.1H), 4.67 (s, 0.5H), 4.63

(d, J = 3.2 Hz, 0.7H), 4.60 (d, J = 2.4 Hz, 0.8H), 4.58 – 4.49 (m, 1.4H), 4.48 – 4.46 (m, 1.1H), 4.44 (s, 0.8H), 4.19 – 4.11 (m, 1.2H), 4.05 – 3.69 (m, 1.4H), 3.83 – 3.42 (m, 7.4H); ^{13}C NMR (100 MHz, $CDCl_3$) δ 138.9, 138.6, 138.5, 138.3, 138.2, 138.1, 138.0, 134.2, 133.8, 128.5, 128.4, 128.3, 128.2, 128.1, 128.0, 127.9, 127.8, 127.7, 127.6, 118.3, 117.3, 102.7, 95.7, 84.7, 82.3, 82.1, 79.9, 77.9, 77.7, 75.8, 75.1, 74.9, 73.5, 73.3, 70.4, 70.3, 69.0, 68.5, 68.2.

Benzyl 2,3,4,6-tetra-*O*-benzyl-α/β-D-glucopyranoside (5c) [7]**:** The product was isolated from the reaction between glycosyl imidate **1α** (103 mg, 0.15 mmol, 1 equiv) and benzyl alcohol **2c** (17 µl, 0.165 mmol, 1.1 equiv) following the general procedure 2(a) and was obtained as semi solid (yield: 83 mg, 88%, α/β 2:1). R_f: 0.3 (acetone/hexane 1:12 (v/v), $[\alpha]_D^{29}$ = +37.37 (c = 0.2, CH_2Cl_2); ^{1}H NMR (400 MHz, $CDCl_3$): δ 7.42 – 7.24 (m, 17.7H), 7.18 – 7.11 (m, 1.4H), 5.00 (dd, J = 11.1, 6.3 Hz, 0.8H), 4.94 (d, J = 3.2 Hz, 0.3H), 4.91 (s, 0.2H), 4.84 (d, J = 4.4 Hz, 1.0H), 4.82 – 4.79 (m, 0.7H), 4.77 (s, 0.1H), 4.74 (s, 0.1H), 4.72 – 4.67 (m, 0.7H), 4.68 – 4.65 (m, 0.7H), 4.63 (s, 0.4H), 4.61 – 4.57 (m, 1.0H), 4.56 – 4.52 (m, 0.9H), 4.50 (d, J = 6.0 Hz, 0.2H), 4.48 (s, 0.2H), 4.45 (d, J = 2.6 Hz, 0.5H), 4.04 (t, J = 9.3 Hz, 0.4H), 3.83 – 3.78 (m, 0.5H), 3.76 (d, J = 1.9 Hz, 0.2H), 3.72 (d, J = 3.6 Hz, 0.4H), 3.70 – 3.67 (m, 0.5H), 3.66 – 3.61 (m, 0.8H), 3.60 – 3.45 (m, 1.6H); ^{13}C NMR (100 MHz, $CDCl_3$) δ 138.9, 138.7, 138.5, 138.3, 138.2, 138.0, 137.5, 137.2, 128.5, 128.4, 128.3, 128.0, 127.8, 127.7, 102.7, 95.7, 84.8, 82.4, 82.2, 79.9, 77.9, 77.8, 75.8, 75.2, 75.1, 75.0, 73.5, 73.1, 71.2, 70.4, 69.2, 69.0, 68.4.

4-Methoxybenzyl 2,3,4,6-tetra-*O*-benzyl-α/β-D-glucopyranoside (5d) [7]**:** The product was isolated from the reaction between glycosyl imidate **1α** (103 mg, 0.15 mmol, 1 equiv) and 4-methoxybenzyl alcohol **2d** (21 µl, 0.165 mmol, 1.1 equiv) following the general procedure 2(a) and was obtained as sticky liquid (yield: 81 mg, 82%, α/β 3.3:1). R_f: 0.3 (acetone/hexane 1:12 (v/v), $[\alpha]_D^{29}$= +36.20 (c = 0.1, CH_2Cl_2); ^{1}H NMR (400 MHz, $CDCl_3$) δ 7.39 – 7.22 (m, 19.4H),

7.19 – 7.11(m, 1.9H), 6.86 (dd, J = 8.8, 2.5 Hz, 1.8H), 5.02 – 4.88 (m, 1.7H), 4.84(d, J = 3.6 Hz,, 1.0H), 4.83 – 4.79 (m, 1.3H), 4.76 (s, 0.1H), 4.72 (s, 0.2H), 4.70 – 4.58 (m, 2.9H), 4.56 (d, J = 3.3 Hz, 0.7H), 4.53 (d, J = 4.4 Hz, 0.3H), 4.52 – 4.46 (m, 2.0H), 4.03 (t, J = 9.3 Hz, 0.6H), 3.81 (s, 2.0H), 3.80 (s, 1.0H), 3.76 (d, J = 1.9 Hz, 0.2H), 3.74 – 3.68 (m, 1.0H), 3.66 (d, J = 10.1 Hz, 0.6H), 3.63 – 3.52 (m, 2.0H), 3.50 (d, J = 8.8 Hz, 0.3H), 3.46 (dd, J = 6.4, 3.0 Hz, 0.2H); ^{13}C NMR (100 MHz, $CDCl_3$) δ 159.7, 139.3, 139.0, 138.8, 138.7, 138.6, 138.5, 138.4, 130.5, 130.0, 129.9, 129.6, 128.7, 128.3, 128.2, 128.1, 128.0, 127.9, 114.2, 114.1, 102.7, 95.5, 85.1, 82.6, 82.5, 80.3, 78.3, 78.1, 77.4, 76.0, 75.4, 75.3, 75.1, 73.8, 73.2, 71.2, 70.7, 69.4, 69.1, 68.9, 55.6.

Cyclohexyl 2,3,4,6-tetra-*O*-benzyl-α/β-D-glucopyranoside (5e) [7]**:** The product was isolated from the reaction between glycosyl imidate **1α** (103 mg, 0.15 mmol, 1 equiv) and cyclohexanol **2e** (17 µl, 0.165 mmol, 1.1 equiv) following the general procedure 2(a) and was obtained as semi solid (yield: 73 mg, 78%, α/β 2.1:1). R_f: 0.3 (acetone/hexane 1:12 (v/v), $[\alpha]_D^{29}$= +46.83 (c = 0.1, CH_2Cl_2); ^{1}H NMR (400 MHz, $CDCl_3$) δ 7.40 – 7.22 (m, 36.1H), 7.19 – 7.11 (m, 3.7H), 5.00 (d, J = 10.8 Hz, 1.7H), 4.95 (d, J = 3.6 Hz, 1.0H), 4.92 (d, J = 11.0 Hz, 0.6H), 7.86 – 4.77 (m, 3.4H), 4.77 – 4.67 (m, 2.3H), 4.68 – 4.49 (m, 5.7H), 4.46 (d, J = 11.3 Hz, 2.3H), 4.00 (t, J = 9.3 Hz, 1.1H), 3.88 (d, J = 10.6 Hz, 1.2H), 3.74 (dd, J = 10.6, 3.5 Hz, 2.7H), 3.64 (t, J = 9.3 Hz, 3.5H), 3.60 – 3.48 (m, 3.2H), 3.48 (s, 0.3H), 3.45 (d, J = 8.0 Hz, 0.5H), 3.42 (s, 0.1H), 2.06-1.67 (m, 7.4H), 1.64 – 1.14 (m, 13.1H); ^{13}C NMR (100 MHz, $CDCl_3$) δ 139.0, 138.7, 138.6, 138.3, 138.2, 138.0, 128.4, 128.3, 128.2, 128.1, 128.0, 127.9, 127.8, 127.7, 127.6, 102.0, 94.7, 84.9, 82.3, 82.1, 78.0, 77.8, 75.7, 75.3, 75.2, 75.0, 74.9, 74.8, 73.5, 73.0, 70.1, 69.2, 68.6, 33.9, 33.4, 32.1, 31.5, 25.7, 24.5, 24.2, 24.0.

Cyclopentyl 2,3,4,6-tetra-*O*-benzyl-α/β-D-glucopyranoside (5f): The product was isolated from the reaction between glycosyl imidate **1α** (103 mg, 0.15 mmol, 1 equiv) and cyclopentanol

2f (15 µl, 0.165 mmol, 1.1 equiv) following the general procedure 2(a) and was obtained as semi solid (yield: 78 mg, 85%, α/β 2.5:1). R_f: 0.3 (acetone/hexane 1:12 (v/v),$[\alpha]_D^{29}$= +39.63 (c = 0.1, CH_2Cl_2); ^{1}H NMR (400 MHz, $CDCl_3$) δ 7.35 – 7.20 (m, 24.7H), 7.12 (dd, J = 7.2, 2.3 Hz, 1.1H), 7.09 (dd, J = 7.1, 2.4 Hz, 1.4H), 4.98 – 4.87 (m, 1.7H), 4.81 (d, J = 3.7 Hz, 1.0H), 4.80 – 4.75 (m, 2.0H), 4.74 – 4.62 (m, 1.7H), 4.62 – 4.54 (m, 2.5H), 4.52 – 4.46 (m, 0.9H), 4.45 – 4.33 (m, 2.6H), 4.15 – 4.08 (m, 0.8H), 3.94 (t, J = 9.3 Hz, 0.8H), 3.80 – 3.75 (m, 0.8H), 3.71 (dd, J = 10.5, 3.2 Hz, 1.3H), 3.61 (m, 2.7H), 3.55 – 3.48 (m, 1.3H), 3.45 – 3.42 (m, 0.4H), 3.40 (d, J = 3.7 Hz, 0.4H), 3.37 (s, 0.1H), 1.91 – 1.67 (m, 6.4H), 1.56 – 1.46 (m, 3H); ^{13}C NMR (100 MHz, CDC_{l3}) δ 139.1, 138.7, 138.5, 138.4, 138.3, 138.2, 138.0, 128.4, 128.2, 128.1, 128.0, 127.9, 127.7, 127.6, 102.1, 95.7, 84.9, 82.3, 82.1, 81.0, 80.1, 78.9, 78.0, 77.9, 75.7, 75.2, 75.0, 74.9, 73.5, 73.0, 70.1, 69.2, 68.6, 33.7, 32.9, 32.2, 31.7, 23.7, 23.5, 23.4, 23.3. HRMS (ESI-TOF): calculated for $C_{39}H_{44}NaO_6$ $[M+Na]^+$ 631.3036 found 631.3034.

2-Bromoethyl 2,3,4,6-tetra-*O*-benzyl-β-D-glucopyranoside (5g): The product was isolated from the reaction between glycosyl imidate **1α** (103 mg, 0.15 mmol, 1 equiv) and 2-bromoethanol **2g** (12 µl, 0.165 mmol, 1.1 equiv) following the general procedure 2(a) and was obtained as semi solid (yield: 70 mg, 72%, β only). R_f: 0.3 (acetone/hexane 1:12 (v/v), $[\alpha]_D^{29}$ = +43.65 (c = 0.1, CH_2Cl_2); ^{1}H NMR (400 MHz, $CDCl_3$) δ 7.36 – 7.11 (m, 19.7H), 7.09 – 7.00 (m, 1.9H), 5.88 (d, J = 5.3 Hz, 0.5H), 4.97 – 4.82 (m, 0.9H), 4.81 – 4.68 (m, 2.4H), 4.67 – 4.54 (m, 1.3H), 4.53 – 4.41 (m, 2.9H), 4.37 (d, J = 8.1 Hz, 1.0H), 4.36 – 4.29 (m, 0.6H), 4.20 – 4.13 (m, 0.4H), 3.85 – 3.75 (m, 1.1H), 3.68 – 3.51 (m, 3.0H), 3.50 – 3.36 (m, 2.5H), 3.28 (t, J = 9.4 Hz, 0.6H); ^{13}C NMR (100 MHz, $CDCl_3$) δ 138.6, 138.4, 138.0, 137.3, 136.7, 136.6, 128.6, 128.5, 128.4, 128.3, 128.2, 128.1, 128.0, 127.9, 127.8, 127.7, 103.7, 84.56, 83.4, 82.1, 81.8, 78.7, 77.7,

76.0, 75.8, 75.3, 75.2, 75.1, 74.9, 73.5, 73.3, 72.9, 71.8, 69.7, 68.8, 68.6, 30.3. HRMS (ESI-TOF): calculated for $C_{36}H_{39}BrNaO_6$ $[M+Na]^+$ 669.1828 found 669.1809.

3-Chloropropyl 2,3,4,6-tetra-*O*-benzyl-α/β-D-glucopyranoside (5h): The product was isolated from the reaction between glycosyl imidate **1α** (103 mg, 0.15 mmol, 1 equiv) and 3-chloropropanol **2h** (14 µl, 0.165 mmol, 1.1 equiv) following the general procedure 2(a) and was obtained as semi solid (yield: 65 mg, 70%, α/β 1.1:1). R_f: 0.3 (acetone/hexane 1:12 (v/v), $[\alpha]_D^{29}$ = +35.76 (c = 0.2, CH_2Cl_2); ^1H NMR (400 MHz, $CDCl_3$) δ 7.39 – 7.24 (m, 26.7H), 7.18 – 7.13 (m, 2.7H), 5.01 – 4.89 (m, 2.1H), 4.86 – 4.78 (m, 3.1H), 4.76 (d, *J* = 3.7 Hz, 1.0H), 4.73 (d, *J* = 11.0 Hz, 0.7H), 4.67 – 4.60 (m, 2.1H), 4.58 – 4.46 (m, 3.1H), 4.41 (d, *J* = 7.8 Hz, 0.7H), 4.09 – 4.04 (m, 0.7H), 3.97 (t, *J* = 9.3 Hz, 0.7H), 3.87 – 3.78 (m, 1.0H), 3.78 – 3.62 (m, 8.2H), 3.62 – 3.48 (m, 2.5H), 3.46 (d, *J* = 8.4 Hz, 0.9H), 3.43 (s, 0.2H), 2.18 – 1.99 (m, 2.7H).; ^{13}C NMR (100 MHz, $CDCl_3$) δ 185.6, 139.2, 138.9, 138.5, 138.4, 138.2, 128.8, 128.7, 128.4, 128.4, 128.3, 128.2, 128.1, 128.0, 127.9, 103.9, 97.6, 85.0, 82.6, 82.4, 80.5, 78.1, 78.0, 76.0, 75.5, 75.3, 75.2, 73.9, 73.6, 70.6, 69.2, 68.8, 66.7, 64.7, 42.2, 42.1, 33.1, 32.6. HRMS (ESI-TOF): calculated for $C_{37}H_{41}ClNaO_6$ $[M+Na]^+$ 639.2489 found 639.2491.

1-Adamantylmethyl 2,3,4,6-tetra-*O*-benzyl-α/β-D-glucopyranoside (5i): The product was isolated from the reaction between glycosyl imidate **1α** (103 mg, 0.15 mmol, 1 equiv) and 1-adamentanemethanol **2i** (27 mg, 0.165 mmol, 1.1 equiv) following the general procedure 2(a) and was obtained as semi solid (yield: 78 mg, 75%, α/β 3.3:1). R_f: 0.4 (acetone/hexane 1:10 (v/v), $[\alpha]_D^{29}$= +32.68 (c = 0.1, CH_2Cl_2); ^1H NMR (400 MHz, $CDCl_3$) δ 7.43 – 7.26 (m, 18.7H), 7.22 – 7.14 (m, 2.1H), 5.08 – 4.95 (m, 1.2H), 4.86 (dd, *J* = 10.8, 7.6 Hz, 1.9H), 4.77 (m, 1.0H), 4.80 – 4.72 (m, 1.8H), 4.64 (m, 2H), 4.59 – 4.49 (m, 1.7H), 4.38 (d, *J* = 7.8 Hz, 0.3H), 4.38 (d, J = 7.8 Hz, 0.3H), 4.01 (t, *J* = 9.2 Hz, 0.7H), 3.82 – 3.74 (m, 1.7H), 3.72 – 3.58 (m, 3.4H), 3.53 –

3.43 (m, 0.7H), 3.30 (d, *J* = 9.2 Hz, 0.7H), 3.12 (d, *J* = 9.5 Hz, 0.3H), 2.91 (d, *J* = 9.2 Hz, 0.7H), 2.00 (s, 3.0H), 1.79 – 1.58 (m, 13.7H); ^{13}C NMR (100 MHz, $CDCl_3$) δ 139.0, 138.7, 138.6, 138.5, 138.3, 138.1, 138.0, 128.4, 128.2, 128.1, 128.0, 127.9, 127.8, 127.7, 127.6, 127.5, 104.3, 97.3, 84.8, 82.3, 82.0, 80.8, 80.7, 78.9, 78.0, 77.8, 75.8, 75.6, 75.2, 75.0, 74.9, 73.5, 72.6, 69.9, 68.9, 68.6, 39.6, 37.1, 33.9, 33.8, 28.2. HRMS (ESI-TOF): calculated for $C_{45}H_{52}NaO_6$ $[M+Na]^+$ 711.3662 found 711.3663.

1-Adamantyl 2,3,4,6-tetra-*O*-benzyl-α/β-D-glucopyranoside (5j) [8]**:** The product was isolated from the reaction between glycosyl imidate **1α** (103 mg, 0.15 mmol, 1 equiv) and 1-adamentanol **2j** (25 mg, 0.165 mmol, 1.1 equiv) following the general procedure 2(a) and was obtained as semi solid (yield: 44 mg, 43%, *α/β* 2:1). R_f: 0.4 (acetone/hexane 1:10 (v/v), $[\alpha]_D^{27}$= +48.56 (c = 0.1, CH_2Cl_2); ^{1}H NMR (400 MHz, $CDCl_3$) δ 7.41 – 7.24 (m, 31.2H), 7.21 (dd, *J* = 7.4, 2.0 Hz, 1.0H), 7.16 (dd, *J* = 7.2, 2.2 Hz, 2.1H), 5.30 (d, *J* = 3.6 Hz, 1.0H), 5.02 (dd, *J* = 10.9, 7.6 Hz, 1.5H), 4.94 (d, *J* = 10.9 Hz, 0.5H), 4.89 – 4.77 (m, 3.2H), 4.74 (d, *J* = 6.4 Hz, 0.6H), 4.71 (s, 2.3H), 4.69 – 4.54 (m, 3.1H), 4.48 (dd, *J* = 11.4, 4.2 Hz, 2.0H), 4.08 – 4.00 (m, 2.0 H), 3.77 (td, *J* = 10.3, 2.4 Hz, 1.7H), 3.71 – 3.61 (m, 3.3H), 3.59 – 3.53 (m, 1.5H), 3.52 – 3.43 (m, 1.4H), 2.16 (d, *J* = 2.3 Hz, 5.9H), 2.01 – 1.73 (m, 11.3H), 1.70 – 1.57 (m, 11.0H); ^{13}C NMR (100 MHz, $CDCl_3$) δ 139.1, 138.7, 138.6, 138.4, 138.3, 138.2, 138.1, 128.4, 128.3, 128.2, 128.0, 127.9, 127.8, 127.7, 127.6, 127.5, 127.4, 96.2, 89.9, 85.1, 82.3, 82.0, 80.1, 78.2, 78.1, 75.7, 75.5, 75.3, 75.1, 74.9, 74.6, 73.4, 72.9, 69.7, 68.7, 42.8, 42.4, 36.3, 30.7, 30.6.

(1*S*,2*R*,5*S*)-Menthyl 2,3,4,6-tetra-*O*-benzyl-α-D-glucopyranoside (5k) [9]**:** The product was isolated from the reaction between glycosyl imidate **1α** (103 mg, 0.15 mmol, 1 equiv) and (+)-menthol **2k** (26 mg, 0.165 mmol, 1.1 equiv) following the general procedure **2(a)** and was obtained as sticky liquid (yield: 62 mg, 61%, α only). R_f: 0.6 (acetone/hexane 1:10 (v/v),$[\alpha]_D^{27}$ =

+15.60 (c = 0.1, CH_2Cl_2); ^{1}H NMR (400 MHz, $CDCl_3$) δ 7.44 – 7.25 (m, 20.6H), 7.22 – 7.14 (m, 2.1H,), 5.05 (d, *J* = 3.6 Hz, 1.0H), 5.01 (d, *J* = 10.9 Hz, 1.0H), 4.89-4.83 (m, 2.0H), 4.78 – 4.64 (m, 3.1H), 4.53 – 4.46(m, 2.1H,), 4.08 – 3.97 (m, 2.0H), 3.79 (dd, *J* = 10.5, 3.8 Hz, 1.0H), 3.67 (m, 2.2H), 3.58 (dd, *J* = 9.8, 3.6 Hz, 1.0H), 3.38 (td, J = 10.6, 4.4 Hz, 1.0H), 2.50 – 2.40 (m, 1.0H), 2.16 (d, *J* = 12.1 Hz, 1.0H), 1.70 – 1.57 (m, 2.6H), 1.48 – 1.26 (m, 2.8H), 1.12 – 0.79 (m, 10.2H), 0.74 (d, *J* = 6.9 Hz, 3.1H); ^{13}C NMR (100 MHz, $CDCl_3$) δ 138.9, 138.4, 138.3, 138.1, 128.4, 128.3, 127.9, 127.6, 127.5, 98.7, 82.0, 81.0, 80.6, 78.1, 75.5, 73.5, 73.2, 70.3, 68.7, 48.8, 43.1, 34.3, 31.7, 24.6, 23.0, 22.3, 21.1, 16.1.

(1*R*,2*S*,5*R*)-Menthyl 2,3,4,6-tetra-*O*-benzyl-α/β-D-glucopyranoside (5l) [9]**:** The product was isolated from the reaction between glycosyl imidate **1α** (103 mg, 0.15 mmol, 1 equiv) and (−)-menthol **2l** (26 mg, 0.165 mmol, 1.1 equiv) following the general procedure 2(a) and was obtained as sticky liquid (yield: 68 mg, 67%, α/β 5:1). R_f: 0.6 (acetone/hexane 1:10 (v/v),$[\alpha]_D^{27}$ = +38.51 (c = 0.2, CH_2Cl_2); ^{1}H NMR (400 MHz, $CDCl_3$) δ 7.34 – 7.12 (m, 21.7H), 7.05 (d, *J* = 2.6 Hz, 2.0H), 4.96 – 4.82 (m, 2.3H), 4.79 – 4.66 (m, 3.6H), 4.62 – 4.47 (m, 2.7H), 4.44 – 4.32 (m, 2.2H), 3.87 (t, *J* = 9.3 Hz, 1.0H), 3.78 – 3.66 (m, 2.1H), 3.65 – 3.55 (m, 1.5H), 3.53 – 3.45 (m, 2.1H), 3.44 – 3.30 (m, 1.7H), 2.26 – 2.15 (m, 1.4H), 1.90 (d, *J* = 11.4 Hz, 1.0H), 1.63 – 1.48 (m, 3.1H), 1.41 – 1.15 (m, 3.2H), 0.93 – 0.69 (m, 11.4H), 0.62 (d, *J* = 6.5 Hz, 3.6H); ^{13}C NMR (100 MHz, $CDCl_3$) δ 139.0, 138.6, 138.4, 138.2, 138.1, 138.0, 128.5, 128.4, 128.3, 128.2, 128.0, 127.9, 127.8, 127.7, 127.6, 127.5, 104.5, 93.4, 85.1, 82.8, 82.1, 81.2, 80.0, 78.2, 77.9, 75.8, 75.7, 75.5, 75.3, 75.1, 75.0, 74.8, 73.7, 73.6, 73.5, 70.7, 69.3, 68.5, 48.9, 47.3, 43.7, 39.8, 34.4, 34.3, 31.8, 31.5, 25.1, 22.8, 22.7, 22.5, 22.4, 21.4, 21.2, 16.0, 15.2.

Cholesteryl 2,3,4,6-tetra-*O*-benzyl-α/β-D-glucopyranoside (5m) [9]**:** The product was isolated from the reaction between glycosyl imidate **1α** (103 mg, 0.15 mmol, 1 equiv) and cholesterol **2m**

(64 mg, 0.165 mmol, 1.1 equiv) following the general procedure 2(a) and was obtained as white solid (yield: 82 mg, 60%, α/β 10.1:1). R_f: 0.5 (acetone/hexane 1:10 (v/v), $[\alpha]_D^{29}$ = +40.85 (c = 0.2, CH_2Cl_2); ^{1}H NMR (400 MHz, $CDCl_3$) δ 7.44 – 7.26 (m, 21.3H), 7.20 (dd, J = 6.2, 3.1 Hz, 0.4H), 7.16 (dd, J = 6.3, 3.0 Hz, 1.9H), 5.38 (d, J = 5.1 Hz, 0.1H), 5.32 (d, J = 5.0 Hz, 0.9H), 5.04 (d, J = 10.8 Hz, 1.0H), 4.99 (s, 0.1H), 4.96 (d, J = 3.5 Hz, 1.0H), 4.94 (s, 0.1H), 4.80 – 4.72 (m, 3.5H), 4.71 – 4.54 (m, 2.5H), 4.52 (s, 0.1H), 4.49 (dd, J = 11.3, 4.1 Hz, 1.9H), 4.04 (t, J = 9.3 Hz, 1.0H), 3.91 (d, J = 9.2 Hz, 1.0H), 3.81 – 3.45 (m, 6.2H), 2.61 – 2.26 (m, 2.3H), 2.08 – 1.81 (m, 5.7H), 1.64 – 0.88 (m, 41.9H), 0.71 (s, 3.0H); ^{13}C NMR (100 MHz, $CDCl_3$) δ 140.8, 139.0, 138.3, 138.2, 138.0, 128.4, 128.2, 128.0, 127.9, 127.7, 127.6, 121.7, 102.2, 94.6, 82.1, 79.9, 77.9, 76.5, 75.7, 75.2, 73.4, 73.1, 70.0, 68.6, 56.8, 56.2, 50.1, 42.3, 39.9, 39.8, 39.5, 37.1, 36.8, 36.2, 35.8, 31.9, 28.3, 28.0, 27.5, 24.3, 23.8, 22.9, 21.1, 19.4, 18.7, 11.9.

6-*O*-(2,3,4,6-Tetra-*O*-benzyl-α/β-D-glucopyranosyl)-1,2;3,4-di-*O*-isopropylidene-α-D-galactopyranose (5n) [10]**:** The product was isolated from the reaction between glycosyl imidate **1α** (103 mg, 0.15 mmol, 1 equiv) and glycosly acceptor **2n (**43 mg, 0.165 mmol, 1.1 equiv) following the general procedure 2(a) and was obtained as semi solid (yield: 85 mg, 72%, α/β 1:1.3). R_f: 0.4 (acetone/hexane 1:5 (v/v), $[\alpha]_D^{29}$ = -15.00 (c = 0.1, CH_2Cl_2); ^{1}H NMR (400 MHz, $CDCl_3$) δ 7.42 (dd, J = 7.5, 1.8 Hz, 2.4H), 7.38 – 7.22 (m, 34.9H), 7.13 (dd, J = 7.0, 2.3 Hz, 3.9H), 5.57 (d, J = 5.0 Hz, 1.1H), 5.52 (d, J = 5.2 Hz, 0.5H), 5.05 (d, J = 11.1 Hz, 1.1H), 5.00 (d, J = 1.5 Hz, 1.0H), 4.96 (d, J = 10.9 Hz, 1.5H), 4.84 – 4.68 (m, 6.6H), 4.65 – 4.57 (m, 4.0H), 4.56 – 4.46 (m, 3.9H), 4.45 (d, J = 3.7 Hz, 1.3H), 4.35 (dd, J = 8.0, 1.8 Hz, 0.7H), 4.32 (dd, J = 5.0, 2.5 Hz, 1.8H), 4.25 (dd, J = 7.9, 1.8 Hz, 1.1H), 4.17 (dd, J = 10.6, 3.6 Hz, 1.1H), 4.12 – 4.07 (m, 1.2H), 4.06 – 4.01 (m, 0.7H), 3.98 (t, J = 9.3 Hz, 0.7H), 3.84 – 3.57 (m, 11.5H), 3.49 – 3.41(m, 2.3H), 1.53 (s, 2.0H), 1.50 (s, 3.0H), 1.45 (s, 5.4H), 1.33 (t, J = 6.6 Hz, 11.6H), 1.25 (s, 3.5H);

^{13}C NMR (100 MHz, $CDCl_3$) δ 138.9, 138.7, 138.3, 138.1, 137.9, 128.7, 128.4, 128.2, 128.0, 127.8, 127.7, 127.5, 109.4, 108.6, 104.4, 97.0, 96.4, 84.5, 82.0, 81.6, 79.8, 77.7, 75.7, 75.0, 74.7, 74.4, 73.5, 72.4, 71.4, 70.8, 70.6, 70.5, 70.2, 69.7, 68.7, 68.3, 67.4, 66.2, 65.7, 29.7, 26.2, 26.0, 25.1, 24.5.

Benzyl 2,3,4,6-tetra-*O*-benzyl-α/β-D-galactopyranoside (9) [11]**:** The product was isolated from the reaction between glycosyl donor **6α** (103 mg, 0.15 mmol, 1 equiv) and benzyl alcohol **2c** (17 µl, 0.165 mmol, 1.1 equiv) following the general procedure 2(a) and was obtained as semi solid (yield: 86 mg, 91%, α/β 1.7:1). R_f: 0.3 (acetone/hexane 1:12 (v/v), $[\alpha]_D^{27}$ = +36.72 (c = 0.2, CH_2Cl_2); ^{1}H NMR (400 MHz, $CDCl_3$) δ 7.41 – 7.21 (m, 47.9H), 4.96 – 4.91 (m, 2.8H), 4.89 (d, *J* = 3.2 Hz, 1.0H), 4.84 (d, *J* = 11.6 Hz, 1.0H), 4.77 – 4.69 (m, 4.7H), 4.60 (m, 5.9H), 4.48 – 4.41 (m, 3.6H), 4.37 (m, 1.3H), 4.06 – 3.94 (m, 4.3H), 3.92 – 3.85 (m, 1.5H), 3.61 (m, 1.8H), 3.53 (m, 2.4H), 3.46 (m, 1.0H); ^{13}C NMR (100 MHz, $CDCl_3$) δ 128.8, 128.6, 128.5, 128.2, 128.1, 128.0, 127.9, 127.8, 103.2, 96.4, 82.5, 79.9, 79.5, 76.8, 75.6, 75.4, 75.1, 74.9, 73.9, 73.8, 73.5, 73.4, 71.2, 69.8, 69.3, 69.2.

2-Bromoethyl 2,3,4,6-tetra-*O*-benzyl-α/β-D-galactopyranoside (10): The product was isolated from the reaction between glycosyl donor **6*α*** (103 mg, 0.15 mmol, 1 equiv) and 2-bromoethanol **2g** (12 µl, 0.165 mmol, 1.1 equiv) following the general procedure 2(a) and was obtained as semi solid (yield: 72 mg, 74%, *α/β* 2.1:1). R_f: 0.3 (acetone/hexane 1:12 (v/v), $[\alpha]_D^{29}$= +35.00 (c = 0.2, CH_2Cl_2); ^{1}H NMR (400 MHz, $CDCl_3$) δ 7.46 – 7.27 (m, 18.5H), 5.04 (d, *J* = 10.8 Hz, 0.3H), 5.00 – 4.98 (m, 0.4H), 4.97 (d, *J* = 3.4 Hz, 0.4H), 4.91 (s, 0.2H), 4.88 (d, *J* = 4.3 Hz, 1.0H), 4.86 (s, 0.3H), 4.83 – 4.75 (m, 1.6H), 4.74 – 4.60 (m, 1.6H), 4.54 – 4.49 (m, 0.7H), 4.47 – 4.42 (m, 1.4H), 4.27 – 4.21(m, 0.7H), 4.11 (d, *J* = 3.6 Hz, 0.1H), 4.08 (d, *J* = 8.0 Hz, 0.1H), 4.07 (s, 0.2H), 4.04 – 3.98 (m, 1.1H), 3.96 – 3.84 (m, 2.1H), 3.62 (d, *J* = 2.4 Hz, 0.3H), 3.60 – 3.52 (m,

3.7H); ^{13}C NMR (100 MHz, $CDCl_3$) δ 138.8, 138.6, 138.5, 138.0, 137.8, 128.5, 128.4, 128.3, 128.1, 127.8, 127.6, 127.5, 104.0, 98.1, 82.0, 79.4, 79.0, 76.5, 75.3, 75.0, 74.8, 74.6, 73.6, 73.5, 73.3, 73.2, 69.8, 69.6, 69.0, 68.8, 68.3, 30.4, 30.3. HRMS (ESI-TOF): calculated for $C_{36}H_{43}BrNO_6$ $[M+NH_4]^+$ 664.2274 found 664.2274.

3-Chloropropyl 2,3,4,6-tetra-*O*-benzyl-α/β-D-galactopyranoside (11): The product was isolated from the reaction between glycosyl donor **6α** (103 mg, 0.15 mmol, 1 equiv) and 3-chloropropanol **2h** (14 µl, 0.165 mmol, 1.1 equiv) following the general procedure 2(a) and was obtained as semi solid (yield: 75 mg, 81%, α/β 1:4.6). R_f: 0.3 (acetone/hexane 1:12 (v/v), $[\alpha]_D^{29}$= +17.92 (c = 0.1, CH_2Cl_2); ^{1}H NMR (400 MHz, $CDCl_3$) δ 7.44 – 7.23 (m, 33.3H), 4.96 (dd, J = 11.5, 6.8 Hz, 1.5H), 4.91 – 4.82 (m, 2.5H), 4.81 – 4.74 (m, 3.5H), 4.73 – 4.54 (m, 3.0H), 4.53 – 4.44 (m, 2.9H), 4.39 (d, J = 7.7 Hz, 1.0H), 4.11 – 3.99 (m, 2.3H), 3.98 – 3.89 (m, 2.0H), 3.87 – 3.79 (m, 1.7H), 3.76 – 3.51 (m, 10.4H), 2.17 – 1.97 (m, 3.3H); ^{13}C NMR (100 MHz, $CDCl_3$) δ 138.8, 138.6, 138.4, 138.0, 137.8, 128.5, 128.4, 128.3, 128.2, 128.1, 127.9, 127.8, 127.7, 127.6, 127.5, 104.0, 97.9, 82.1, 79.6, 79.0, 75.3, 75.0, 74.8, 74.6, 73.6, 73.5, 73.4, 73.2, 73.0, 69.4, 69.0, 68.8, 66.4, 64.6, 41.9, 41.8, 32.8, 32.3. HRMS (ESI-TOF): calculated for $C_{37}H_{45}ClNO_6$ $[M+NH_4]^+$ 634.2935 found 634.2936.

1-Adamantanemethyl 2,3,4,6-tetra-*O*-benzyl-α/β-D-galactopyranoside (12): The product was isolated from the reaction between glycosyl donor **6α** (103 mg, 0.15 mmol, 1 equiv) and 1-adamentanemethanol **2i** (27 mg, 0.165 mmol, 1.1 equiv) following the general procedure **2(a)** and was obtained as semi solid (yield: 75 mg, 73%, α/β 2.6:1). R_f: 0.4 (acetone/hexane 1:10 (v/v), $[\alpha]_D^{29}$= +32.46 (c = 0.1, CH_2Cl_2); ^{1}H NMR (400 MHz, $CDCl_3$) δ 7.45 – 7.22 (m, 20.2H), 5.05 – 4.93 (m, 1.2H), 4.92 – 4.85 (m, 0.6H), 4.83 (d, J = 3.1 Hz, 1.0H), 4.80 – 4.71 (m, 1.8H), 4.70 – 4.59 (m, 1.7H), 4.56 – 4.42 (m, 2.3H), 4.32 (d, J = 7.7 Hz, 0.4H), 4.08 (dd, J = 9.3, 1.9

Hz, 0.8H), 4.01 – 3.65 (m, 2.6H), 3.60 (t, *J* = 5.1 Hz, 1.0H), 3.58 – 3.51 (m, 1.8H), 3.40 (d, *J* = 9.2 Hz, 0.1H), 3.29 (d, *J* = 9.1 Hz, 0.5H), 3.06 (d, *J* = 9.6 Hz, 0.3H), 2.89 (dd, *J* = 16.2, 9.2 Hz, 0.6H), 1.99 (s, 2.8H), 1.79 – 1.52 (m, 12.2H); ^{13}C NMR (100 MHz, $CDCl_3$) δ 139.1, 138.9, 138.7, 138.6, 138.1, 138.0, 128.5, 128.4, 128.3, 128.2, 128.1, 127.9, 127.7, 127.6, 127.5, 127.4, 104.9, 97.8, 82.4, 80.8, 79.6, 78.7, 78.6, 75.3, 75.2, 74.7, 74.5, 73.7, 73.5, 73.4, 73.2, 73.0, 72.6, 69.2, 68.9, 39.7, 37.2, 35.0, 33.9, 28.3, 28.2. HRMS (ESI-TOF): calculated for $C_{45}H_{56}NO_6$ $[M+NH_4]^+$ 706.4108 found 706.4108.

6-*O*-(2,3,4,6-Tetra-*O*-benzyl-α/β-D-galactopyranosyl)-1,2;3,4-di-*O*-isopropylidene-α-D-galactopyranose (13) [10]**:** The product was isolated from the reaction between glycosyl donor **6α** (103 mg, 0.15 mmol, 1 equiv) and glycosyl acceptor **2n** (43 mg, 0.165 mmol, 1.1 equiv) following the general procedure 2(a) and was obtained as semi solid (yield: 73 mg, 62%, *α/β* 1.4:1). R_f: 0.4 (acetone/hexane 1:5 (v/v), $[\alpha]_D^{29}$= -12.36 (c = 0.1, CH_2Cl_2); ^{1}H NMR (400 MHz, $CDCl_3$) δ 7.47 (dd, *J* = 7.6, 1.5 Hz, 1.5H), 7.44 – 7.23 (m, 39.2H), 5.59 (d, *J* = 5.0 Hz, 0.6H), 5.54 (d, *J* = 5.0 Hz, 1.0H), 5.09 – 5.00 (m, 1.6H), 4.95 (dd, *J* = 11.5, 2.2 Hz, 1.7H), 4.88 – 4.81 (m, 1.4H), 4.80 – 4.71 (m, 5.4H), 4.67 – 4.55 (m, 4.1H), 4.54 – 4.40 (m, 5.1H), 4.37 – 4.30 (m, 3.0H), 4.24 (dd, *J* = 7.9, 1.7 Hz, 0.8H), 4.15 (dd, *J* = 10.5, 3.6 Hz, 0.8H), 4.12 – 4.01 (m, 5.0H), 3.98 (dd, *J* = 10.0, 2.7 Hz, 1.2H), 3.91 (d, *J* = 2.6 Hz, 1.0H), 3.87 – 3.65 (m, 4.2H), 3.62 – 3.57 (m, 2.1H), 3.56 – 3.50 (m, 3.0H), 1.54 (s, 2.9H), 1.52 (s, 2.2H), 1.46 (d, *J* = 3.3 Hz, 4.9H), 1.37 – 1.31 (m, 11.4H); ^{13}C NMR (100 MHz, $CDCl_3$) δ 139.0, 138.9, 138.7, 138.6, 138.0, 137.9, 128.6, 128.4, 128.3, 128.2, 127.9, 127.8, 127.7, 127.5, 127.4, 109.2, 108.6, 104.7, 97.5, 96.3, 81.9, 79.1, 79.0, 76.4, 74.9, 74.8, 74.6, 73.5, 73.4, 73.3, 73.1, 72.7, 71.5, 70.9, 70.8, 70.7, 70.6, 70.5, 69.6, 69.2, 68.7, 67.4, 66.3, 65.8, 26.2, 26.0, 25.1, 24.9, 24.6, 24.4.

Benzyl 2,3,4,6-tetra-*O*-benzyl-α/β-D-mannopyranoside (14) [12]**:** The product was isolated from the reaction between glycosyl donor **7α** (103 mg, 0.15 mmol, 1 equiv) and benzyl alcohol (**2c**, 17 µl, 0.165 mmol, 1.1 equiv) following the general procedure 2(a) and was obtained as sticky liquid (yield: 70 mg, 74%, α/β 1.2:1). R_f: 0.3 (acetone/hexane 1:12 (v/v),$[\alpha]_D^{29}$ = -7.84 (c = 0.2, CH_2Cl_2); ^1H NMR (400 MHz, $CDCl_3$): δ 7.44 (dd, J = 6.4, 3.1 Hz, 1.1H), 7.40 – 7.23 (m, 28.9H), 7.21 – 7.15 (m, 2.4H), 5.03 – 4.99 (m, 0.7H), 4.98 (d, J = 3.2 Hz, 1.0H), 4.92 – 4.86 (m, 1.7H), 4.75 – 4.64 (m, 3.5H), 4.63 – 4.56 (m, 3.4H), 4.55 – 4.50 (m, 2.1H), 4.47 (s, 0.7H), 4.45 (d, J = 6.5 Hz, 0.9H), 4.05 – 3.93 (m, 2.3H), 3.92 – 3.72 (m, 4.7H), 3.53 – 3.44 (m, 1.1H); ^{13}C NMR (100 MHz, $CDCl_3$) δ 138.5, 138.4, 138.3, 138.1, 137.4, 137.3, 128.4, 128.3, 128.1, 128.0, 127.8, 127.7, 127.6, 127.5, 100.4, 97.2, 82.3, 80.2, 76.0, 75.2, 75.1, 75.0, 74.9, 74.8, 74.1, 73.5, 73.4, 72.6, 72.2, 72.1, 71.5, 70.9, 69.6, 69.3, 69.0. HRMS (ESI-TOF): calculated for $C_{41}H_{46}NO_6$ $[M+NH_4]^+$ 648.3325 found 648.3326.

2-Bromoethyl 2,3,4,6-tetra-*O*-benzyl-α/β-D-mannopyranoside (15): The product was isolated from the reaction between glycosyl donor **7α** (103 mg, 0.15 mmol, 1 equiv) and 2-bromoethanol (**2g**, 12 µl, 0.165 mmol, 1.1 equiv) following the general procedure 2(a) and was obtained as semi solid (yield: 76 mg, 78%, α/β 3:1). R_f: 0.3 (acetone/hexane 1:12 (v/v),$[\alpha]_D^{29}$ = -9.23 (c = 0.1, CH_2Cl_2); ^1H NMR (400 MHz, $CDCl_3$) δ 7.46 (dd, J = 7.4, 2.0 Hz, 0.8H), 7.41 – 7.22 (m, 26.3H), 7.20 – 7.13 (m, 2.9H), 4.99 (d, J = 12.3 Hz, 0.3H), 4.92 (d, J = 1.7 Hz, 1.0H), 4.90 – 4.85 (m, 1.8H), 4.73 (q, J = 12.4 Hz, 2.2H), 4.68 – 4.61 (m, 3.2H), 4.59 – 4.47 (m, 3.9H), 4.46 (d, J = 1.8 Hz, 0.3H), 4.43 (s, 0.1H), 4.23 (quint, J = 5.6 Hz, 0.4H), 4.03 – 3.90 (m, 3.8H), 3.89 – 3.81 (m, 2.5H), 3.80 – 3.70 (m, 4.5H), 3.55 – 3.49 (m, 1.0H), 3.48 – 3.39 (m, 2.7H); ^{13}C NMR (100 MHz, $CDCl_3$) δ 138.8, 138.7, 138.5, 138.3, 129.0, 128.7, 128.6, 128.5, 128.4, 128.3, 128.2, 128.1, 128.0, 127.9, 102.2, 98.7, 82.1, 80.3, 76.2, 75.5, 75.4, 75.1, 75.0, 74.6, 74.0, 73.9, 73.7, 73.2,

72.6, 72.5, 71.8, 70.0, 69.5, 68.0, 30.9, 30.7. HRMS (ESI-TOF): calculated for $C_{36}H_{43}BrNO_6$ $[M+NH_4]^+$ 664.2274 found 664.2264.

3-Chloropropyl 2,3,4,6-tetra-*O*-benzyl-α/β-D-mannopyranoside (16): The product was isolated from the reaction between glycosyl donor **7α** (103 mg, 0.15 mmol, 1 equiv) and 3-chloropropanol (**2h**, 14 µl, 0.165 mmol, 1.1 equiv) following the general procedure 2(a) and was obtained as semi solid (yield: 71 mg, 77%, α/β 1.9:1). R_f: 0.3 (acetone/hexane 1:12 (v/v), $[\alpha]_D^{29}$ = -8.40 (c = 0.1, CH_2Cl_2); ^1H NMR (400 MHz, $CDCl_3$) δ 7.43 (dd, J = 7.5, 2.0 Hz, 1.1H), 7.40 – 7.24 (m, 24.7H), 7.22 – 7.15 (m, 2.7H), 4.96 (s, 0.2H), 4.91 (d, J = 14.6 Hz, 0.9H), 4.87 (d, J = 2.4 Hz, 1.0H), 4.86 (s, 0.5H), 4.83 (s, 0.1H), 4.77 (s, 0.1H), 4.73 (d, J = 6.7 Hz, 1.2H), 4.69 – 4.63 (m, 2.5H), 4.62 – 4.48 (m, 4.3H), 4.41 (s, 0.3H), 4.09 – 3.97 (m, 1.4H), 3.94 – 3.78 (m, 4.4H), 3.76 (d, J = 1.6 Hz, 0.5H), 3.75 – 3.72 (m, 1.6H), 3.68 – 3.59 (m, 1.8H), 3.58 – 3.44 (m, 3.6H), 2.15 – 1.93 (m, 2.9H); ^{13}C NMR (100 MHz, $CDCl_3$) δ 138.8, 138.7, 138.6, 138.5, 138.4, 128.8, 128.7, 128.6, 128.5, 128.4, 128.2, 128.1, 128.0, 127.9, 127.8, 102.1, 98.5, 82.5, 80.4, 76.2, 75.5, 75.2, 74.5, 74.4, 73.9, 73.7, 73.1, 72.6, 72.3, 71.9, 69.7, 69.5, 66.8, 64.4, 42.1, 42.0, 33.0, 32.6. HRMS (ESI-TOF): calculated for $C_{37}H_{45}ClNO_6$ $[M+NH_4]^+$ 634.2935 found 634.2916.

Cholesteryl 2,3,4,6-tetra-*O*-benzyl-α/β-D-mannopyranoside (17) [14]**:** The product was isolated from the reaction between glycosyl donor **7α** (103 mg, 0.15 mmol, 1 equiv) and cholesterol (**2m**, 64 mg, 0.165 mmol, 1.1 equiv) following the general procedure 2(a) and was obtained as white solid (yield: 93 mg, 68%, α/β 1:1.9). R_f: 0.5 (acetone/hexane 1:10 (v/v),$[\alpha]_D^{29}$= -24.00 (c = 0.1, CH_2Cl_2); ^1H NMR (400 MHz, $CDCl_3$) δ 7.52 – 7.47 (m, 3.0H), 7.42 – 7.24 (m, 46.0H), 7.23 – 7.15 (m, 5.2H), 5.34 (d, J = 5.0 Hz, 1.2H), 5.28 (d, J = 5.0 Hz, 1.0H), 5.04 (d, J = 1.4 Hz, 1.0H), 5.03 (s, 0.4H), 4.99 (s, 0.9H), 4.95 – 4.86 (m, 3.9H), 4.80 – 4.68 (m, 2.8H), 4.67 – 4.56 (m, 6.8H), 4.54 (d, J = 3.2 Hz, 1.8H), 4.51 (d, J = 2.6 Hz, 3.3H), 4.46 (s, 0.9H), 4.43 (s,

0.4H), 4.04 – 3.92 (m, 2.3H), 3.91 – 3.78 (m, 6.6H), 3.77 – 3.70 (m, 3.8H), 3.63 – 3.54 (m, 1.6H), 3.53 – 3.42 (m, 3.9H), 2.37 – 2.20 (m, 5.0H), 2.12 – 1.90 (m, 6.7H), 1.89 – 1.74 (m, 6.4H), 1.72 – 1.44 (m, 17.6H), 1.43 – 1.23 (m, 15.0H), 1.22 – 0.82 (m, 59.6H), 0.69 (d, *J* = 5.7 Hz, 7.7H); ^{13}C NMR (100 MHz, $CDCl_3$) *δ* 140.7, 140.6, 138.9, 138.7, 138.6, 138.5, 138.4, 138.2, 128.5, 128.3, 128.1, 128.0, 127.8, 127.6, 127.5, 127.4, 127.3, 121.9, 99.7, 95.7, 82.5, 80.4, 78.7, 76.5, 75.9, 75.2, 75.1, 75.0, 74.1, 73.8, 73.4, 73.3, 72.5, 72.1, 71.7, 71.4, 69.9, 69.4, 56.8, 56.2, 50.2, 50.1, 42.3, 39.9, 39.8, 39.5, 38.9, 37.3, 37.0, 36.8, 36.7, 36.2, 35.8, 32.0, 31.9, 29.8, 28.3, 28.0, 27.6, 24.3, 23.8, 22.8, 22.6, 21.1, 19.5, 19.4, 18.7, 11.9.

6-*O*-(2,3,4,6-Tetra-*O*-benzyl-α/β-D-mannopyranosyl)-1,2;3,4-di-*O*-isopropylidene-α-D-galactopyranose (18) [10]**:** The product was isolated from the reaction between glycosyl donor **7α** (103 mg, 0.15 mmol, 1 equiv) and glycosyl acceptor **2n** (43 mg, 0.165 mmol, 1.1 equiv) following the general procedure 2(a) and was obtained as semi solid (yield: 72 mg, 61%, *α* only). R_f: 0.4 (acetone/hexane 1:5 (v/v), $[\alpha]_D^{29}$ = -28.30 (c = 0.1, CH_2Cl_2); ^{1}H NMR (400 MHz, $CDCl_3$) *δ* 7.40 – 7.17 (m, 23.9H), 7.13 – 7.05 (m, 2.6H), 5.45 (d, *J* = 4.9 Hz, 1.0H), 4.94 (s, 1.1H), 4.79 (d, *J* = 10.6 Hz, 1.4H), 4.73 – 4.58 (m, 3.9H), 4.57 – 4.39 (m, 6.5H), 4.28 – 4.21 (m, 1.4H), 4.08 (d, *J* = 7.9 Hz, 1.2H), 3.99 – 3.80 (m, 4.3H), 3.78 – 3.55 (m, 7.2 H), 1.43 (s, 3.8H), 1.36 (s, 3.6H), 1.26 (s, 7.5H), 1.18 (s, 2.9H); ^{13}C NMR (100 MHz, $CDCl_3$) *δ* 138.5, 138.4, 138.2, 128.4, 128.1, 127.9, 127.7, 127.6, 109.4, 108.6, 97.3, 96.3, 80.0, 75.1, 74.7, 74.5, 73.3, 72.4, 72.1, 71.9, 70.9, 70.7, 70.6, 68.9, 65.5, 65.3, 26.1, 26.0, 24.9, 24.6.

1-Adamantanemethyl 2,3-di-*O*-benzyl-4,6-*O*-benzylidine-α-D-glucopyranoside (19): The product was isolated from the reaction between glycosyl donor **8α** (89 mg, 0.15 mmol, 1 equiv) and 1-adamantanemethanol (**2i**, 27 mg, 0.165 mmol, 1.1 equiv) following the general procedure 2(a) and was obtained as sticky liquid (yield: 55 mg, 61%, α only). R_f: 0.4 (acetone/hexane 1:10

(v/v), $[\alpha]_D^{29}$ = +30.67 (c = 0.1, CH_2Cl_2); ^{1}H NMR (400 MHz, $CDCl_3$) δ 7.55 (dd, *J* = 7.6, 1.7 Hz, 2.6H), 7.49 – 7.25 (m, 16.4H), 5.60 (s, 1.0H), 5.01 – 4.80 (m, 3.5H), 4.75 (d, *J* = 3.6 Hz, 1.0H), 4.69 (d, *J* = 11.9 Hz, 1.1H), 4.31 (dd, *J* = 10.0, 4.7 Hz, 1.0H), 4.09 (t, *J* = 9.2 Hz, 1.0H), 3.86 (td, *J* = 10.0, 4.7 Hz, 1.1H), 3.74 (t, *J* = 10.2 Hz, 1.2H), 3.68 – 3.58 (m, 2.1H), 3.32 (d, *J* = 9.1 Hz, 1.0H), 2.93 (d, *J* = 9.2 Hz, 1.0H), 2.02 (s, 3.4H), 1.83 – 1.55 (m, 15.2H); ^{13}C NMR (100 MHz, $CDCl_3$) δ 139.0, 138.6, 137.5, 128.9, 128.4, 128.3, 128.2, 127.9, 127.7, 127.5, 126.0, 101.1, 98.4, 82.4, 80.0, 79.2, 78.5, 75.2, 73.1, 69.2, 62.3, 39.6, 37.1, 33.9, 28.2. HRMS (ESI-TOF): calculated for $C_{38}H_{45}O_6$ $[M+H]^+$ 597.3216 found 597.3208.

Cholesteryl 2,3-di-*O*-benzyl-4,6-*O*-benzylidine-α-D-glucopyranoside (20): The product was isolated from the reaction between glycosyl donor **8α** (89 mg, 0.15 mmol, 1 equiv) and cholesterol **2m** (64 mg, 0.165 mmol, 1.1 equiv) following the general procedure 2(a) and was obtained as white solid (yield: 67 mg, 54%, α only). R_f: 0.5 (acetone/hexane 1:10 (v/v), $[\alpha]_D^{29}$ = +27.35 (c = 0.1, CH_2Cl_2); ^{1}H NMR (400 MHz, $CDCl_3$) δ 7.50 (dd, *J* = 7.6, 1.9 Hz, 2.2H), 7.43 – 7.21 (m, 15.6H), 5.56 (s, 1.0H), 5.37 – 5.31 (m, 1.0H), 4.95 – 4.89 (m, 2.1H), 4.87 – 4.80 (m, 2.2H), 4.69 (d, *J* = 12.1 Hz, 1.0H), 4.27 (dd, *J* = 10.2, 4.9 Hz, 1.0H), 4.07 (t, *J* = 9.3 Hz, 1.1H), 3.96 (td, *J* = 10.0, 4.8 Hz, 1.1H), 3.69 (t, *J* = 10.3 Hz, 1.1H), 3.61 (t, *J* = 9.4 Hz, 1.1H), 3.55 (dd, *J* = 9.3, 3.8 Hz, 1.0H), 3.51 – 3.39 (m, 1.3H), 2.53 – 2.42 (m, 1.1H), 2.32 – 2.25 (m, 1.1H), 2.05 – 1.78 (m, 5.7H), 1.67 – 1.05 (m, 24.4H), 1.04 – 0.96 (m, 6.2H), 0.92 (d, *J* = 6.5 Hz, 4.1H), 0.87 (dd, *J* = 6.6, 1.7 Hz, 7.4H), 0.68 (s, 3.4H); ^{13}C NMR (100 MHz, $CDCl_3$) δ 140.7, 138.3, 137.5, 128.9, 128.4, 128.3, 128.2, 128.0, 127.8, 127.5, 126.0, 121.9, 101.1, 96.0, 82.4, 79.3, 78.7, 78.5, 75.3, 73.4, 62.5, 56.8, 50.2, 46.1, 42.3, 40.0, 39.8, 39.5, 37.1, 36.8, 36.2, 35.8, 32.2, 31.9, 28.2, 28.0, 27.6, 24.3, 23.8, 22.8, 22.6, 21.1, 19.4, 18.7, 11.9. HRMS (ESI-TOF): calculated for $C_{54}H_{76}NO_6$ $[M+NH_4]^+$ 834.5673 found 834.5660.

6-*O*-(2,3-Di-*O*-benzyl-4,6-*O*-benzylidine-α-D-glucopyranoside)-1,2;3,4-di-*O*-isopropylidene-α-D-galactopyranose (21) [14]**:** The product was isolated from the reaction between glycosyl donor **8α** (89 mg, 0.15 mmol, 1 equiv) and glycosyl acceptor **2n** (43 mg, 0.165 mmol, 1.1 equiv) following the general procedure 2(a) and was obtained as semi solid (yield: 63 mg, 61%, α only). R_f: 0.4 (acetone/hexane 1:5 (v/v), $[\alpha]_D^{29}$= +29.51 (c = 0.1, CH_2Cl_2); ^{1}H NMR (400 MHz, $CDCl_3$) δ 7.53 – 7.47 (m, 2.0H), 7.42 – 7.24 (m, 13.9H), 5.56 (s, 1.1H), 5.54 (s, 0.4H), 4.93 (d, *J* = 3.5 Hz, 1.0H), 4.90 (s, 0.5H), 4.86 – 4.79 (m, 1.2H), 4.77 (d, *J* = 4.8 Hz, 1.4H), 4.61 (dd, *J* = 7.9, 2.4 Hz, 1.0H), 4.37 – 4.26 (m, 2.7H), 4.05 (t, *J* = 9.3 Hz, 1.9H), 3.95– 3.87 (m, 1.0H), 3.83 – 3.56 (m, 4.9H), 1.55 (s, 2.4H), 1.46 (s, 2.7H), 1.33 (d, *J* = 4.7 Hz, 5.7H); ^{13}C NMR (100 MHz, $CDCl_3$) δ 139.1, 138.5, 129.2, 128.7, 128.6, 128.5, 128.3, 128.1, 127.9, 126.4, 109.6, 109.0, 101.5, 98.5, 96.6, 82.4, 79.5, 78.8, 75.6, 73.2, 71.2, 70.9, 69.3, 67.2, 66.2, 62.8, 26.5, 26.4, 25.2, 24.9.

Methyl 2,3,4,6-tetra-*O*-benzyl-α/β-D-glucopyranosyl-(1→6)-2,3-di-O-benzyl-α-D-glucopyranoside (23) [15]**:** The product was isolated from the reaction between glycosyl donor **1α** (103 mg, 0.15 mmol, 1 equiv) and glycosyl acceptor **22** (77 mg, 0.165 mmol, 1.1 equiv) following the general procedure 2(a) and was obtained as sticky liquid (yield: 83 mg, 62%, α/β 3.2:1)**.** R_f: 0.35 (acetone/hexane 1:4 (v/v), $[\alpha]_D^{29}$= +47.15 (c = 0.4, CH_2Cl_2); ^{1}H NMR (400 MHz, $CDCl_3$) δ 7.33 – 7.12 (m, 38.3H), 7.10 – 7.01 (m, 2.9H), 4.95 – 4.80 (m, 3.2H), 4.77 – 4.62 (m, 7.5H), 4.61 – 4.41 (m, 6.5H), 4.39 (d, *J* = 4.8 Hz, 1.1H), 4.36 (d, *J* = 3.0 Hz, 0.7H), 4.05 (d, *J* = 10.3 Hz, 0.4H), 3.93 – 3.80 (m, 2.4H), 3.76 – 3.62 (m, 4.9H), 3.61 – 3.44 (m, 7.8H), 3.41 (dd, *J* = 9.5, 3.2 Hz, 1.9H), 3.29 (s, 3H), 3.26 (s, 0.9H); ^{13}C NMR (100 MHz, $CDCl_3$) δ 139.3, 139.1, 138.5, 138.3, 138.1, 128.8, 128.7, 128.3, 128.2, 128.0, 127.9, 104.0, 98.4, 97.8, 85.0, 82.3, 81.8,

81.7, 80.1, 79.9, 79.8, 78.0, 77.9, 76.0, 75.9, 75.7, 75.3, 75.1, 73.7, 73.6, 73.5, 73.4, 72.1, 70.6, 70.4, 69.8, 69.1, 69.0, 68.7, 55.6.

Methyl 2,3,4,6-tetra-O-benzyl-α/β-D-mannopyranosyl-(1→6)-2,3-di-O-benzyl-α-D-glucopyranoside (24): The product was isolated from the reaction between glycosyl donor **7*α*** (103 mg, 0.15 mmol, 1 equiv) and glycosyl acceptor **22** (77 mg, 0.165 mmol, 1.1 equiv) following the general procedure 2(a) and was obtained as sticky liquid (yield: 85 mg, 63%, α only). R_f: 0.35 (acetone/hexane 1:4 (v/v), $[\alpha]_D^{29}$ = -14.71 (c = 0.2, CH_2Cl_2); ^{1}H NMR (400 MHz, $CDCl_3$) δ 7.46 – 7.20 (m, 30.8H), 7.16 (bs, 2.6H), 5.00 (d, J = 11.3 Hz, 1.1H), 4.93 (s, 1.0H), 4.90 – 4.54 (m, 12.4H), 4.50 (d, J = 10.8 Hz, 1.1H), 4.05 (d, J = 9.9 Hz, 1.0H), 3.98 – 3.77 (m, 5.8H), 3.76 – 3.56 (m, 5.9H), 3.55 – 3.49 (m, 1.2H), 3.41 (s, 0.3H), 3.36 (s, 3.0H); ^{13}C NMR (100 MHz, $CDCl_3$) δ 138.8, 138.5, 138.3, 138.2, 138.1, 138.0, 128.5, 128.4, 128.3, 128.1, 128.0, 127.9, 127.8, 127.6, 98.3, 98.1, 81.6, 80.2, 79.6, 75.7, 75.2, 75.0, 74.8, 73.3, 73.2, 72.7, 72.2, 72.0, 70.1, 69.4, 69.1, 65.6, 55.2. HRMS (ESI-TOF): calculated for $C_{55}H_{60}NaO_{11}$ $[M+Na]^+$ 919.4033 found 919.4036.

Controlled experiments:

Table S1: Concentration dependent controlled experiments[(a)]

Entry	Acceptor **2a** loading	Catalyst **3a** loading	Yield[(b)]	
			**5a**	**1*α***
1	10 mol%	10 mol%	trace	97%
2	50 mol%	50 mol%	21%	68%
3	100 mol%	100 mol%	36%	56%

(a) Reaction Conditions: To a suspension of **2a** and **3a** in dry DCM stirred for 30 mins at room temperature 10 mol% of **4** and 1 equiv. of **1*α*** was added and reaction mixture was further stirred at the room temperature for additional 2h. (b) Isolated yield after column chromatography.

References:

1) Schmidt, R. R.; Michel, J. *Angew. Chem. Int. Ed.* **1980,** *19*, 731-732.
2) Schmidt, R. R.; Kinzy, W. *Adv. Carbohydr. Chem. Biochem.* **1994,** *50*, 21-123.
3) Chan, J.; Lu, A.; Bennet, A. J. *J. Am. Chem. Soc.* **2011,** *133*, 2989-2997.
4) Michigami, K.; Terauchi, M.; Hayashi, M. *Synthesis* **2013,** *45*, 1519-1523.
5) Berkessel, A.; Das, S.; Pekel, D.; Neudörfl, J.-M. *Angew. Chem. In. Ed.* **2014,** *53*, 11660-11664.
6) Kim, H. Y.; Oh, K. *Org. Lett.* **2011,** *13*, 1306-1309.
7) Geng, Y.; Kumar, A.; Faidallah, H. M.; Albar, H. A.; Mhkalid, I. A.; Schmidt, R. R. *Angew. Chem. In. Ed.* **2013,** *52*, 10089-10092.
8) Mensah, E. A.; Azzarelli, J. M.; Nguyen, H. M. *J. Org. Chem.* **2009,** *74*, 1650-1657.
9) Kumar, A.; Geng, Y.; Schmidt, R. R. *Adv. Synth.Catal.* 2012, 354, 1489-1499.
10) Medina, S.; Henderson, A. S.; Bower, J. F.; Galan, M. C. *Chem. Commun.* **2015,** *51*, 8939-8941.
11) Vibhute, A. M.; Dhaka, A.; Athiyarath, V.; Sureshan, K. M. *Chem. Sci.* **2016,** *7*, 4259-4263.
12) Koppolu, S. R.; Niddana, R.; Balamurugan, R. *Org. Biomol.Chem.* **2015,** *13*, 5094-5097.
13) Sun, L.; Wu, X.; Xiong, D.-C.; Ye, X.-S. *Angew. Chem. In. Ed.* **2016,** *55*, 8041-8044.
14) Mukherjee, M. M.; Basu, N.; Ghosh, R. *RSC Adv.* **2016,** *6*, 105589-105606.
15) Peng, P.; Schmidt, R. R. *J. Am. Chem. Soc.* **2015,** *137*, 12653-12659.

NMR spectra

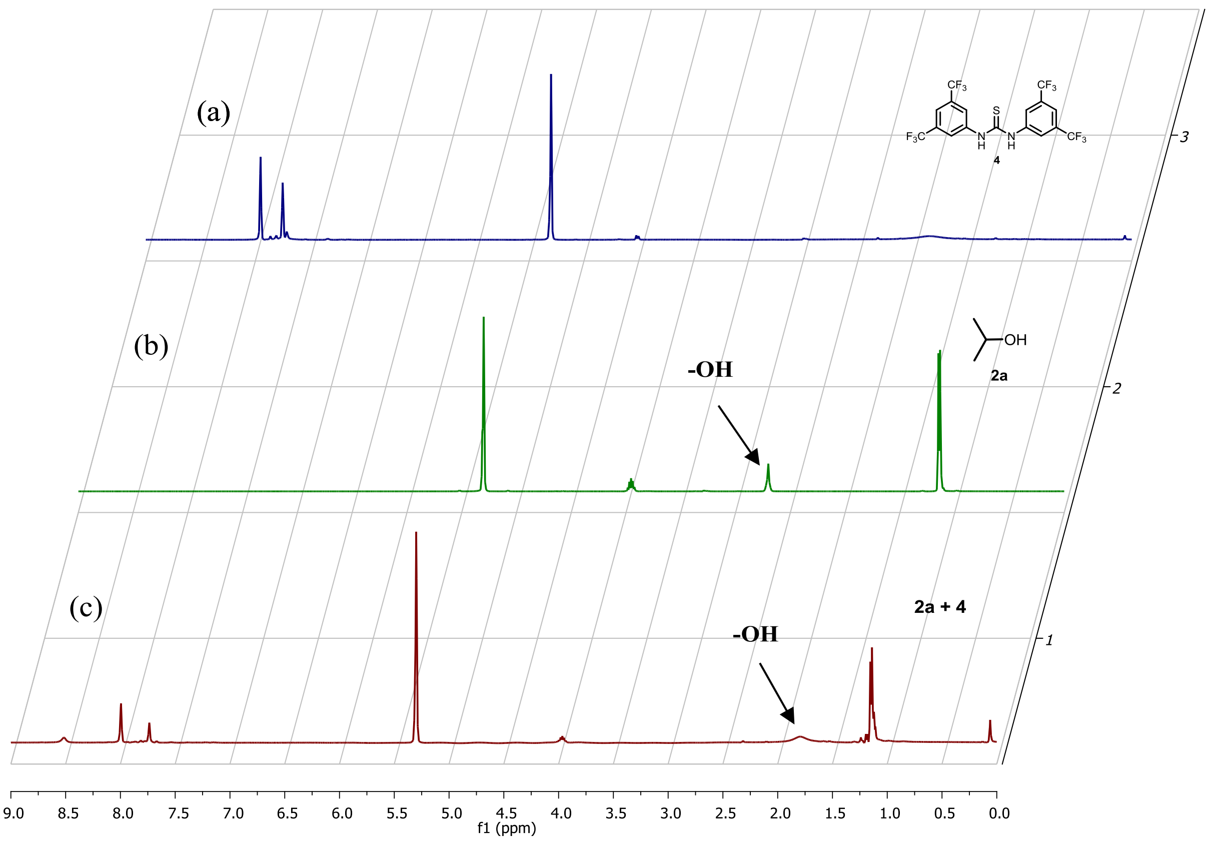

Figure S1. (a) ^{1}H NMR spectra of acceptor **4** in CD_2Cl_2; (b) ^{1}H NMR spectra of pyridinium salt **2a** in CD_2Cl_2; (c) ^{1}H NMR spectra of a mixture of **2a** and 10 mol % of **4** in CD_2Cl_2. In presence of 10 mol % of **4** the -OH peak of **2a** shifts to upfield δ 2.70 to δ 1.79 indicating thiourea **4** amplifies the nucleophilicity of glycosyl acceptor by imparting partial negative charge.

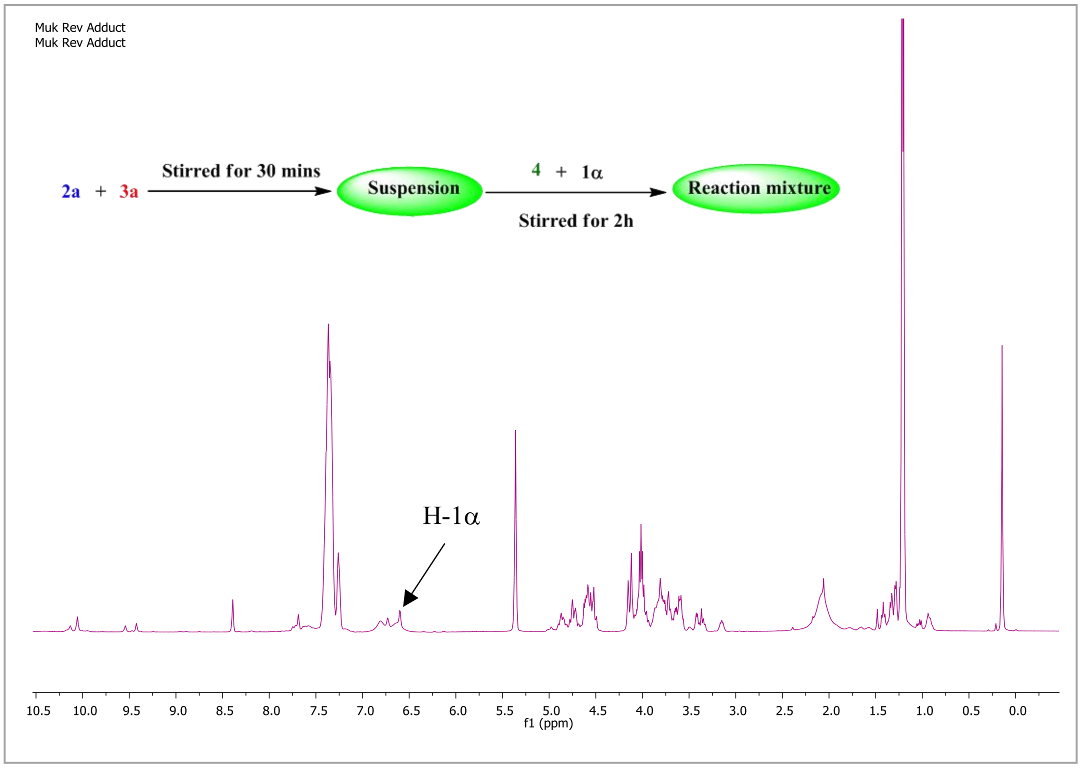

Figure S2: ^{1}H NMR of reaction mixture of 1 equiv. of both **2a** and **3a**, treated with **1α** and 10 mol % of **4** at room temperature for 2h in CD_2Cl_2. The presence of H-1α signal of **1α** indicates reaction was not completed even after 2h. If the reaction would proceed through intra molecular pathway, full conversion of **1α** would take place. The incompletion of reaction confirms the reaction would have proceeded through intermolecular pathway.

^{1}H NMR spectrum of compound **3a** (400 MHz, DMSO-d_6)

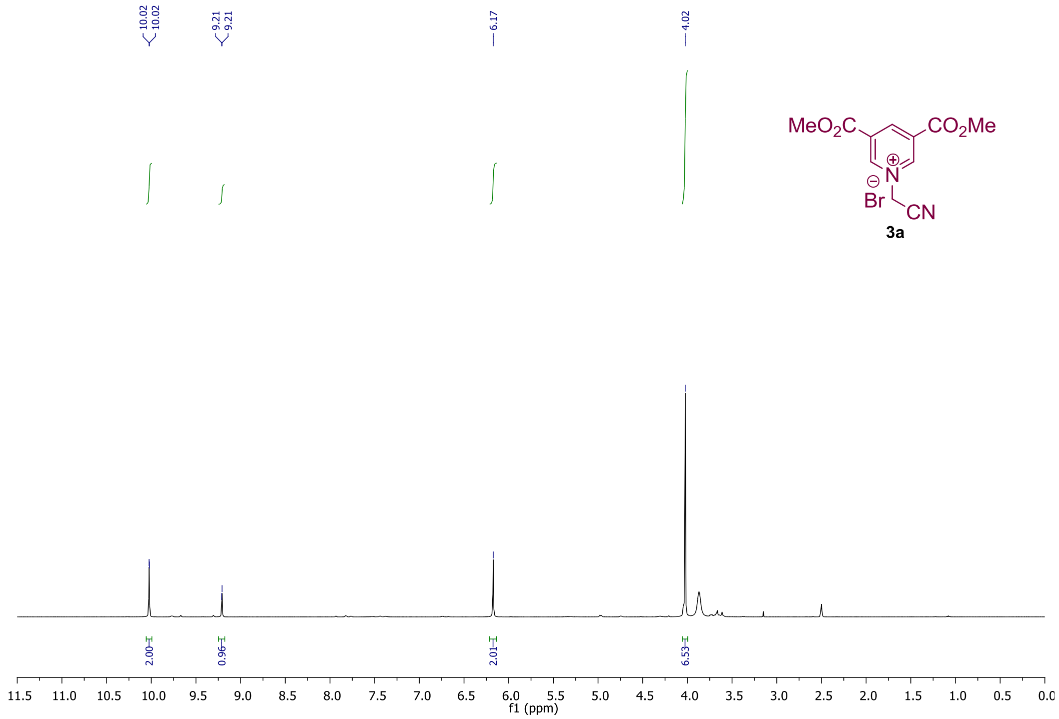

^{13}C NMR spectrum of compound **3a** (100 MHz, DMSO-d_6)

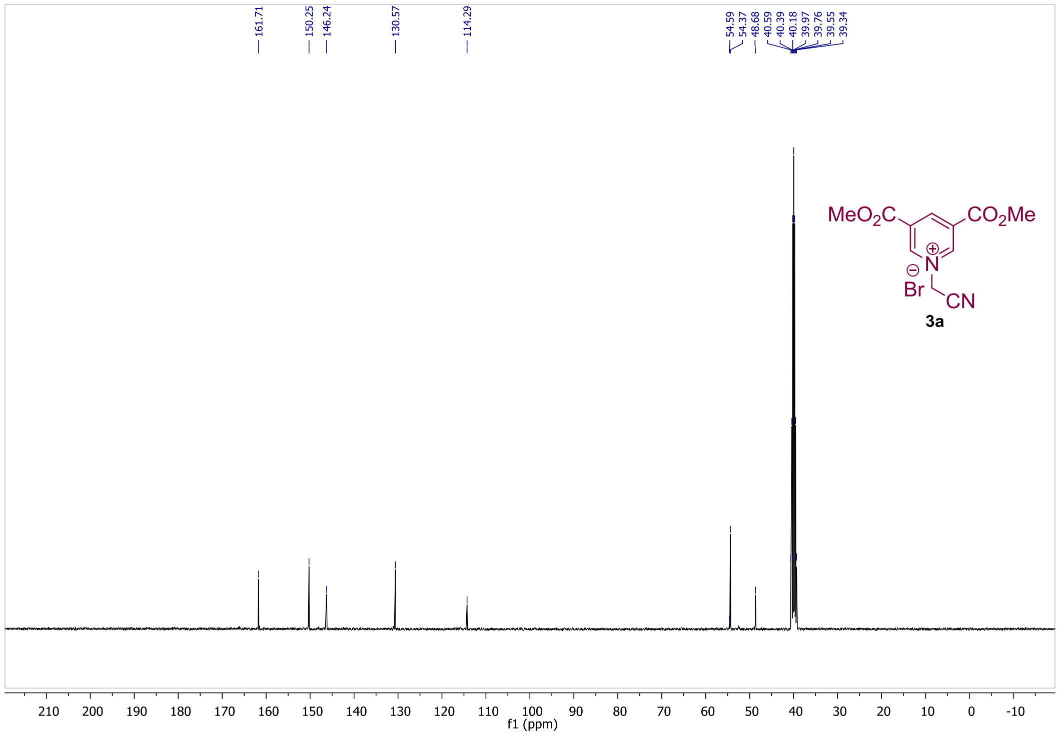

^{1}H NMR spectrum of compound **3b** (400 MHz, DMSO-d_6)

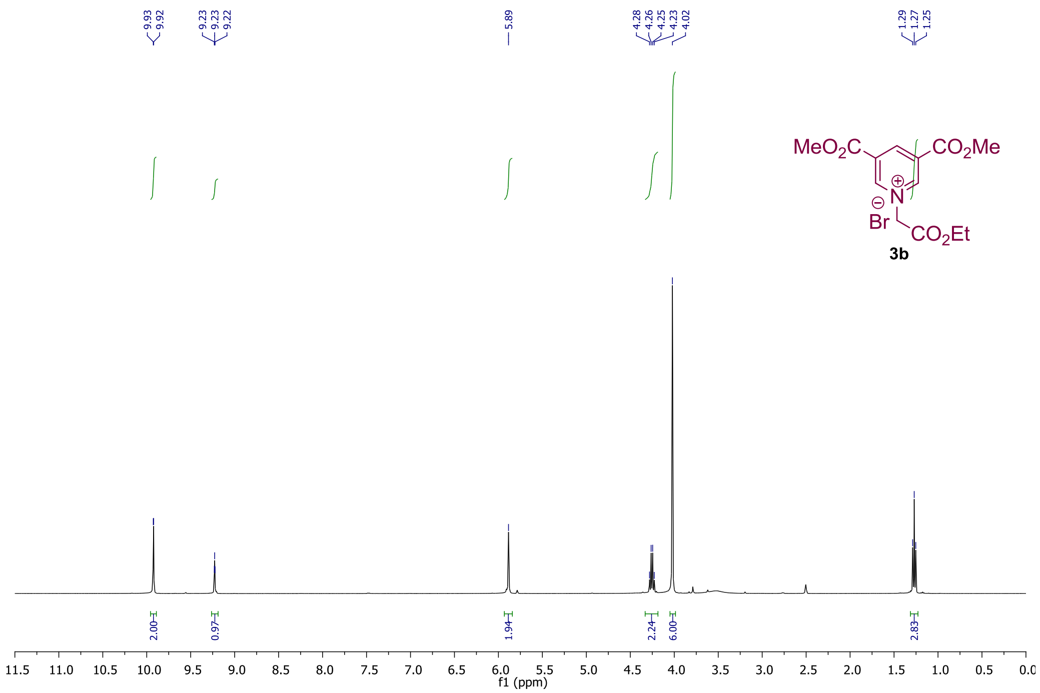

^{13}C NMR spectrum of compound **3b** (100 MHz, DMSO-d_6)

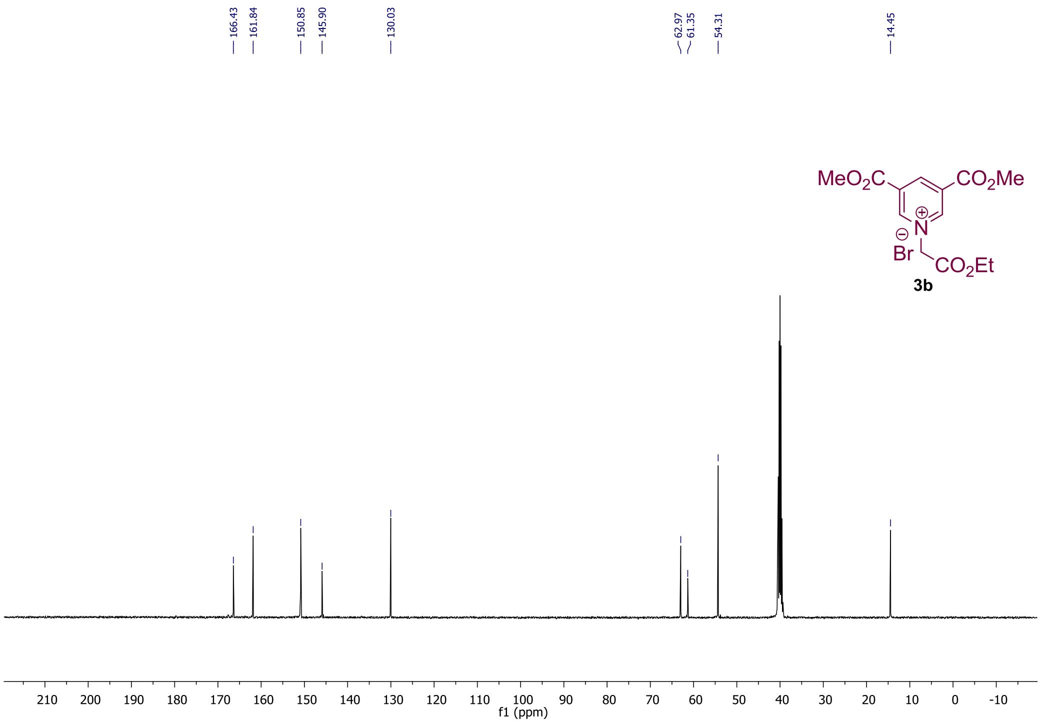

^{1}H NMR spectrum of compound **3c** (400 MHz, DMSO-d_6)

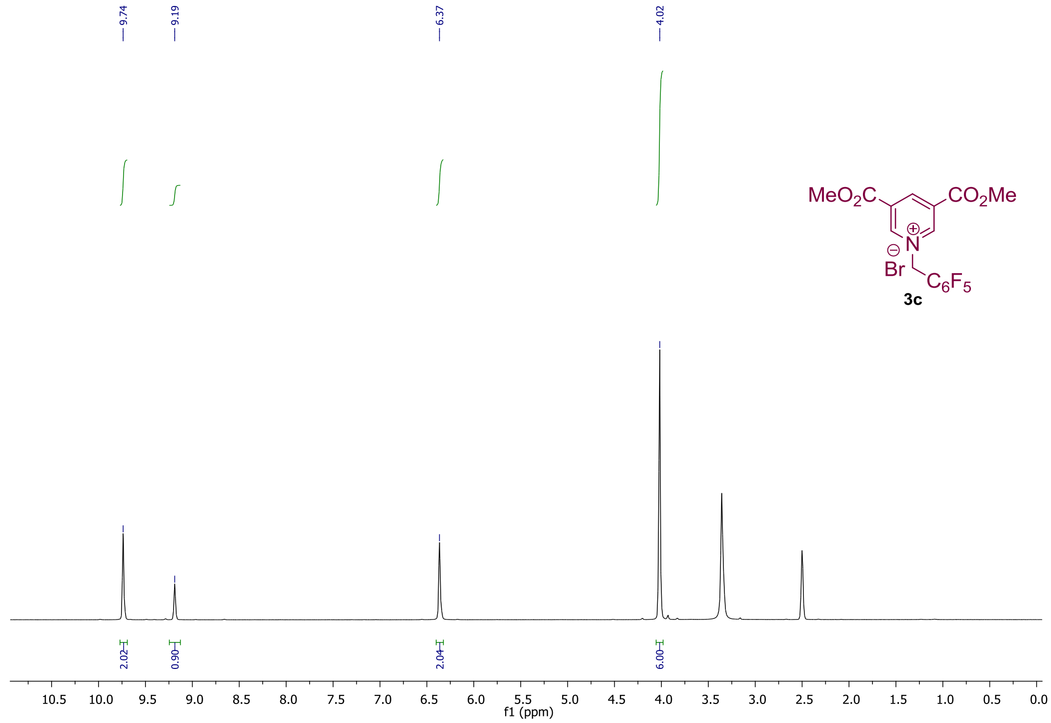

^{13}C NMR spectrum of compound **3c** (100 MHz, DMSO-d_6)

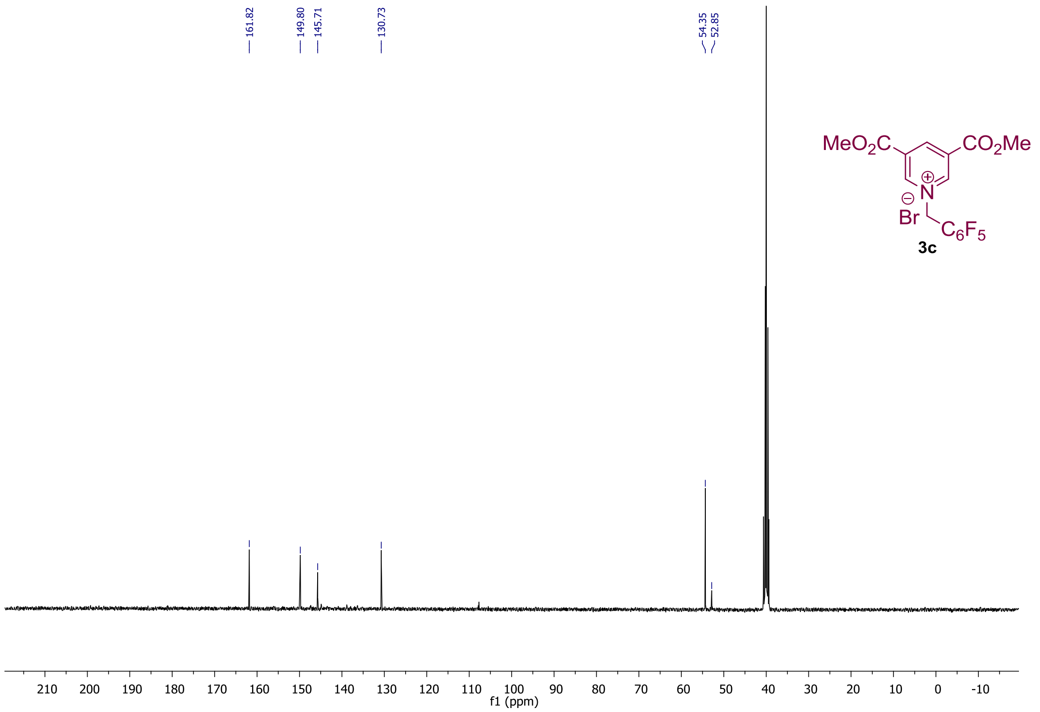

^{19}F NMR spectrum of compound **3c** (376 MHz, DMSO-d_6)

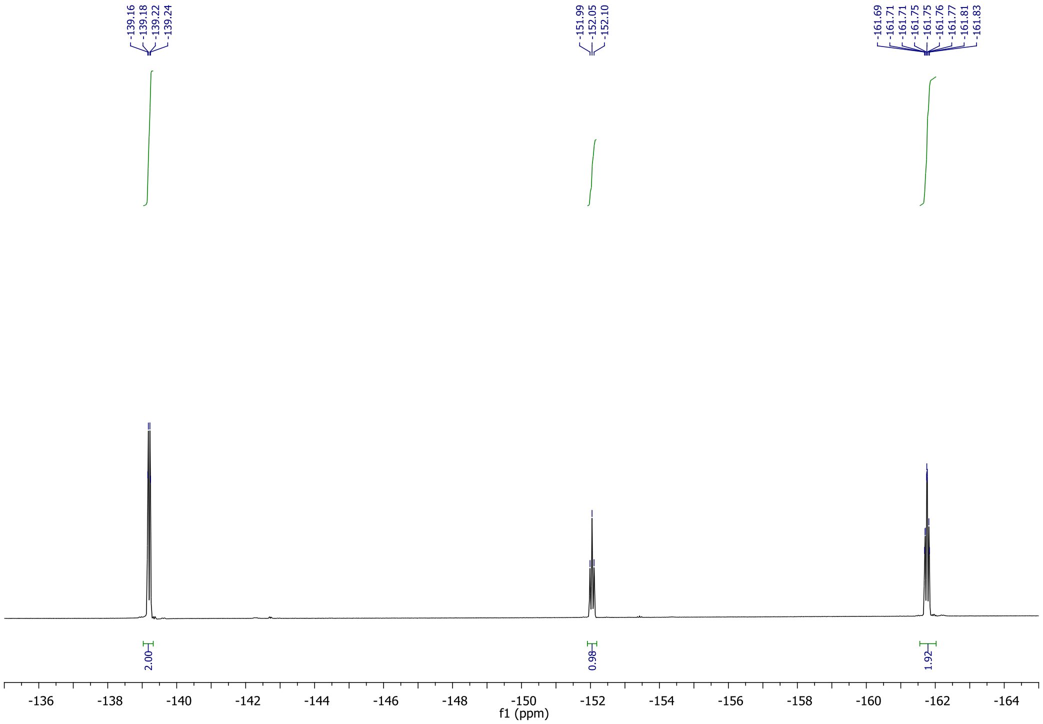

^{1}H NMR spectrum of compound **4** (400 MHz, DMSO-d_6)

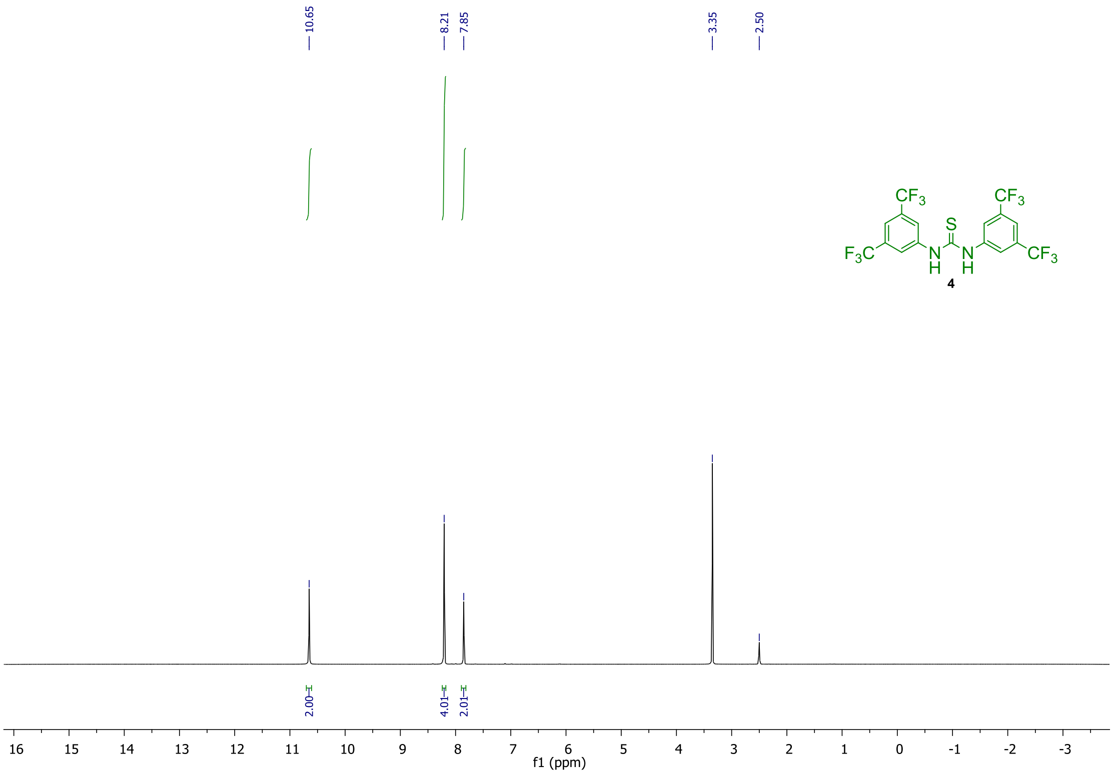

^{13}C NMR spectrum of compound **4** (100 MHz, DMSO-d_6)

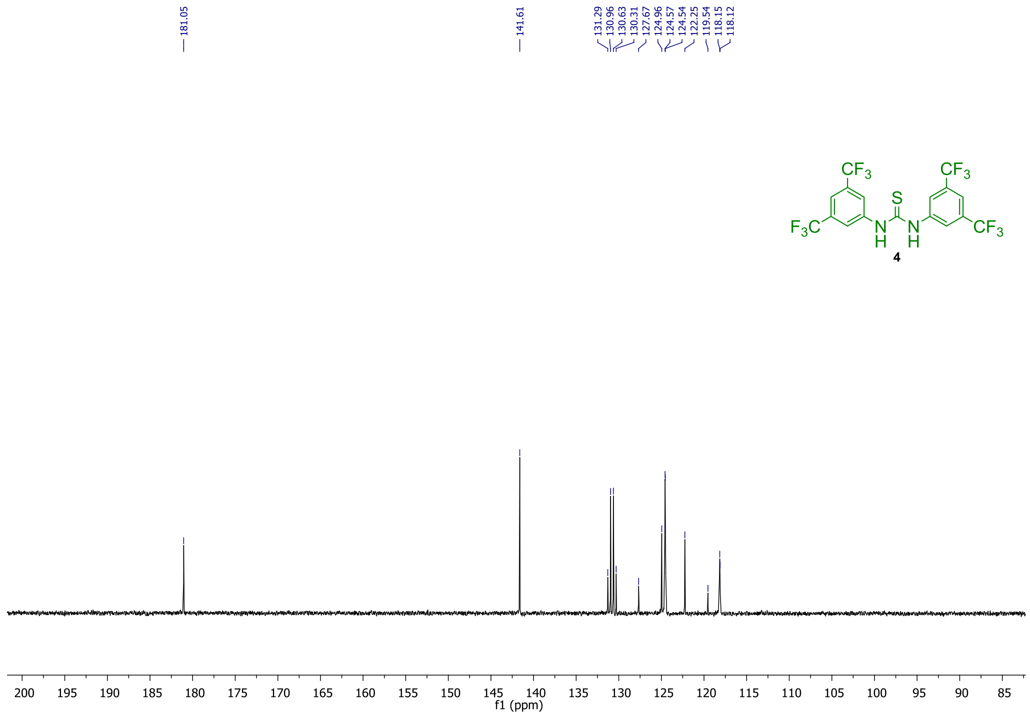

^{1}H NMR spectrum of compound **5a** (400 MHz, $CDCl_3$)

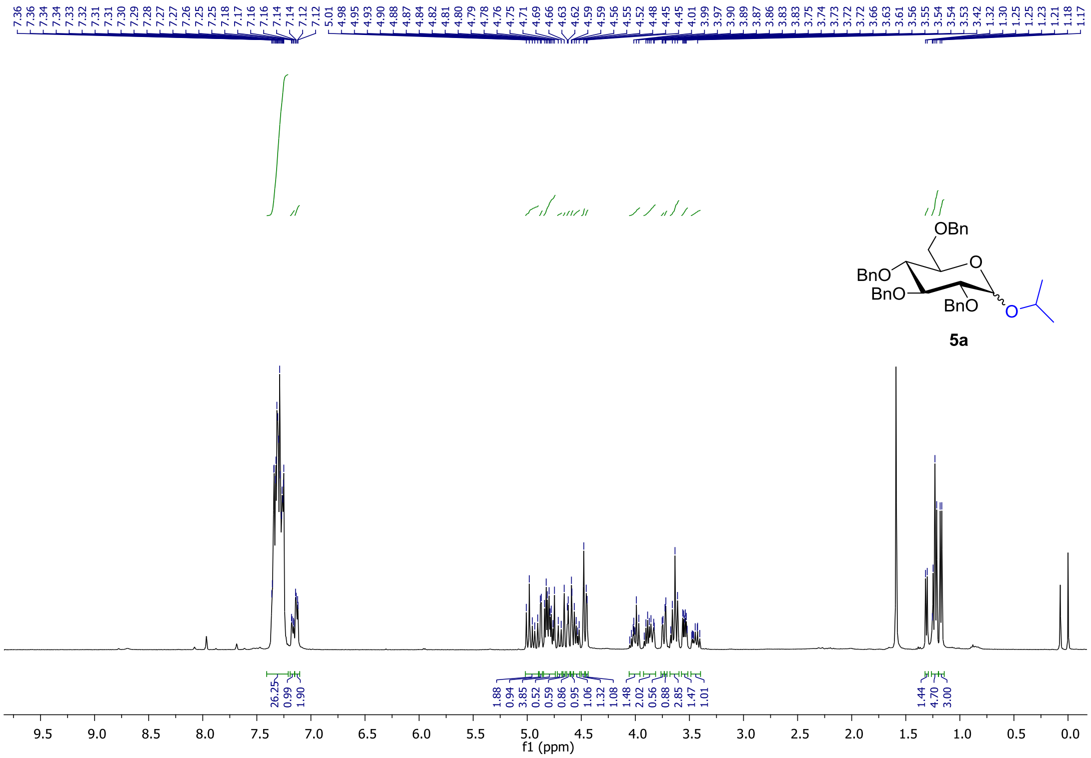

^{13}C NMR spectrum of compound **5a** (100 MHz, $CDCl_3$)

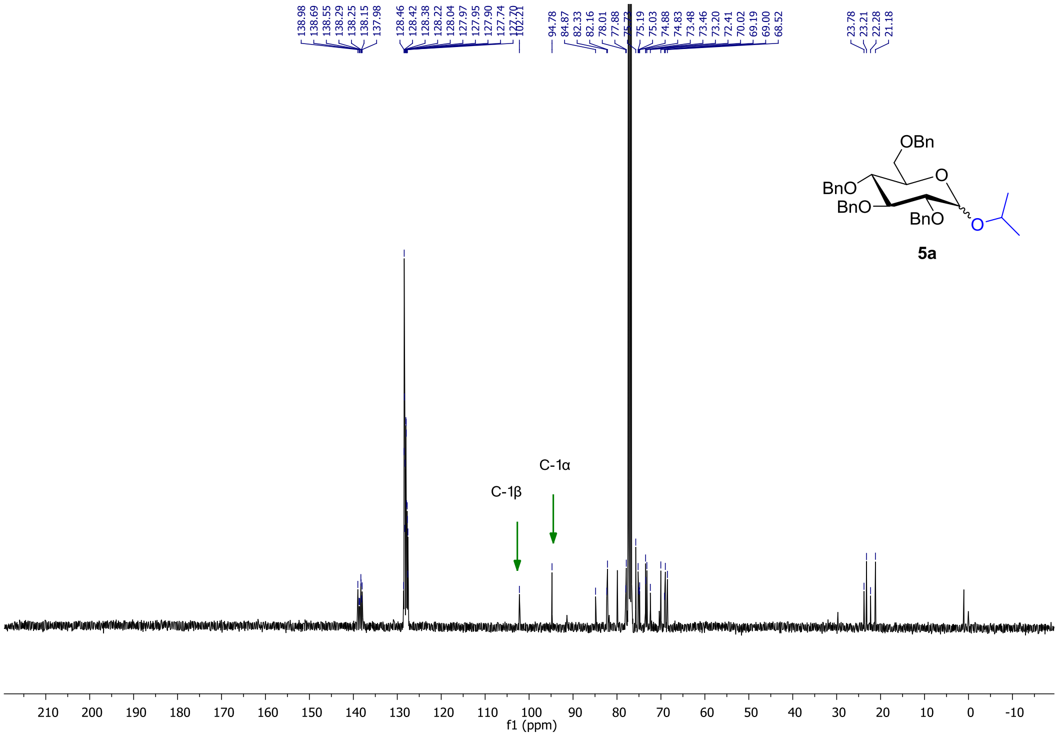

^{1}H NMR spectrum of compound **5b** (400 MHz, $CDCl_3$)

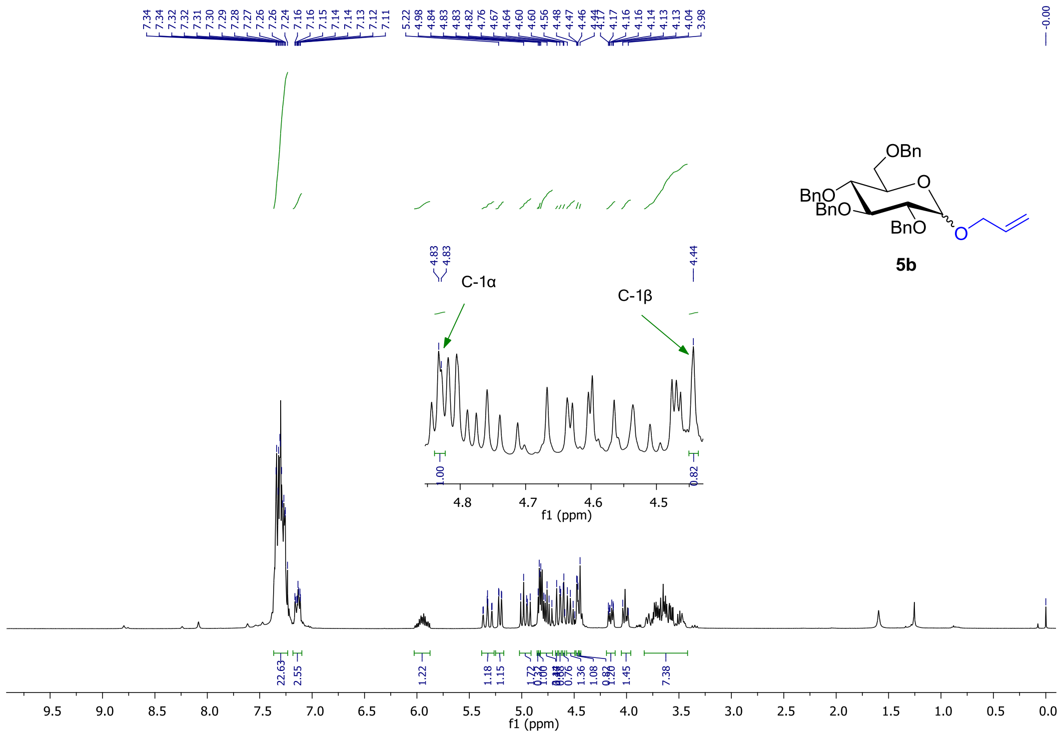

^{13}C NMR spectrum of compound **5b** (100 MHz, $CDCl_3$)

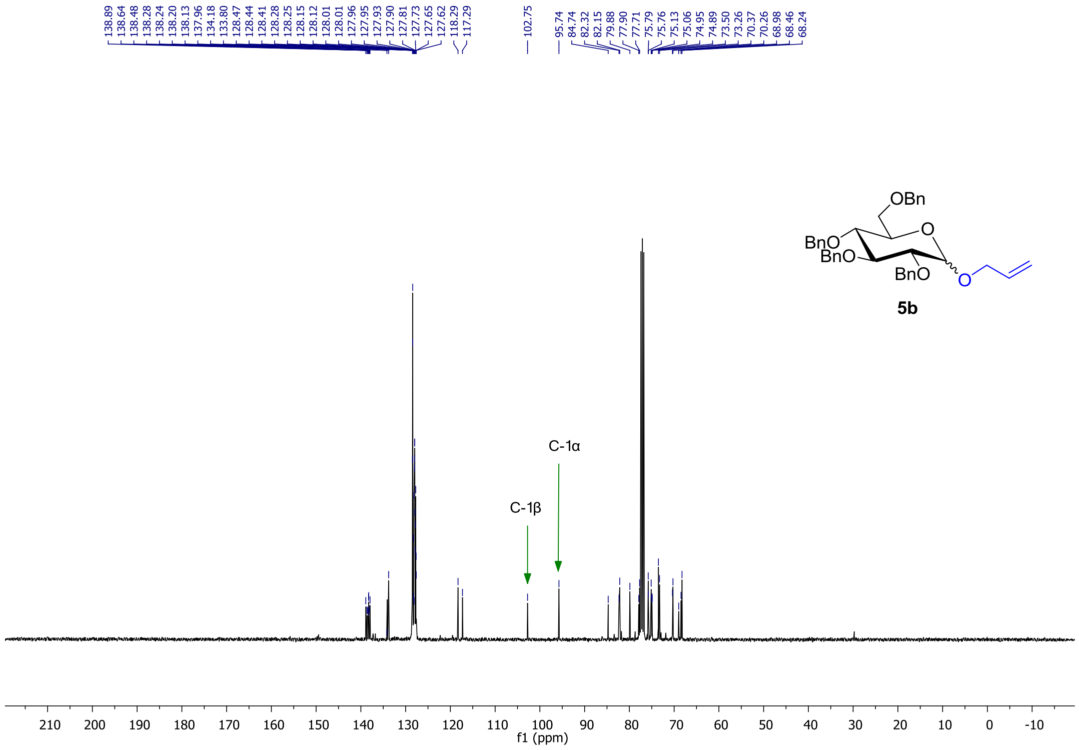

COSY spectrum of compound **5b**

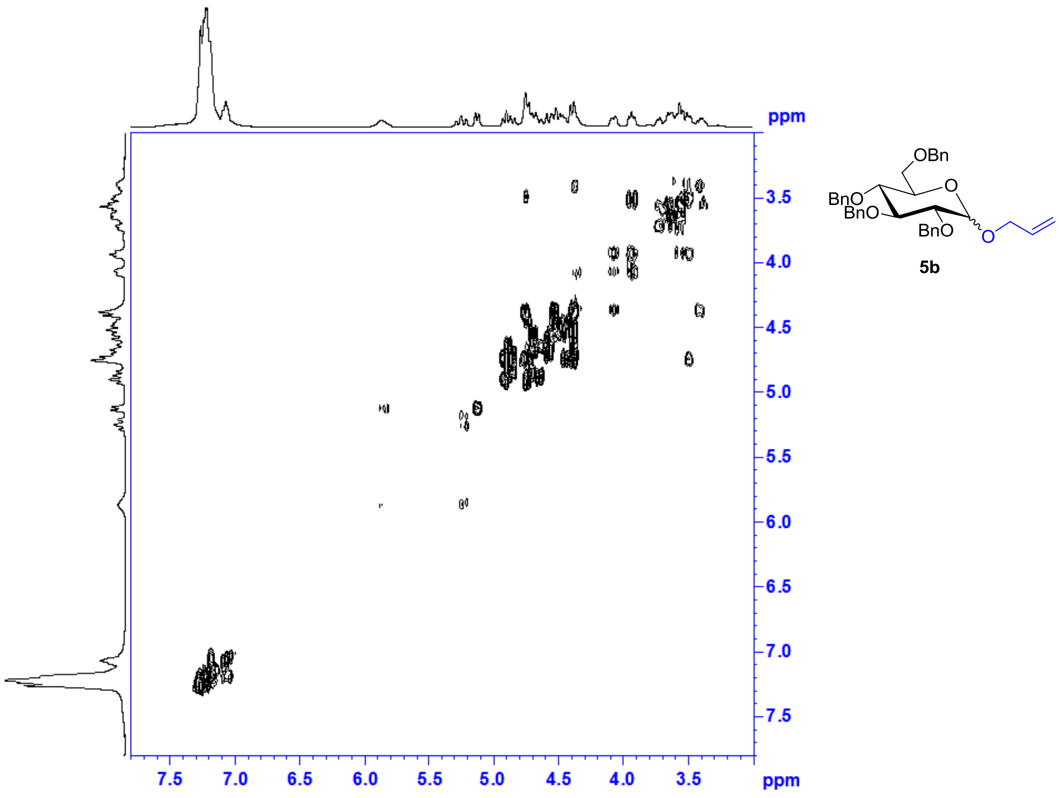

HSQC spectrum of compound **5b**

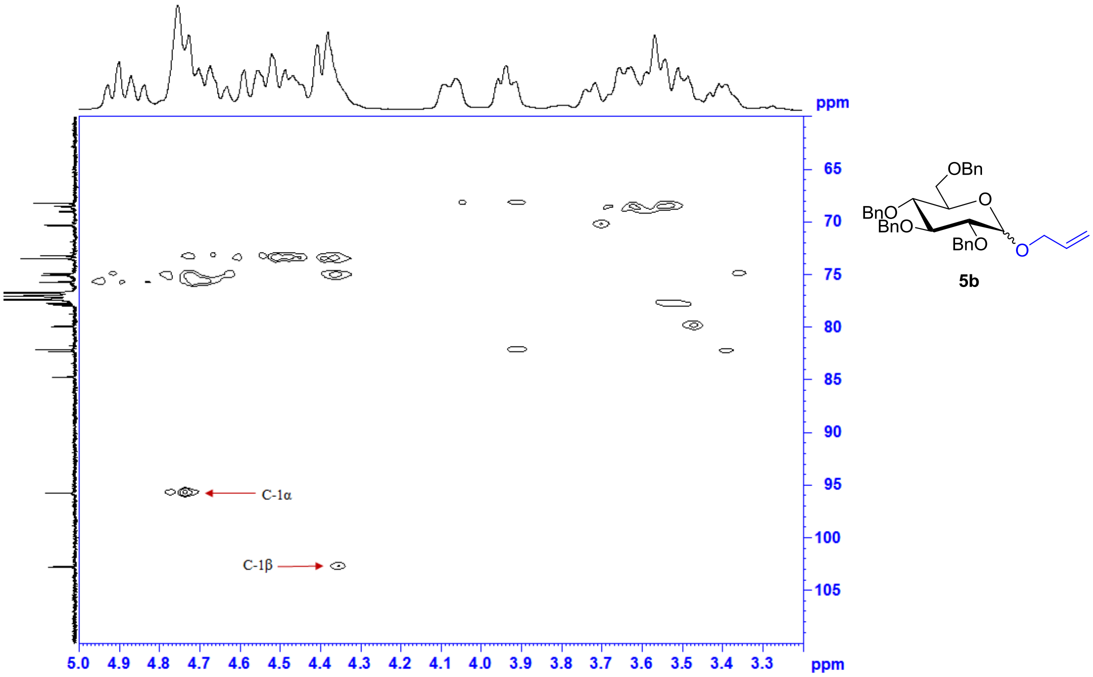

^{1}H NMR spectrum of compound **5c** (400 MHz, $CDCl_3$)

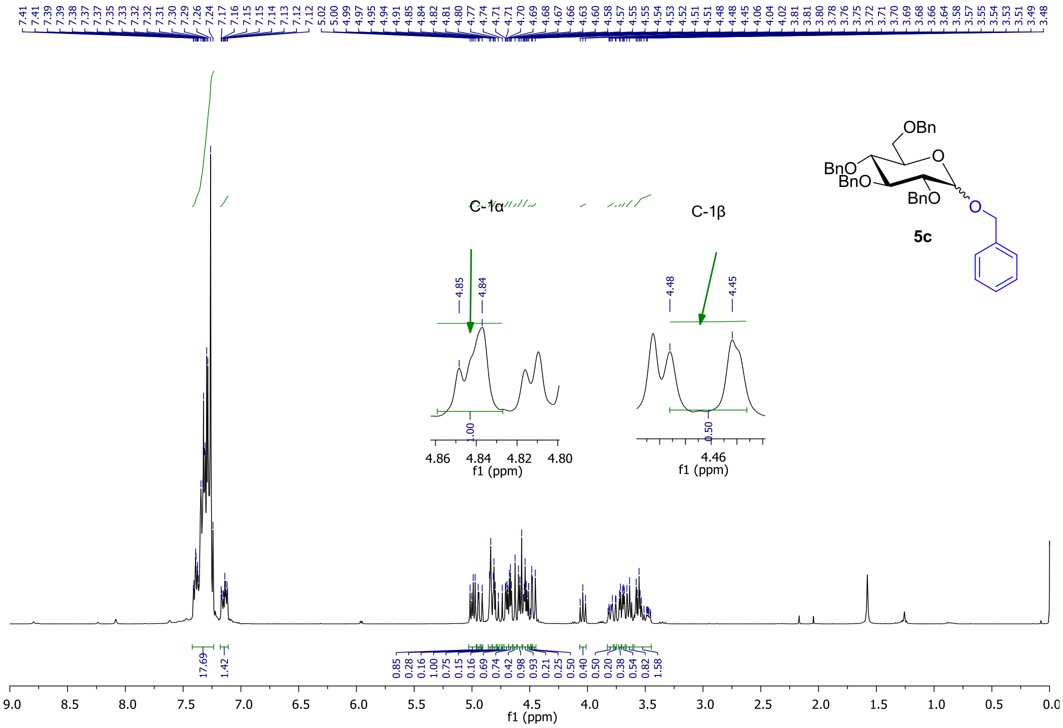

^{13}C NMR spectrum of compound **5c** (100 MHz, $CDCl_3$)

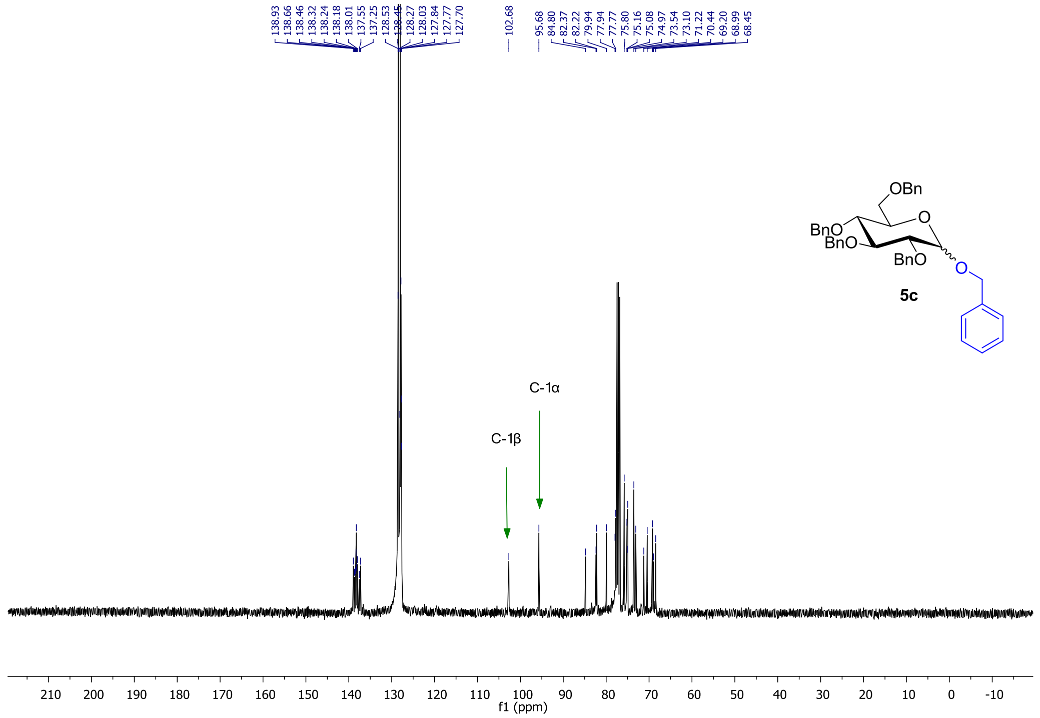

COSY spectrum of compound **5c**

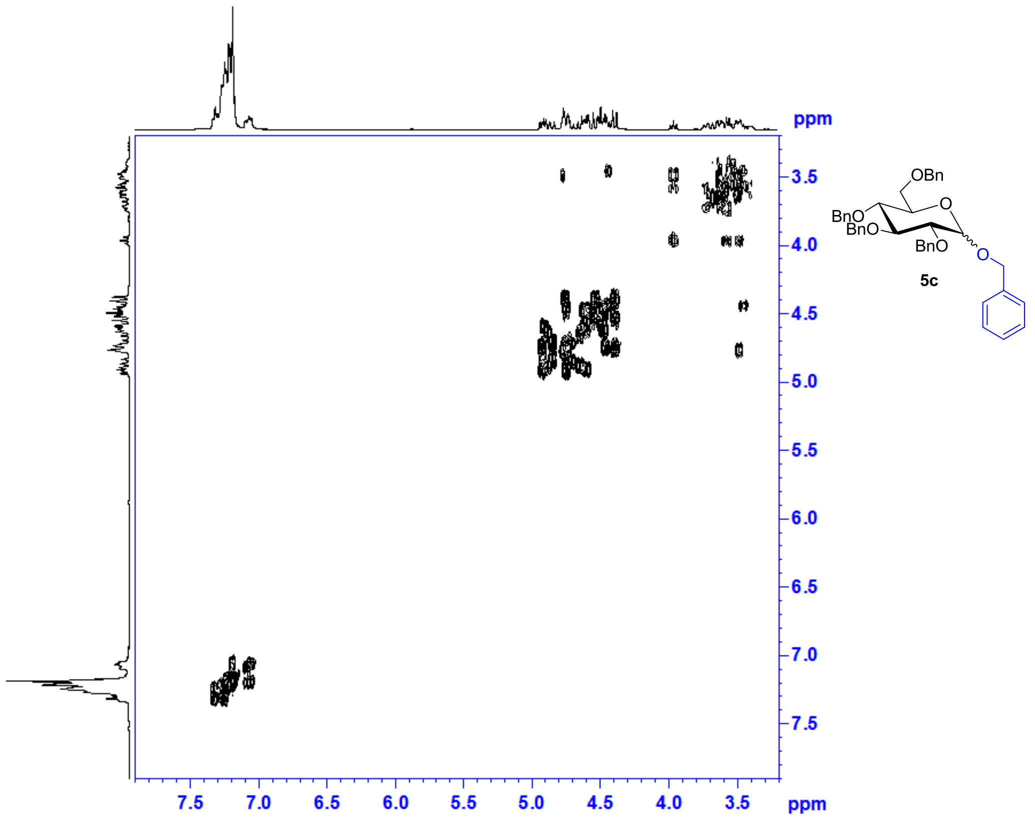

HSQC spectrum of compound **5c**

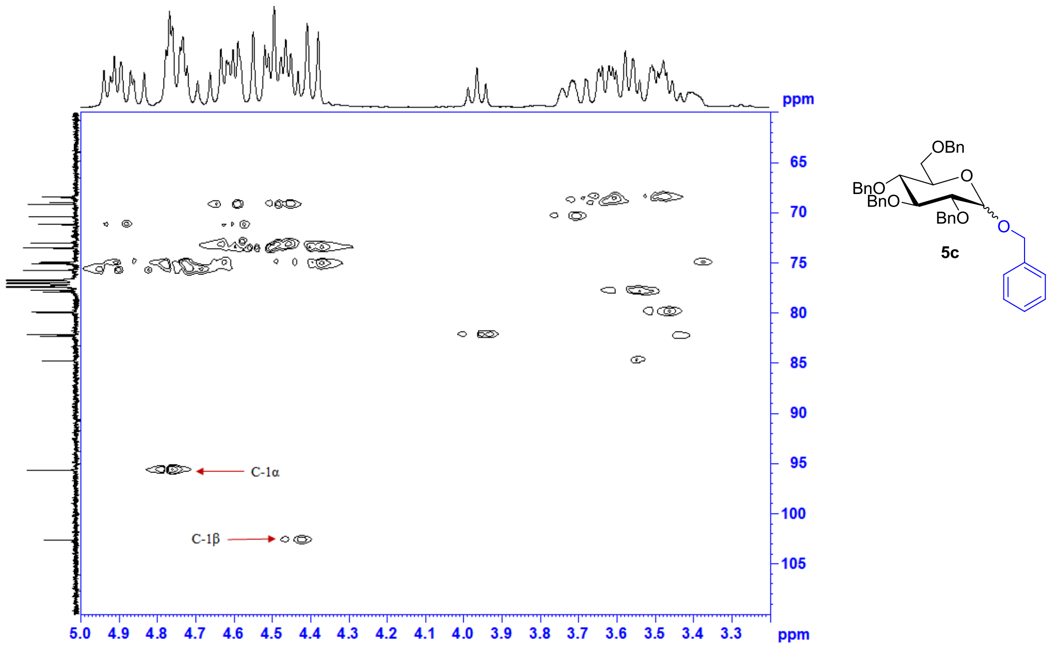

^{1}H NMR spectrum of compound **5d** (400 MHz, $CDCl_3$)

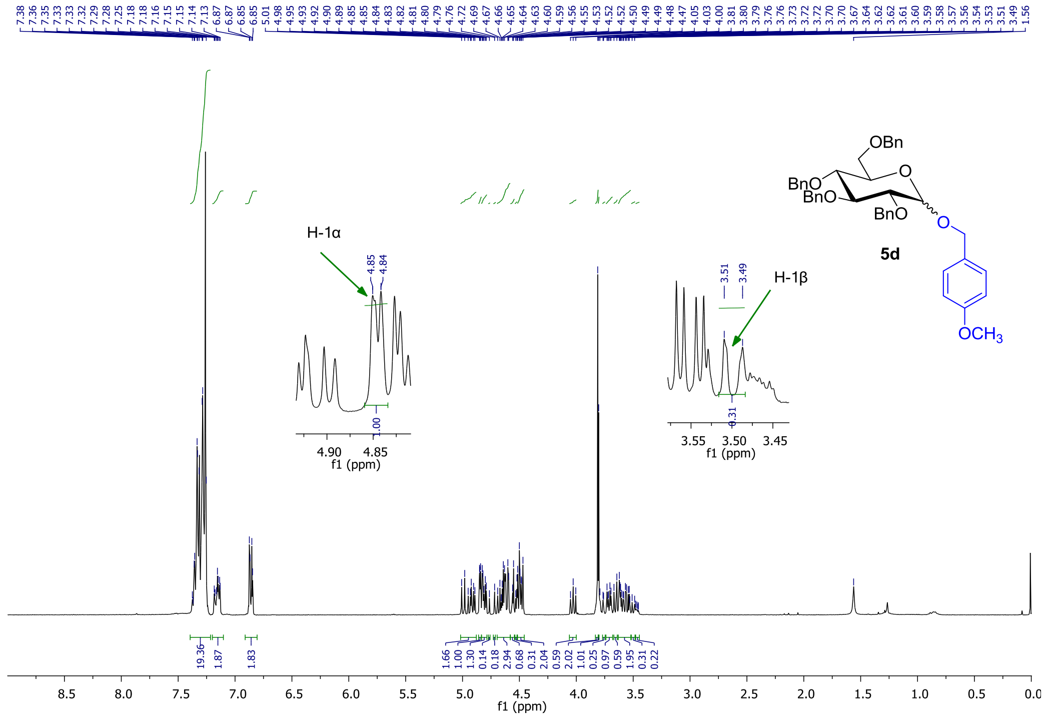

^{13}C NMR spectrum of compound **5d** (100 MHz, $CDCl_3$)

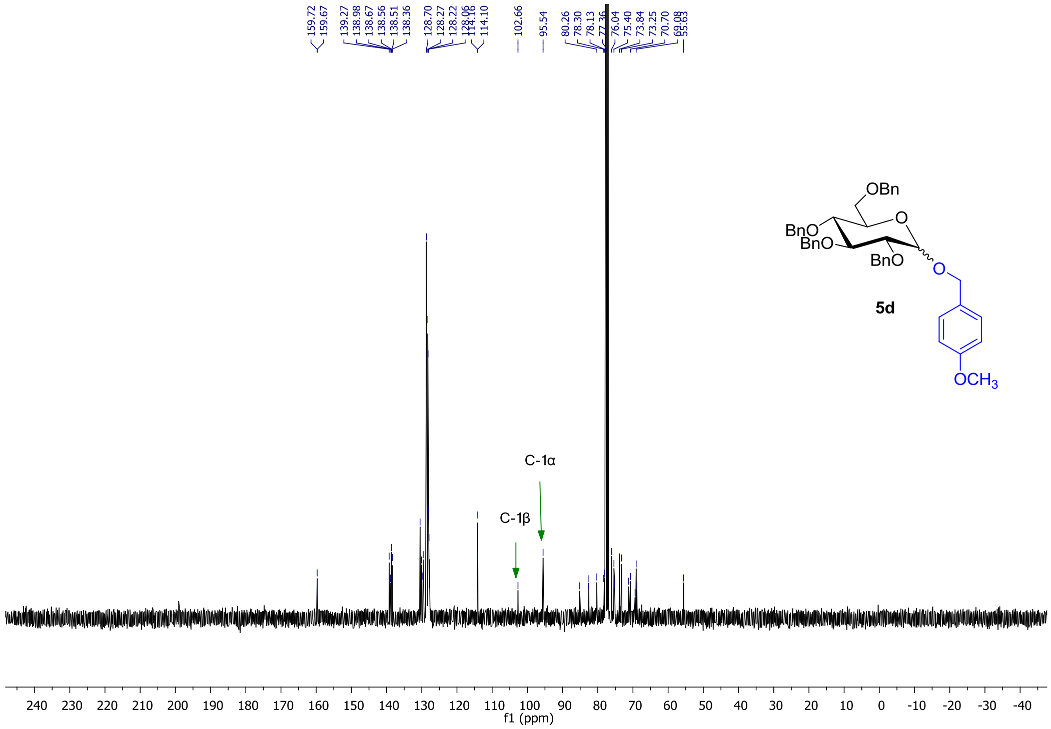

COSY spectrum of compound **5d**

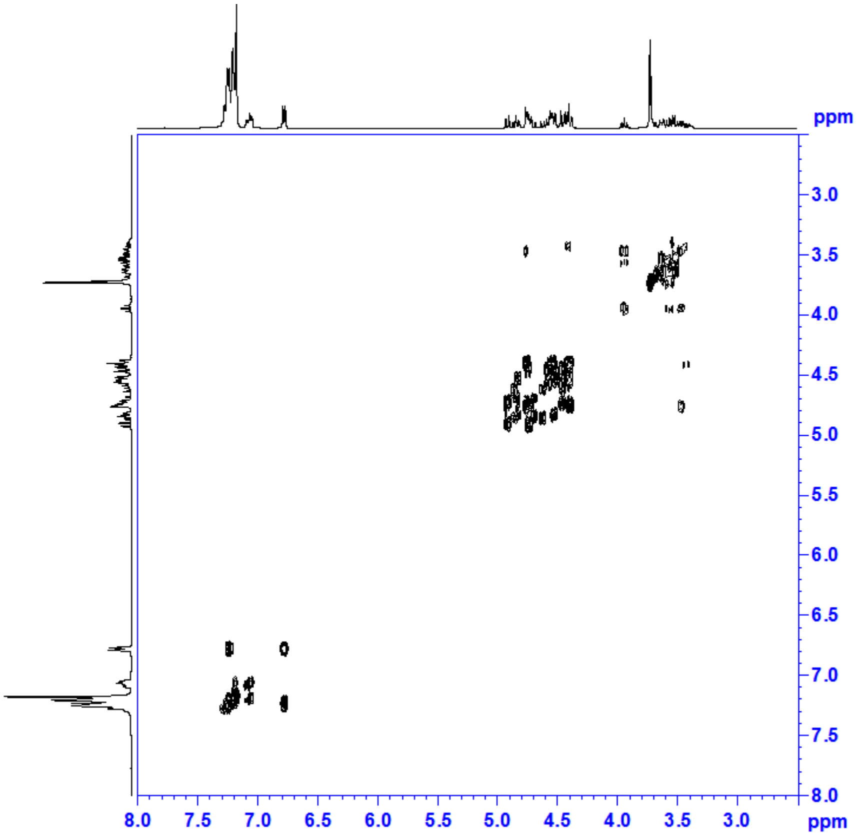

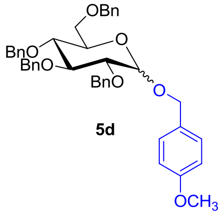

5d

HSQC spectrum of compound **5d**

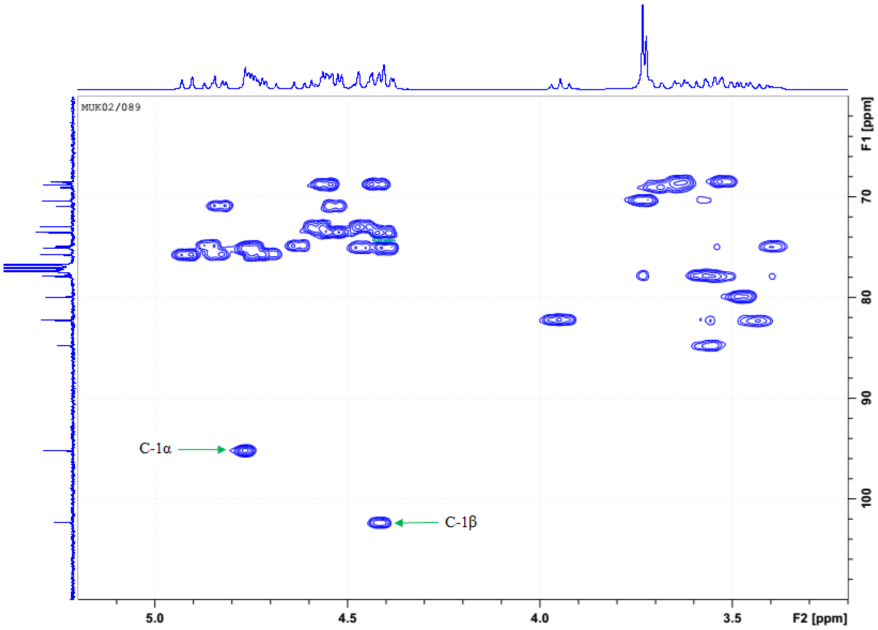

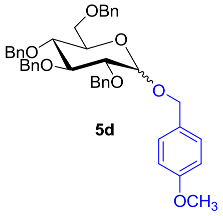

5d

^{1}H NMR spectrum of compound **5e** (400 MHz, $CDCl_3$)

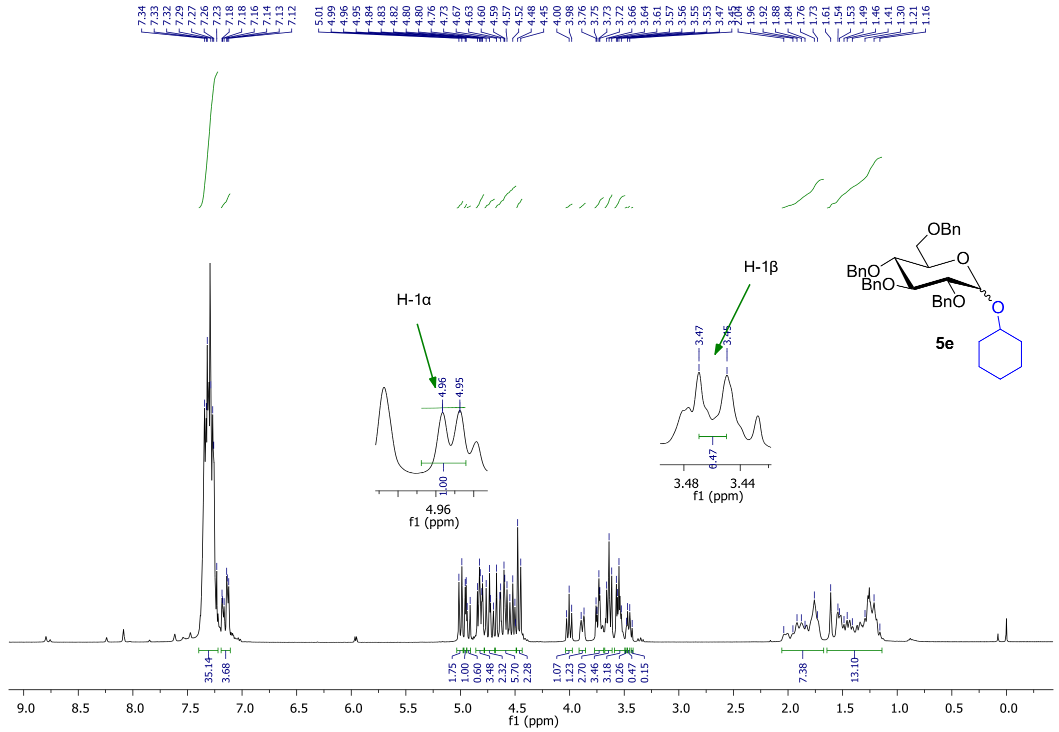

^{13}C NMR spectrum of compound **5e** (100 MHz, $CDCl_3$)

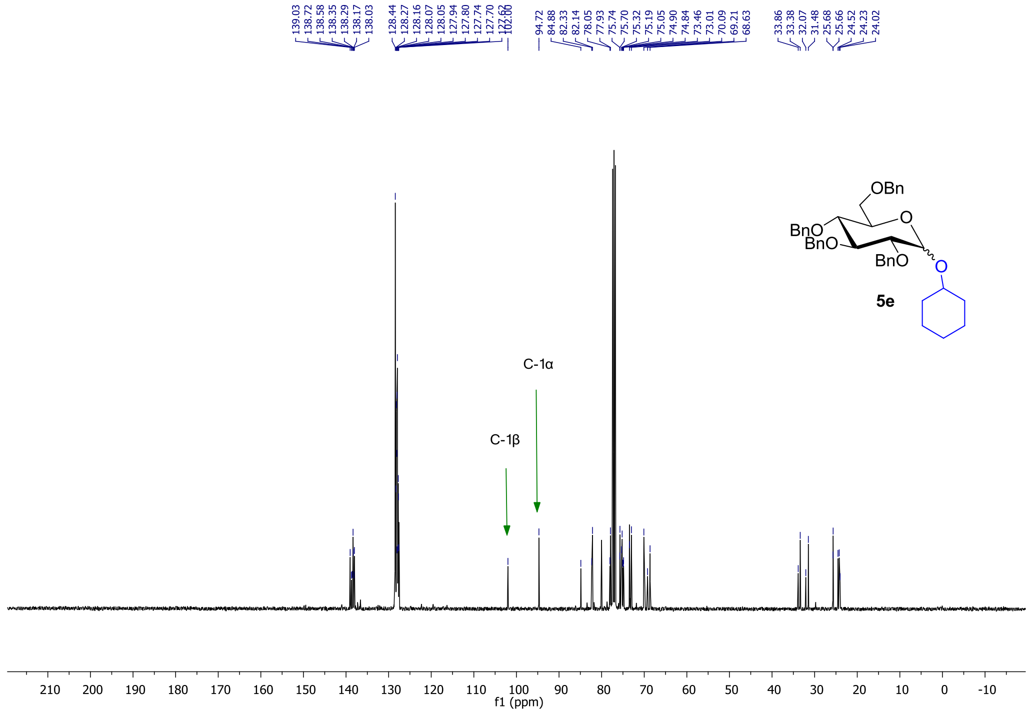

COSY spectrum of compound **5e**

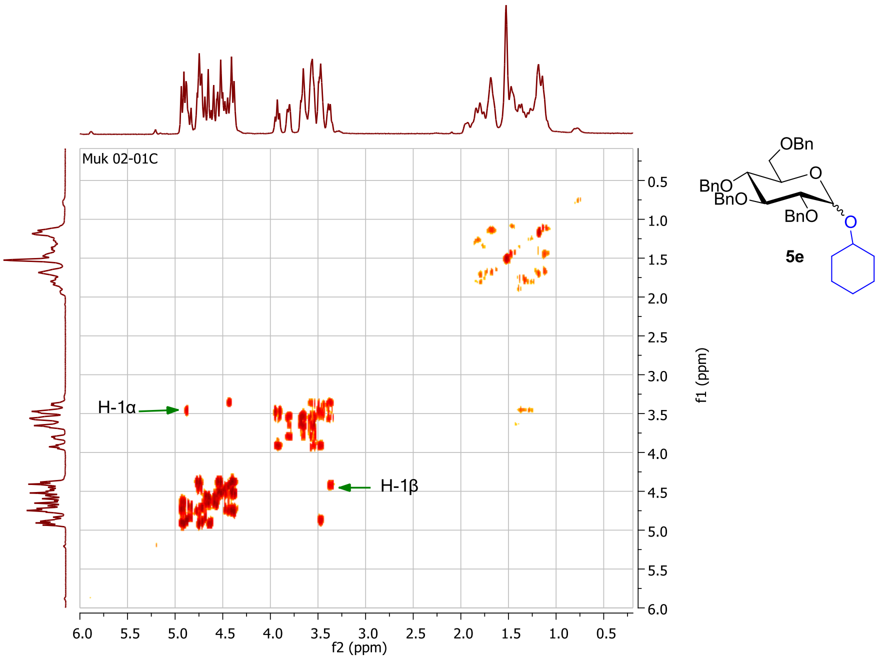

HSQC spectrum of compound **5e**

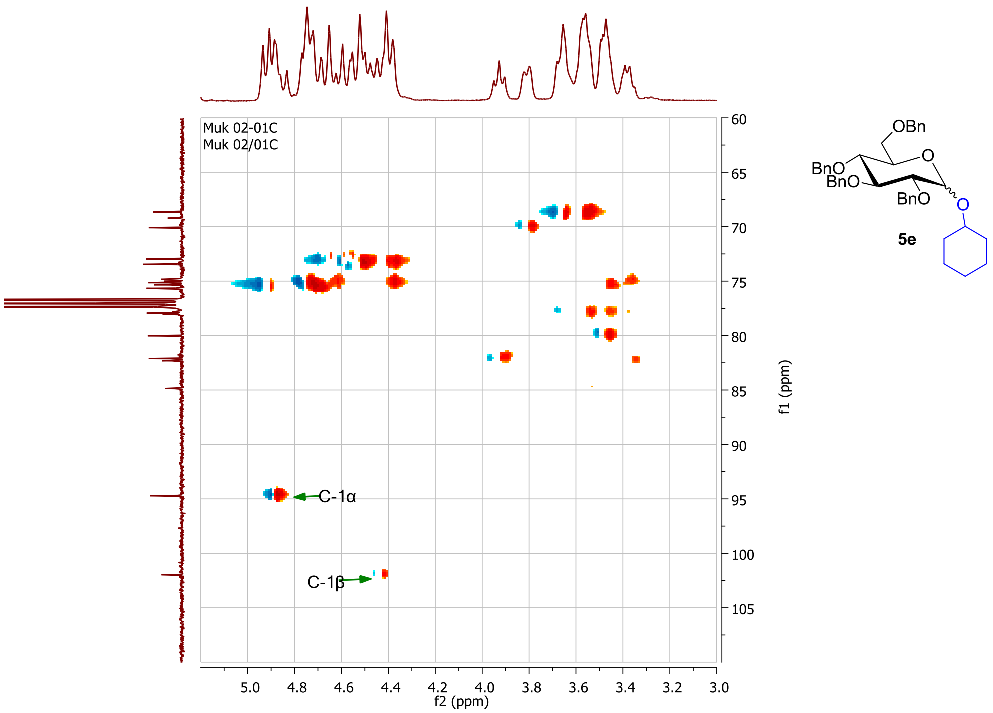

^{1}H NMR spectrum of compound **5f** (400 MHz, $CDCl_3$)

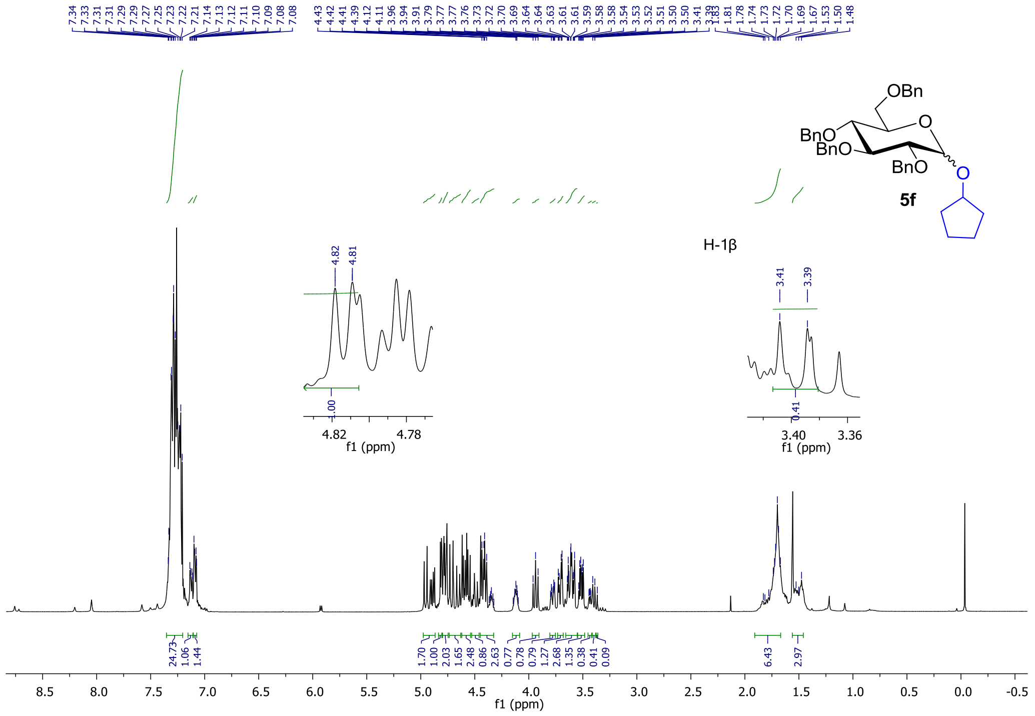

^{13}C NMR spectrum of compound **5f** (100 MHz, $CDCl_3$)

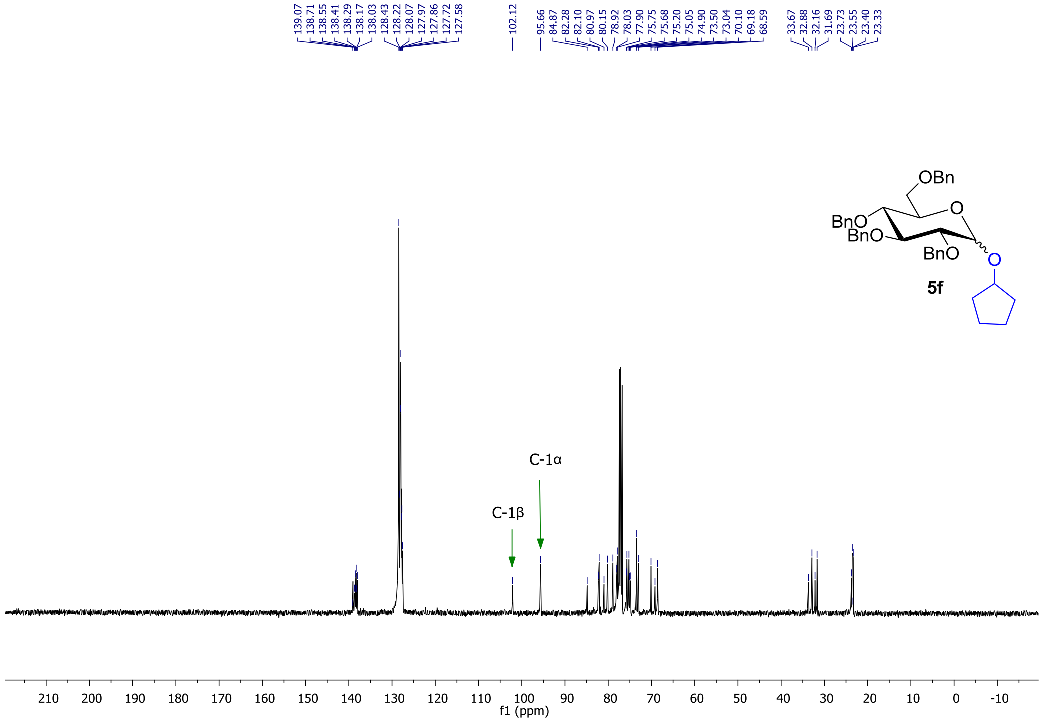

COSY spectrum of compound **5f**

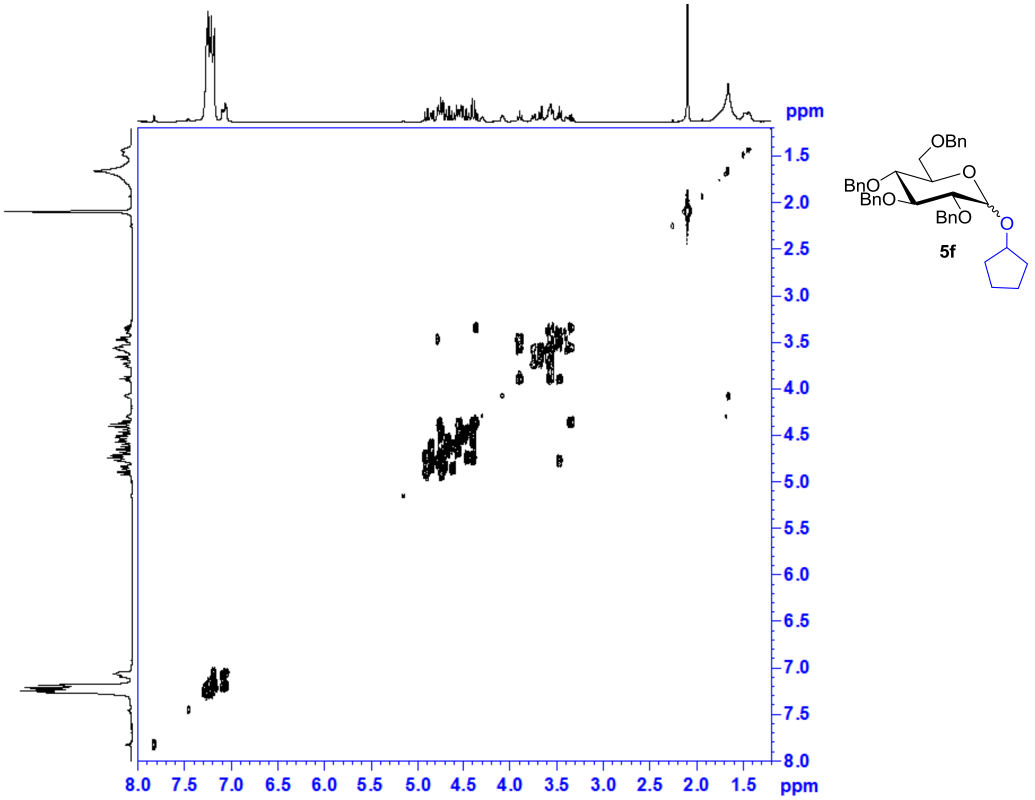

HSQC spectrum of compound **5f**

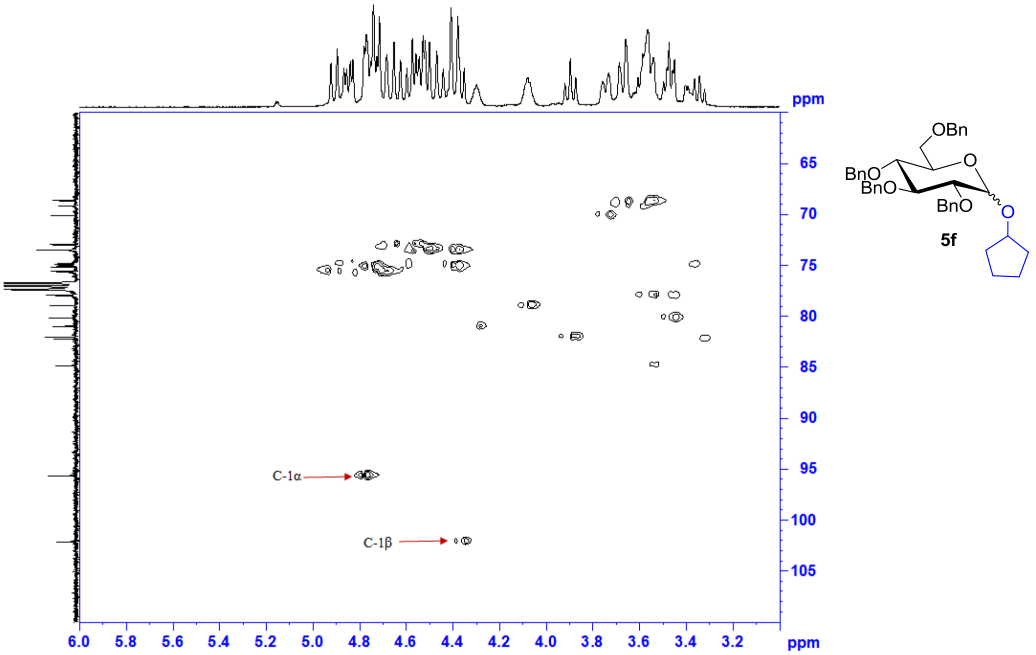

^{1}H NMR spectrum of compound **5g** (400 MHz, $CDCl_3$)

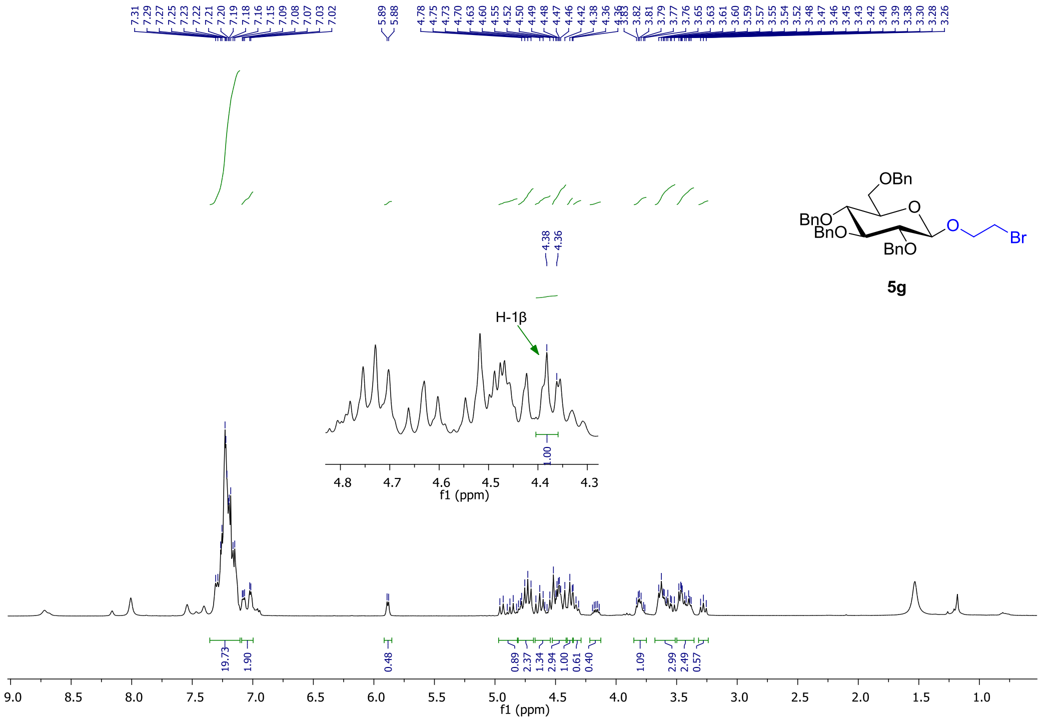

^{13}C NMR spectrum of compound **5g** (100 MHz, $CDCl_3$)

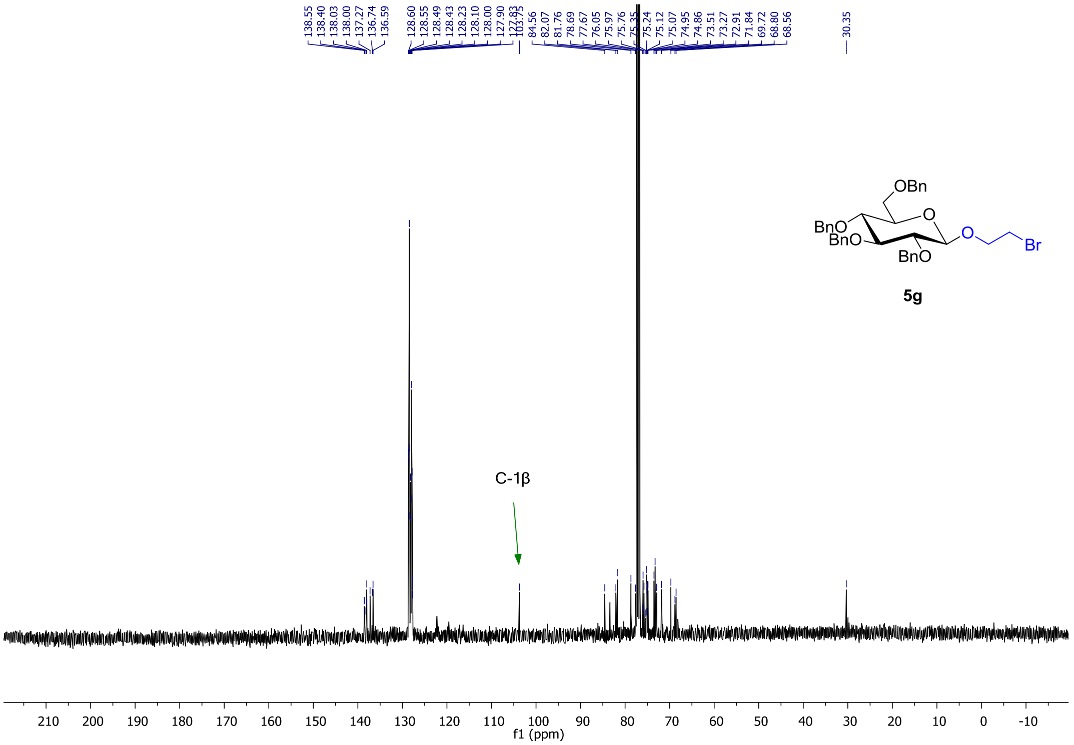

COSY spectrum of compound **5g**

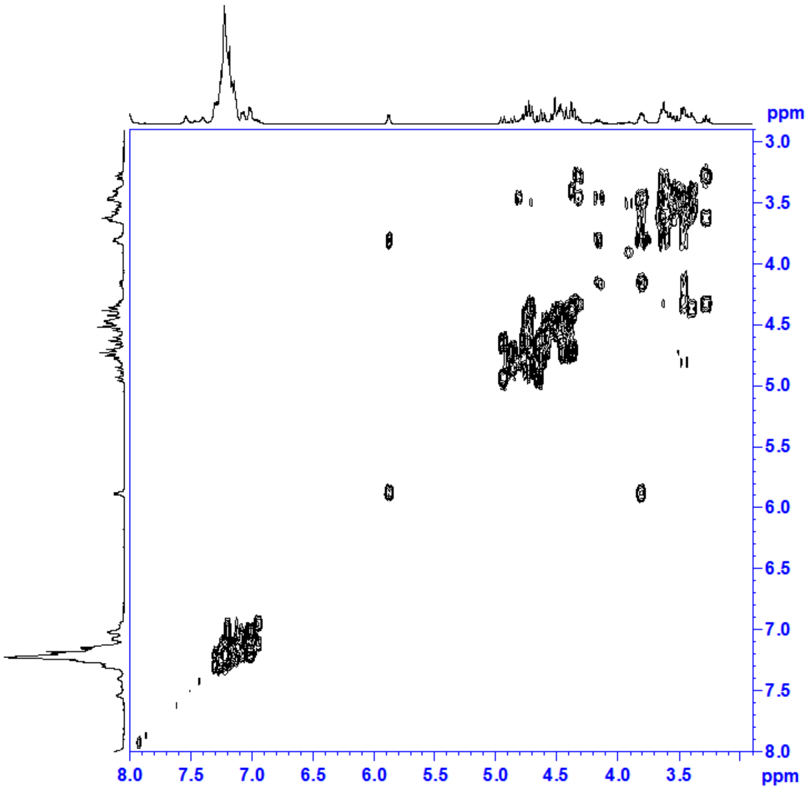

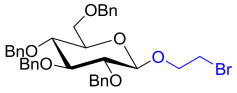

5g

HSQC spectrum of compound **5g**

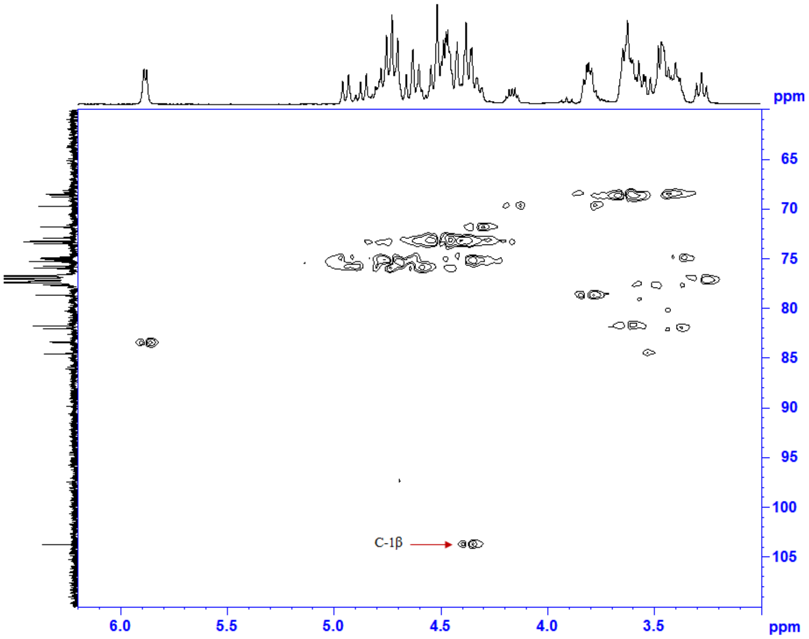

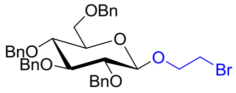

5g

^{1}H NMR spectrum of compound **5h** (400 MHz, $CDCl_3$)

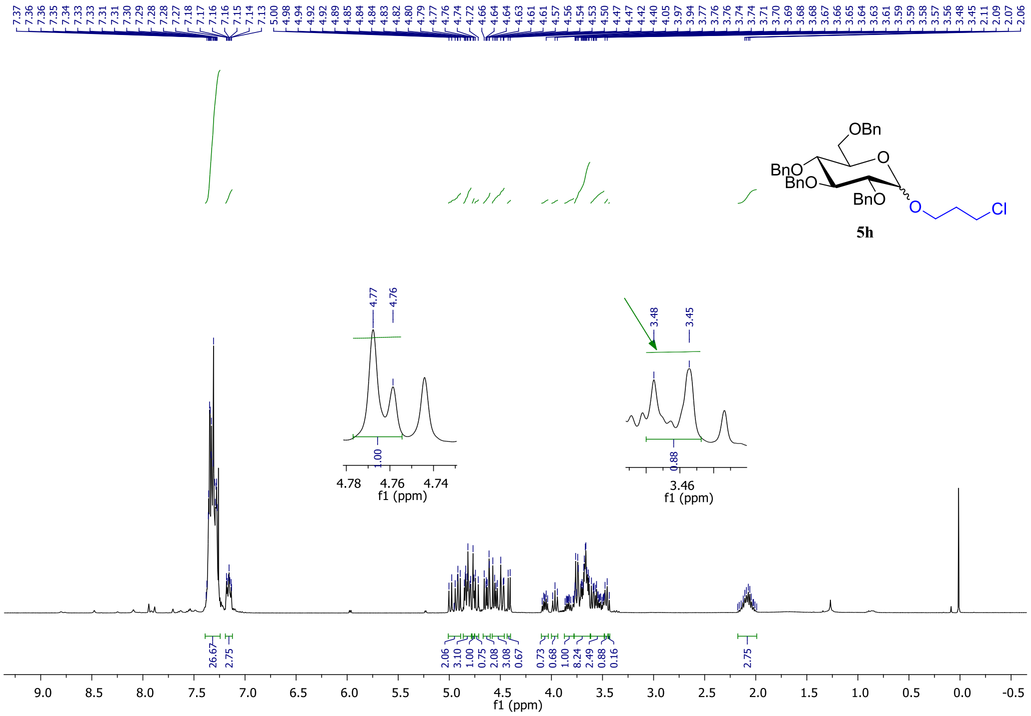

^{13}C NMR spectrum of compound **5h**(100 MHz, $CDCl_3$)

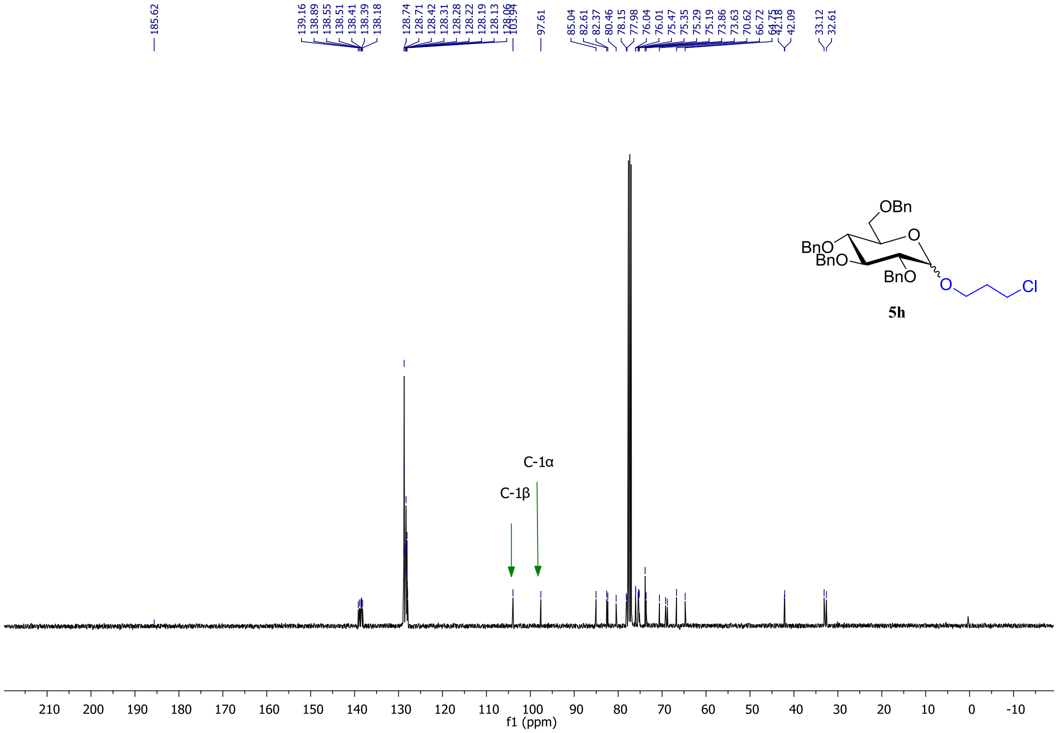

COSY spectrum of compound **5h**

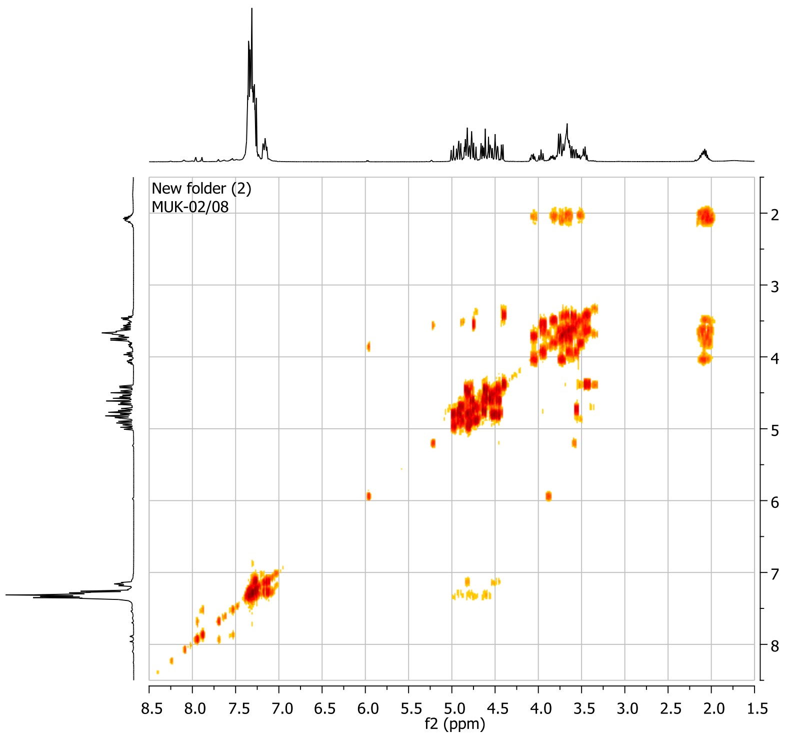

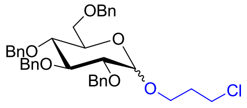

5h

HSQC spectrum of compound **5h**

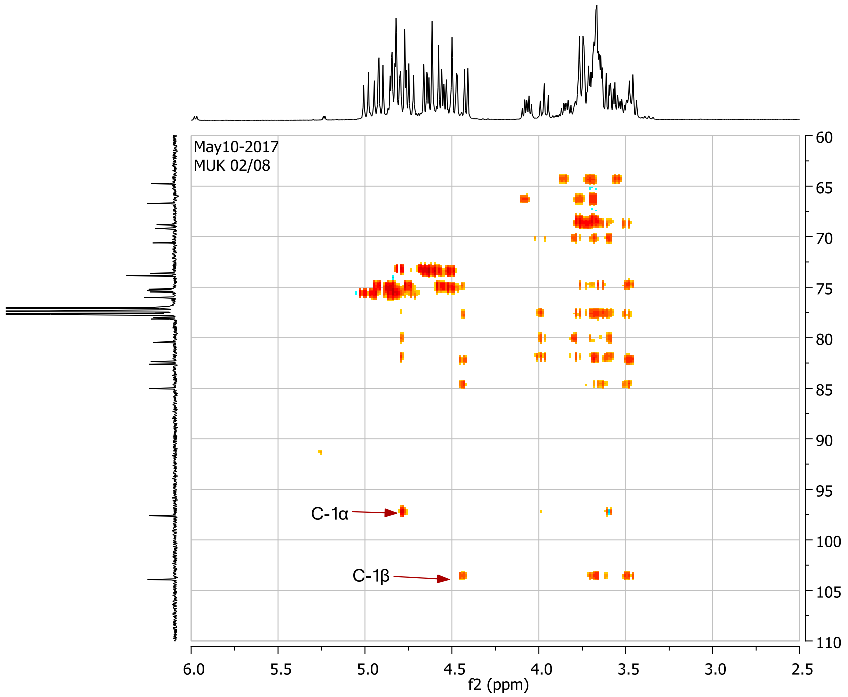

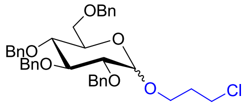

5h

^{1}H NMR spectrum of compound **5i** (400 MHz, $CDCl_3$)

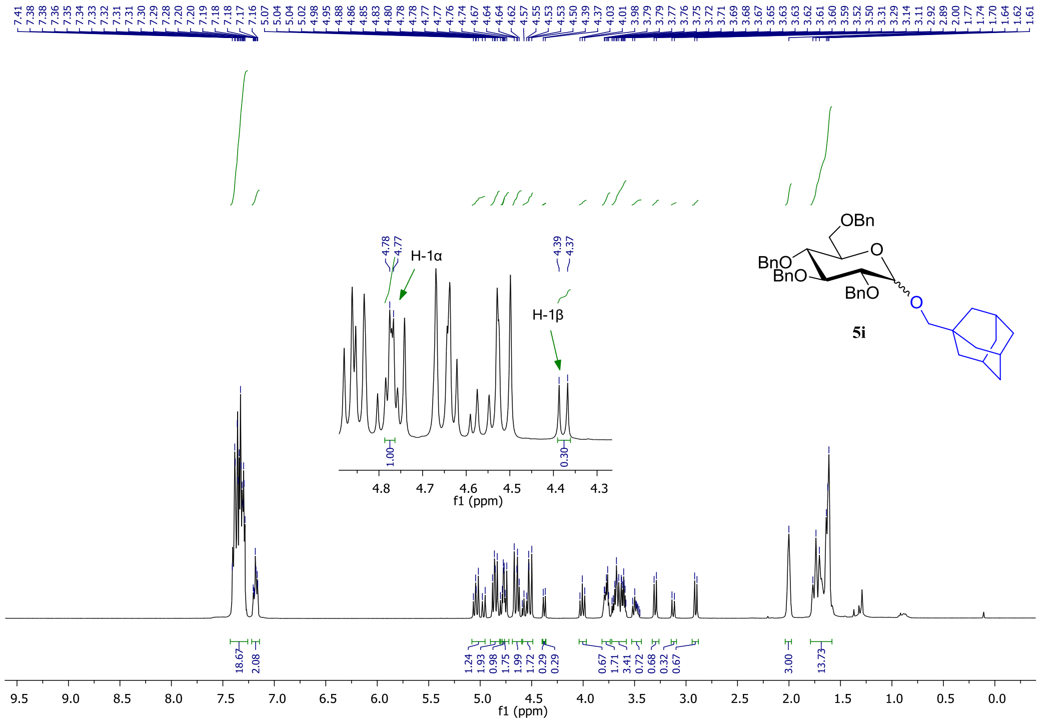

^{13}C NMR spectrum of compound **5i** (100 MHz, $CDCl_3$)

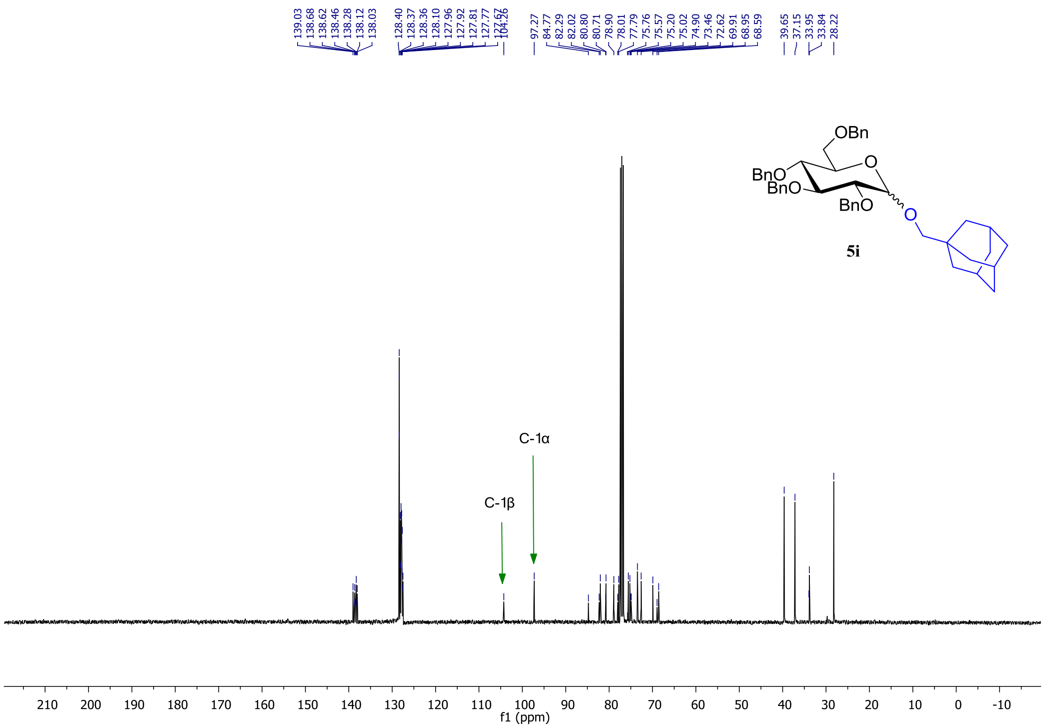

COSY spectrum of compound **5i**

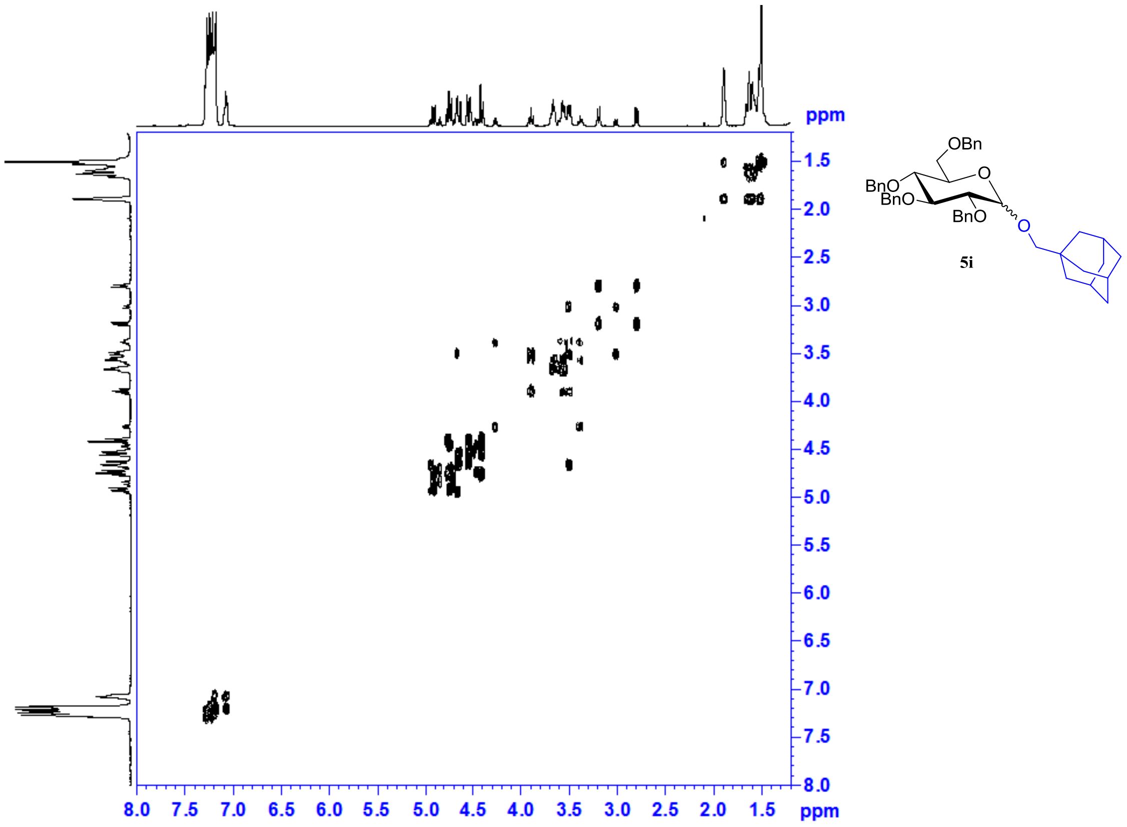

HSQC spectrum of compound **5i**

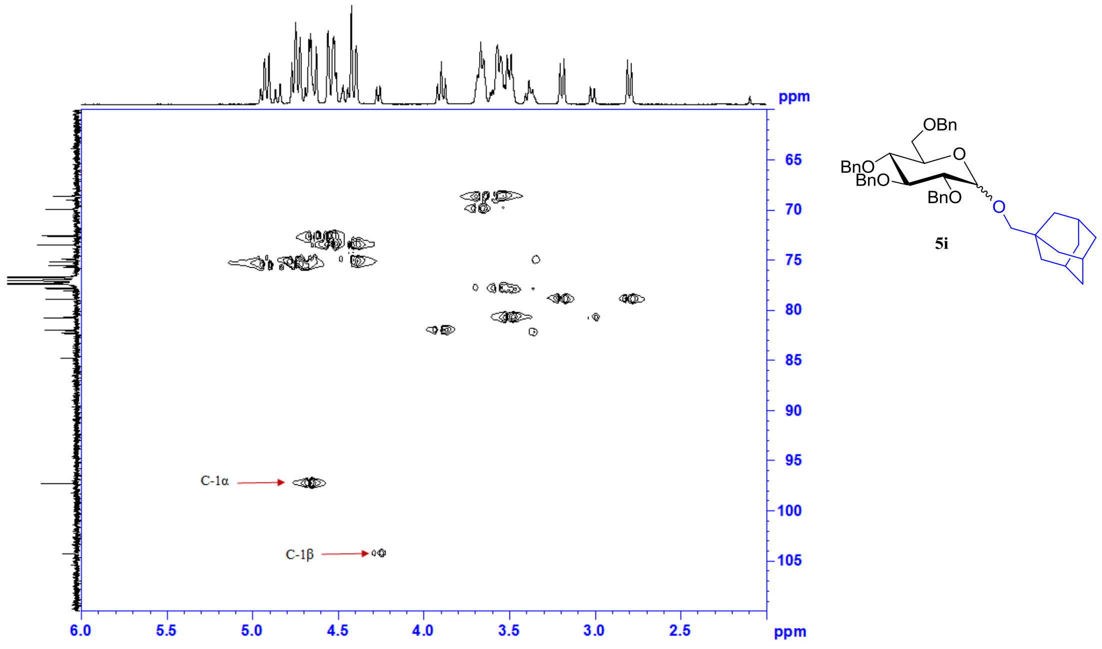

^{1}H NMR spectrum of compound **5j**(400 MHz, $CDCl_3$)

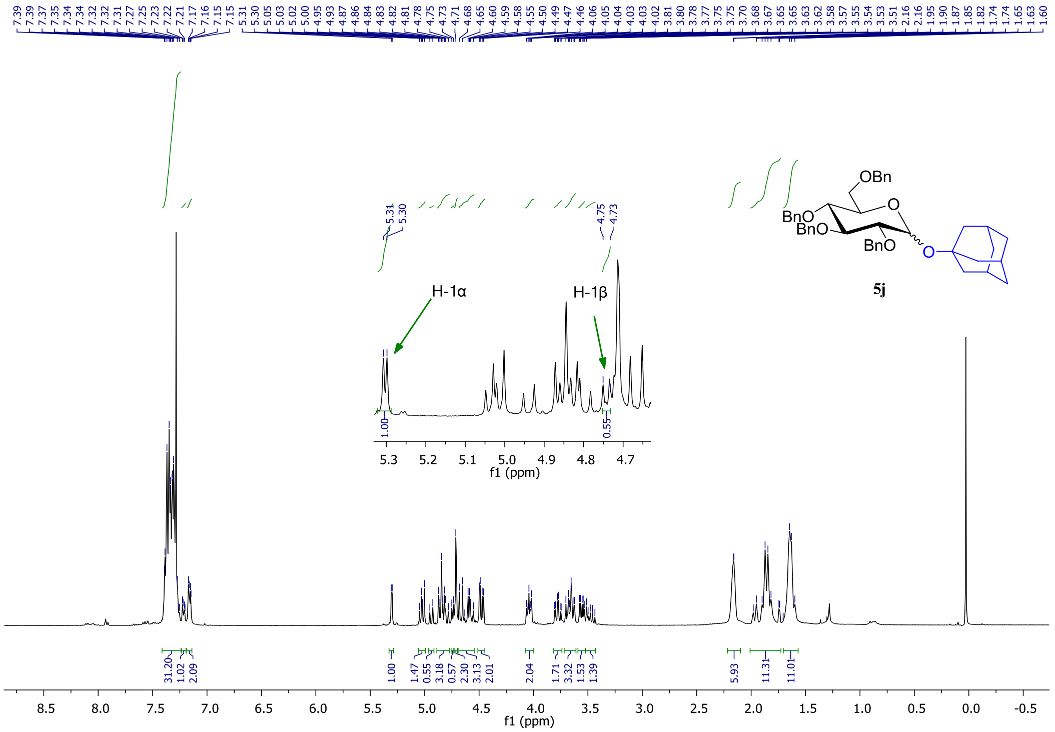

^{13}C NMR spectrum of compound **5j** (100 MHz, $CDCl_3$)

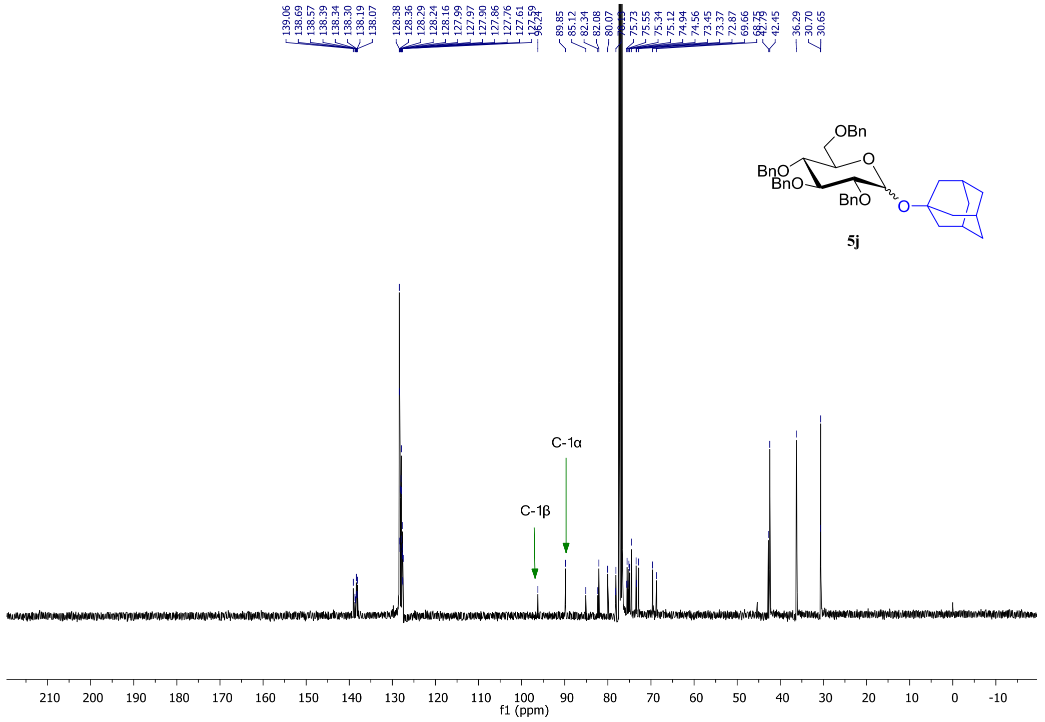

COSY spectrum of compound **5j**

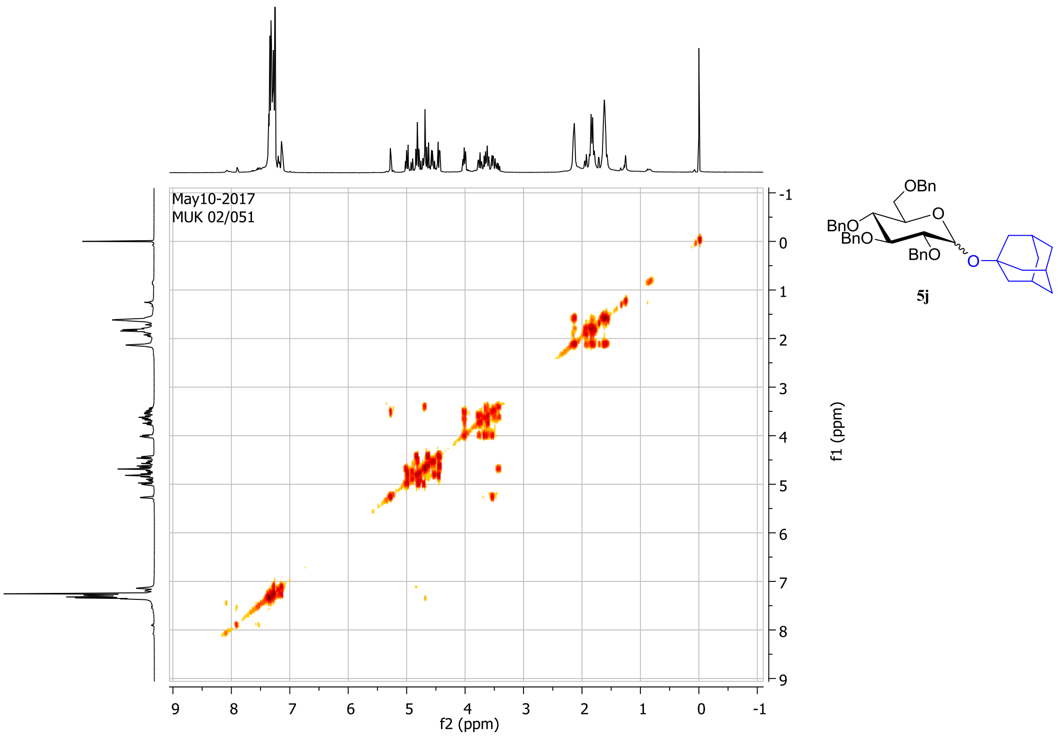

HSQC spectrum of compound **5j**

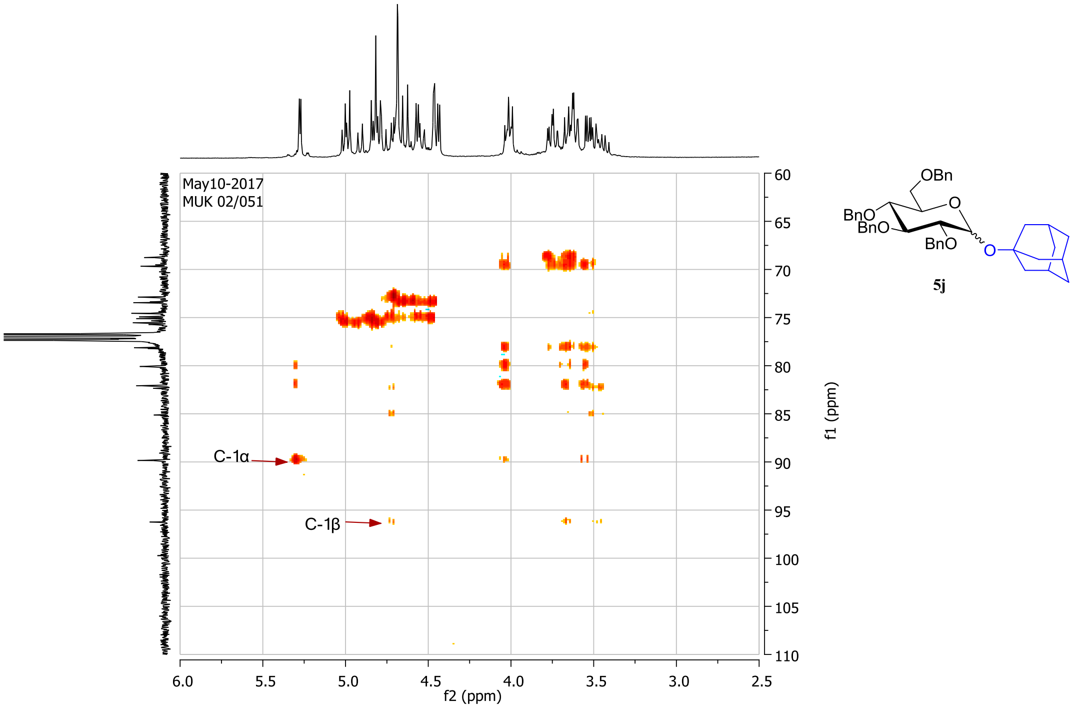

^{1}H NMR spectrum of compound **5k** (400 MHz, $CDCl_3$)

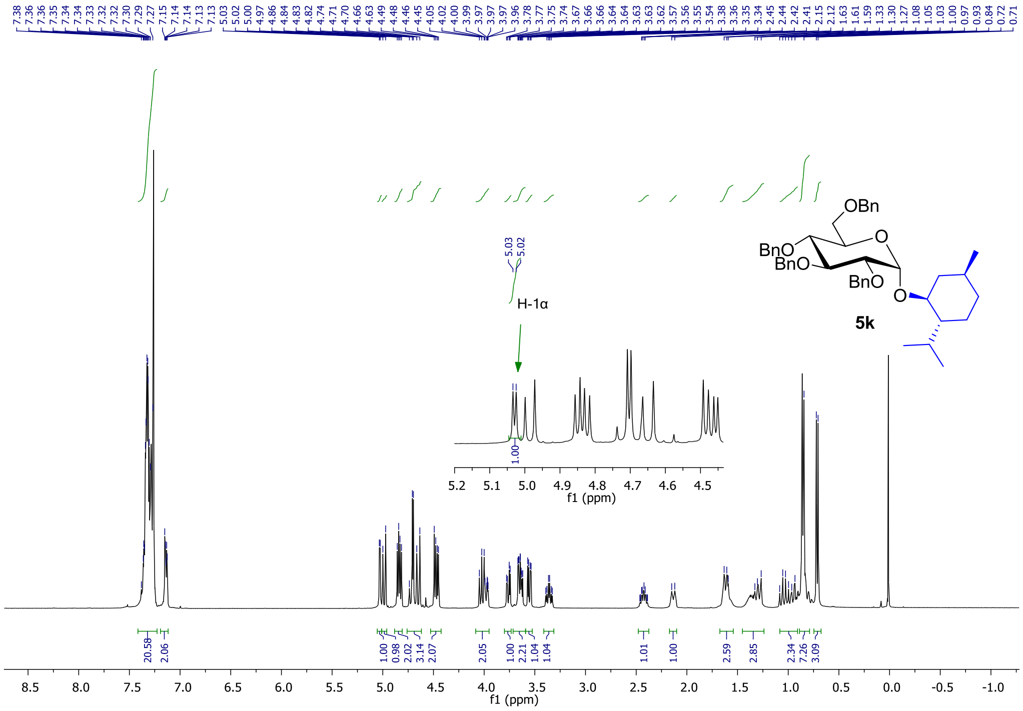

^{13}C NMR spectrum of compound **5k** (100 MHz, $CDCl_3$)

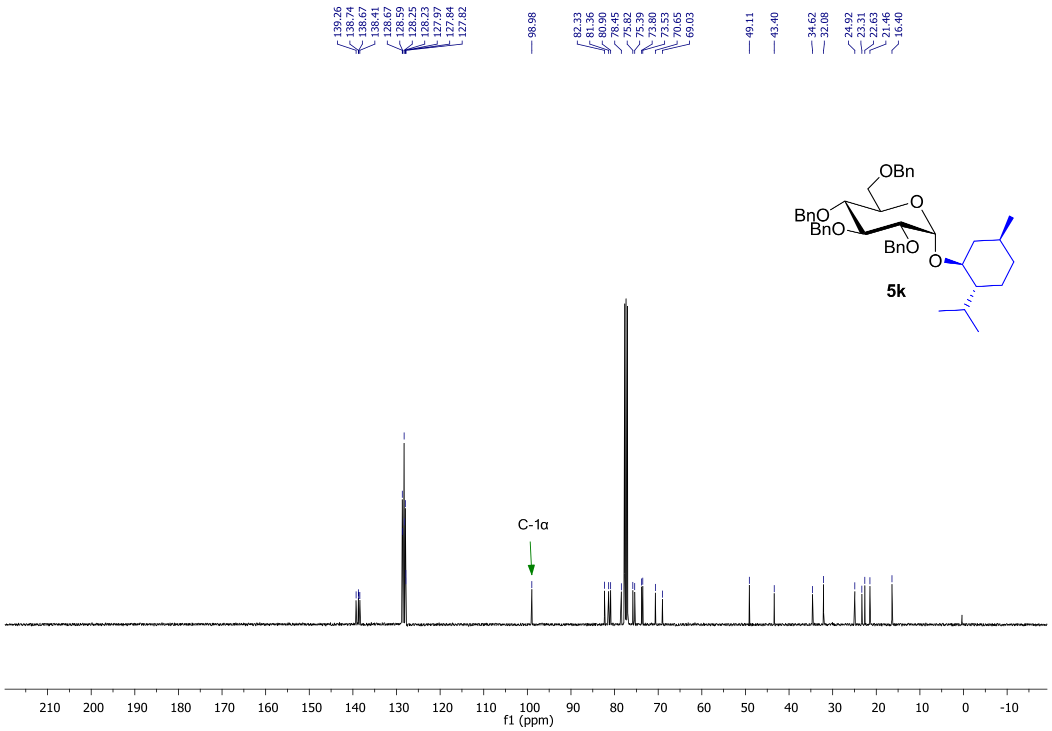

COSY spectrum of compound **5k**

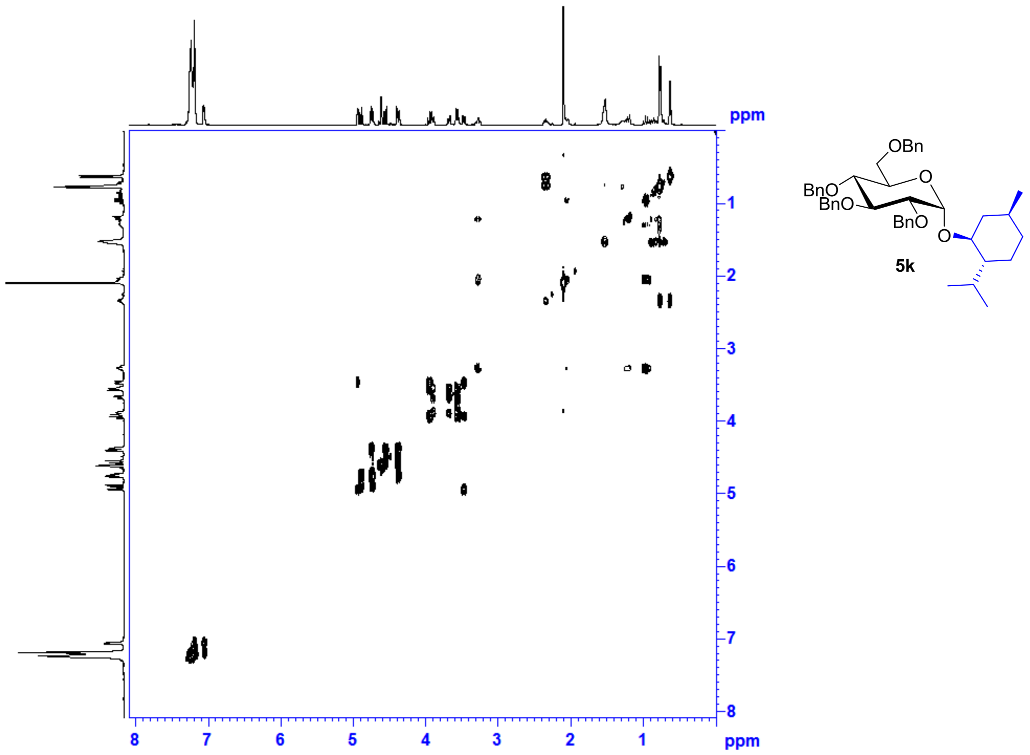

HSQC spectrum of compound **5k**

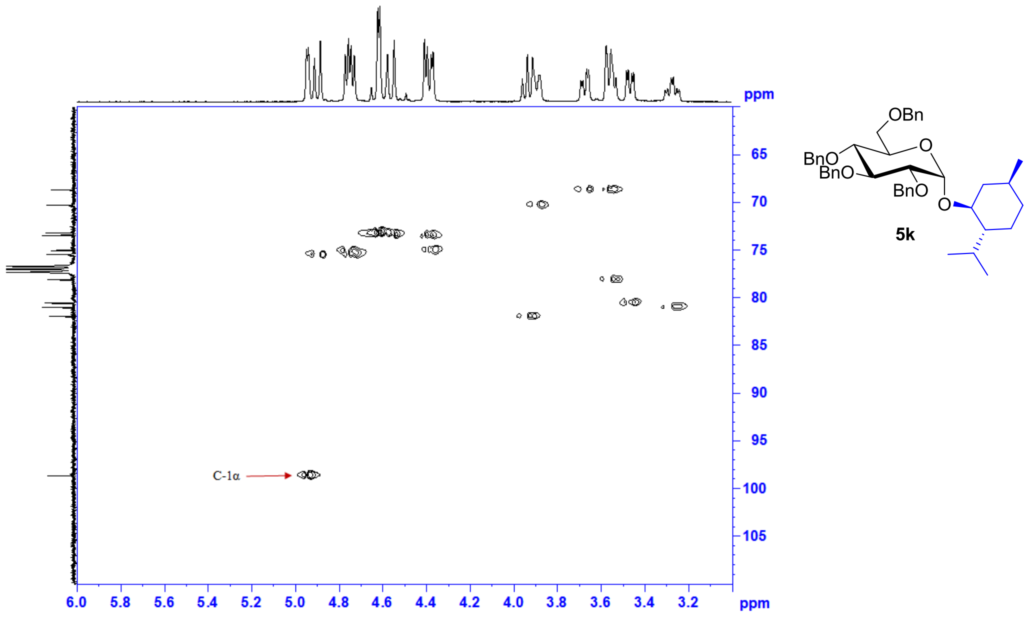

^{1}H NMR spectrum of compound **5l** (400 MHz, $CDCl_3$)

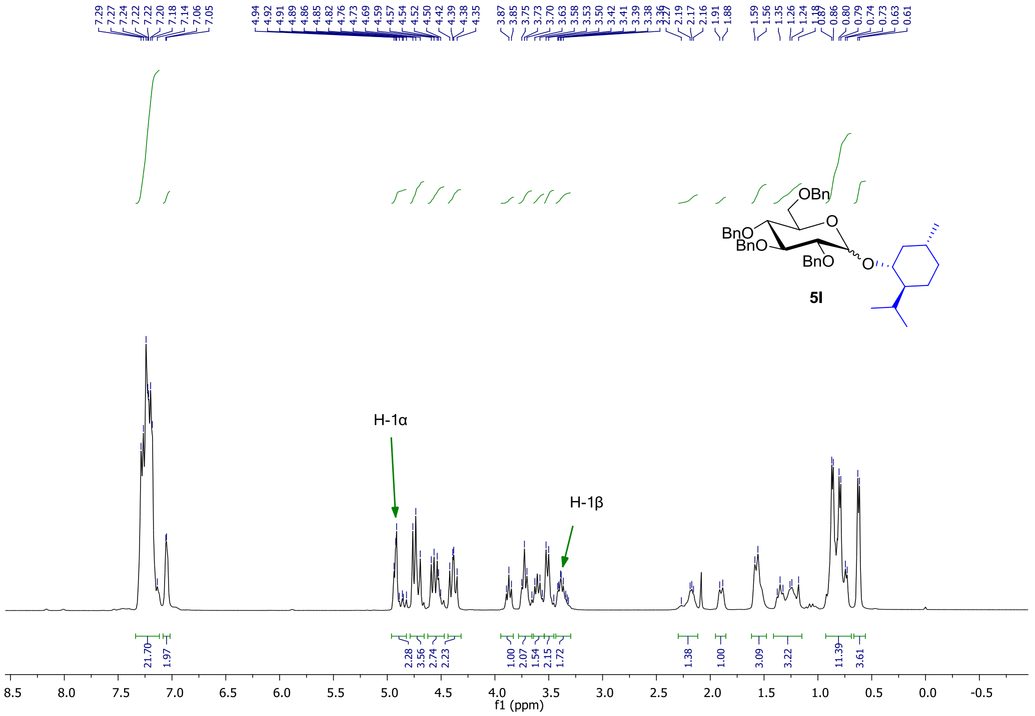

^{13}C NMR spectrum of compound **5l** (100 MHz, $CDCl_3$)

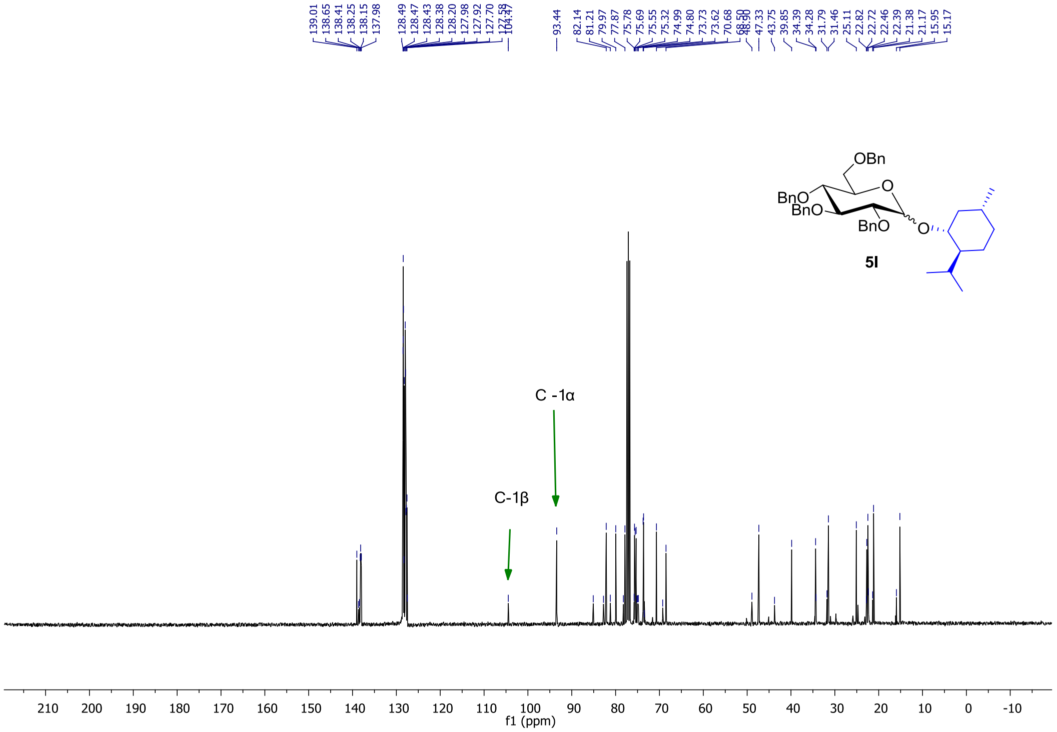

COSY spectrum of compound **5l**

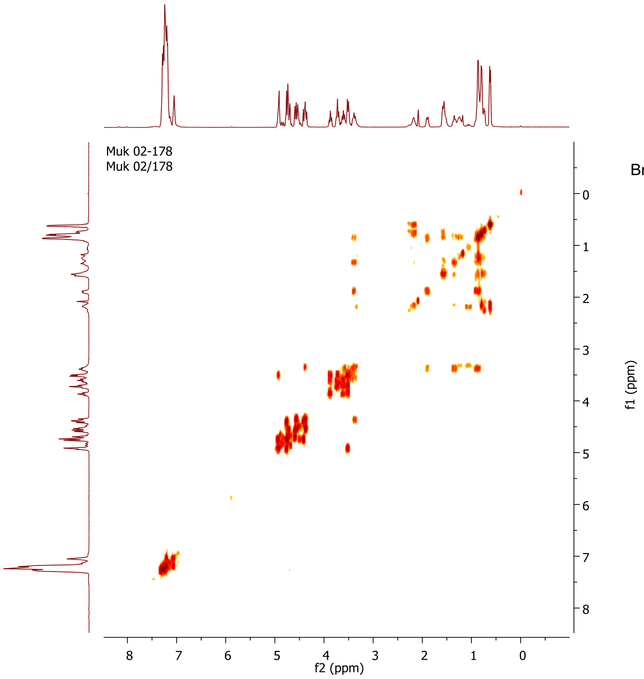

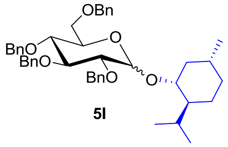

5l

HSQC spectrum of compound **5l**

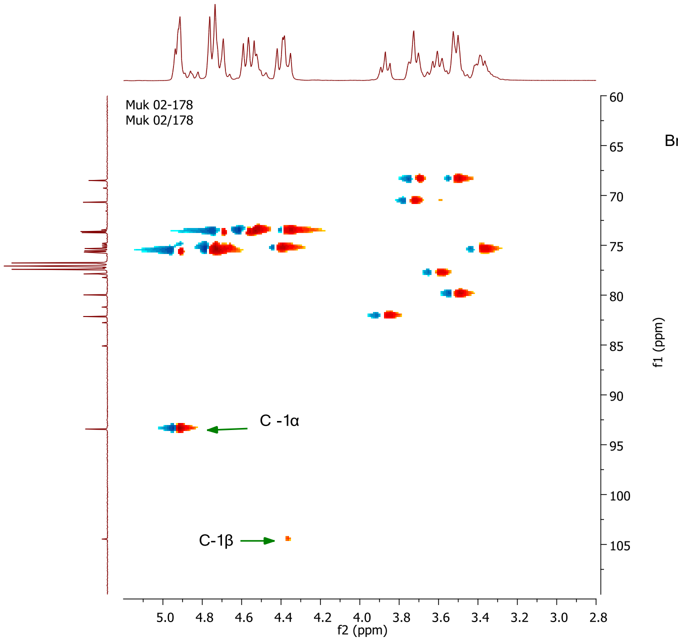

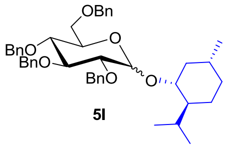

5l

^{1}H NMR spectrum of compound **5m** (400 MHz, $CDCl_3$)

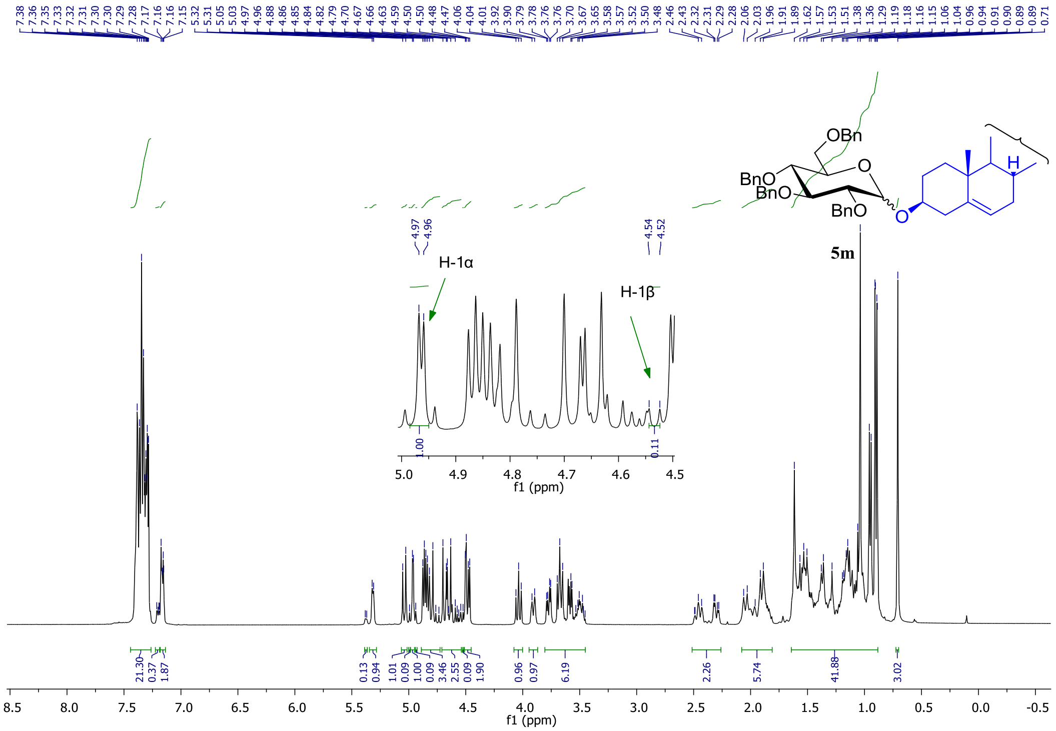

^{13}C NMR spectrum of compound **5m** (100 MHz, $CDCl_3$)

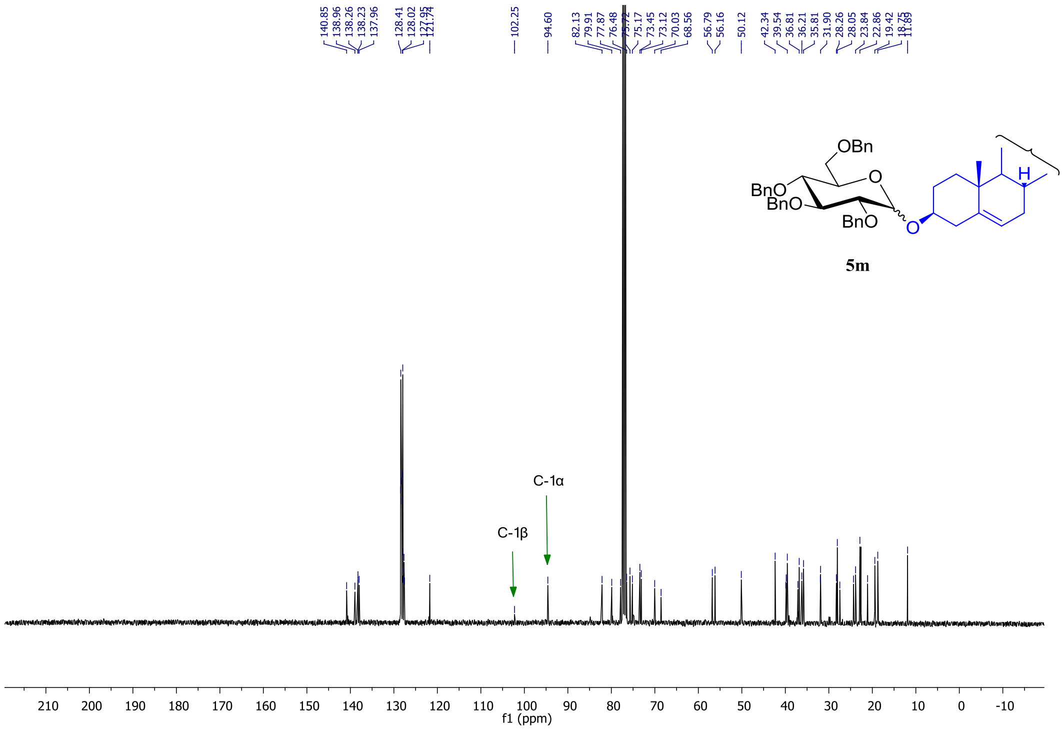

COSY spectrum of compound **5m**

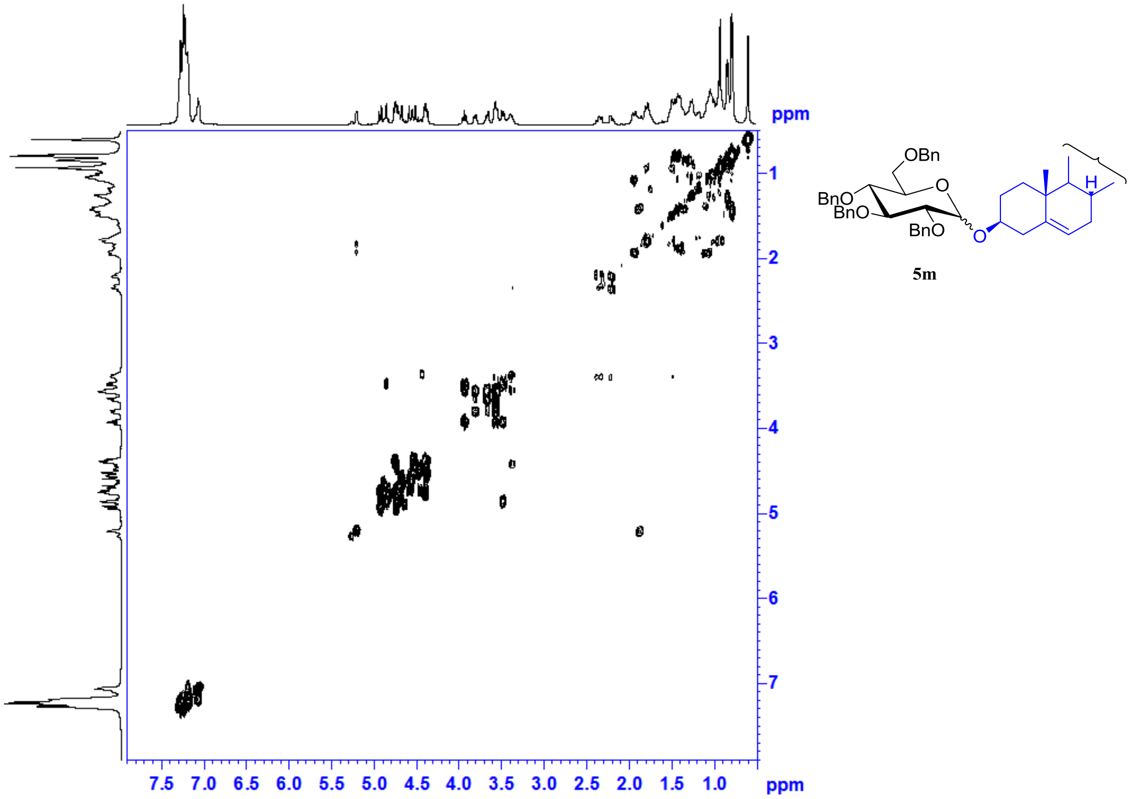

HSQC spectrum of compound **5m**

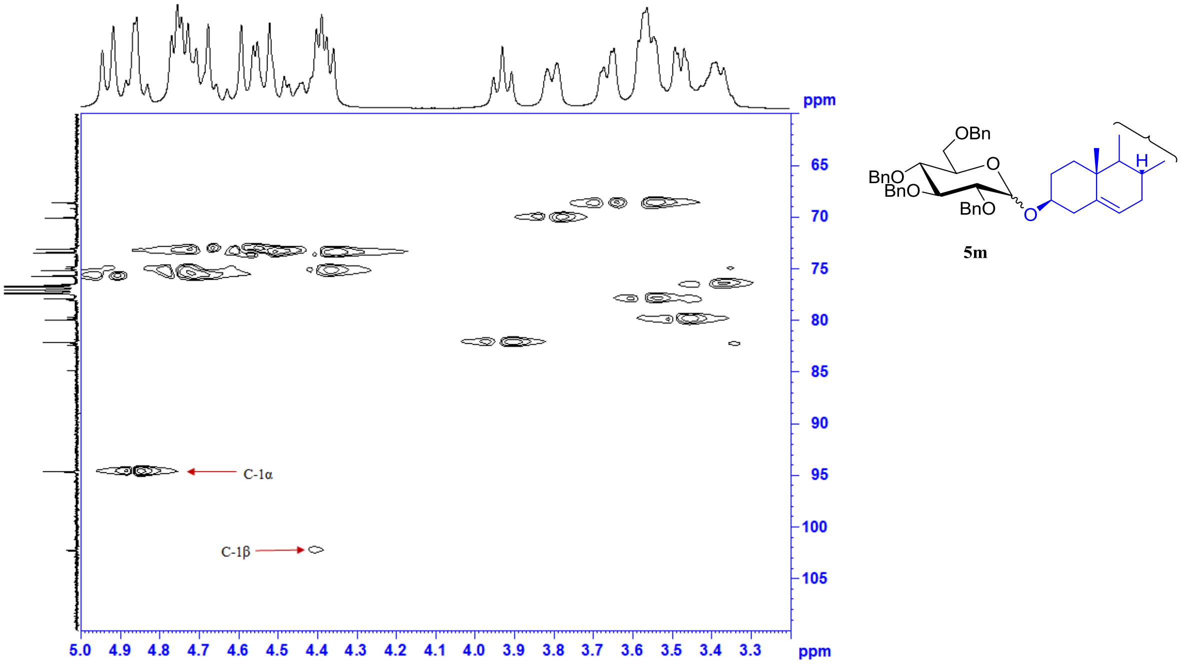

^{1}H NMR spectrum of compound **5n** (400 MHz, $CDCl_3$)

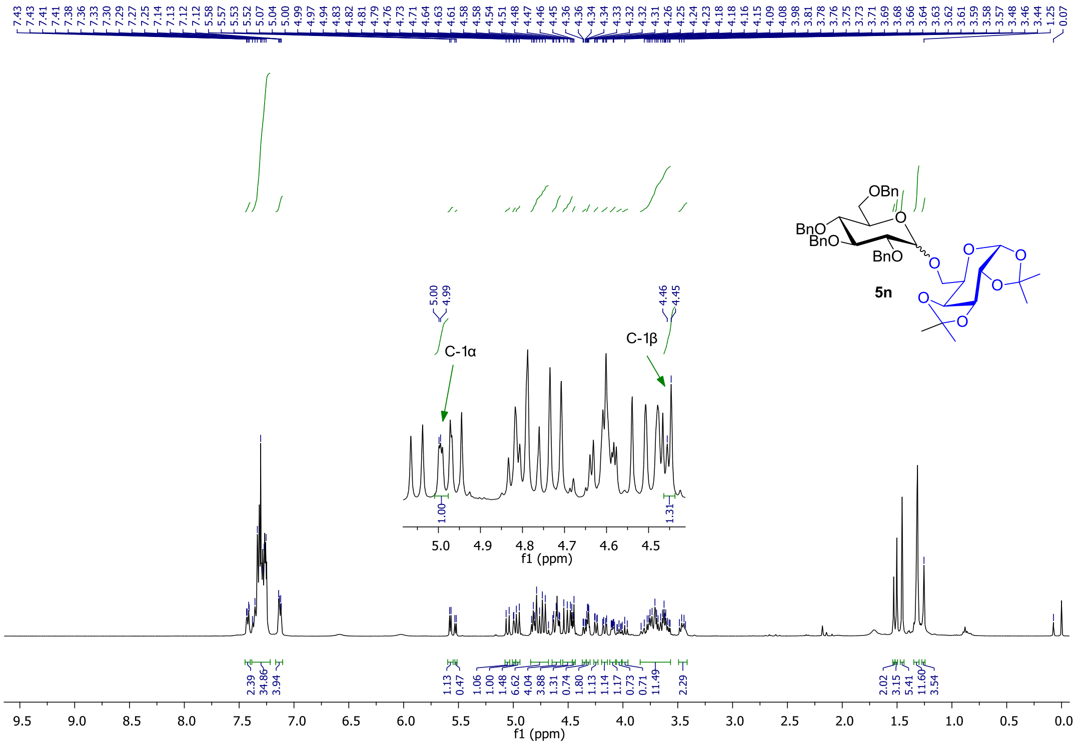

^{13}C NMR spectrum of compound **5n** (100 MHz, $CDCl_3$)

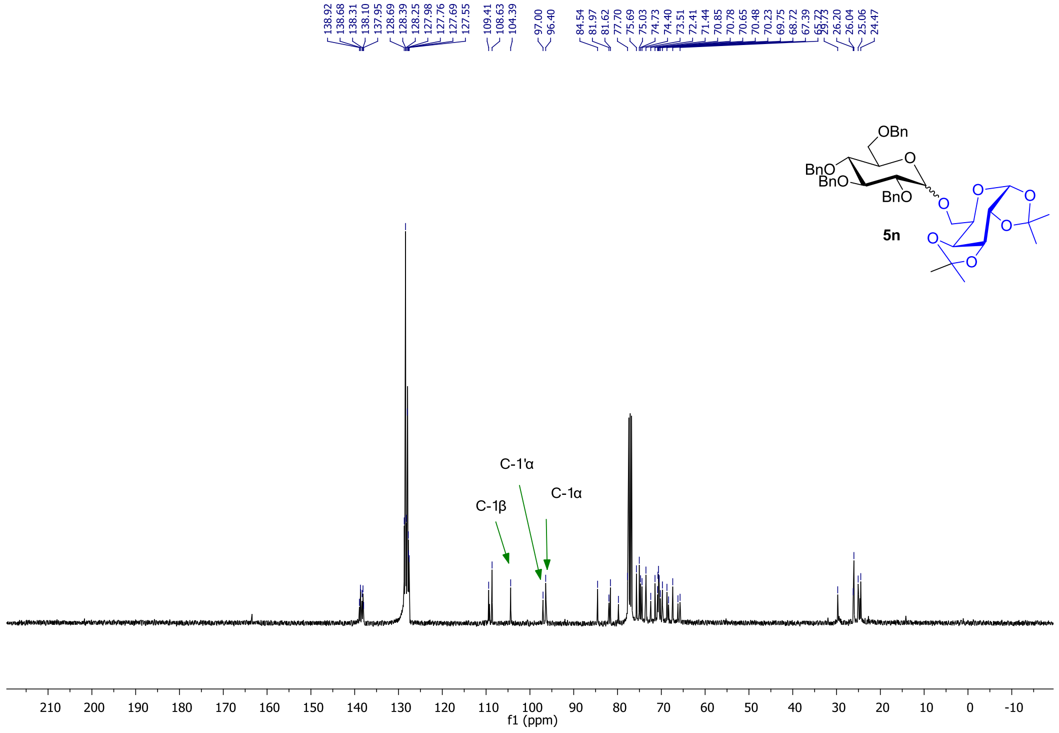

COSY spectrum of compound **5n**

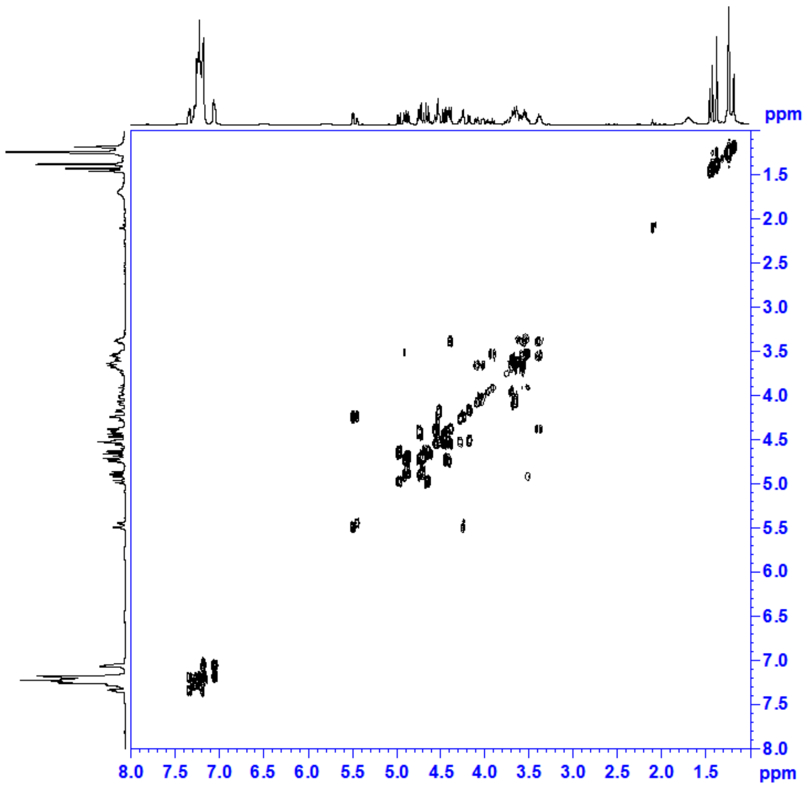

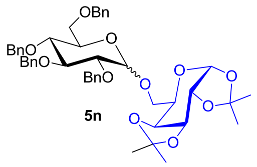

HSQC spectrum of compound **5n**

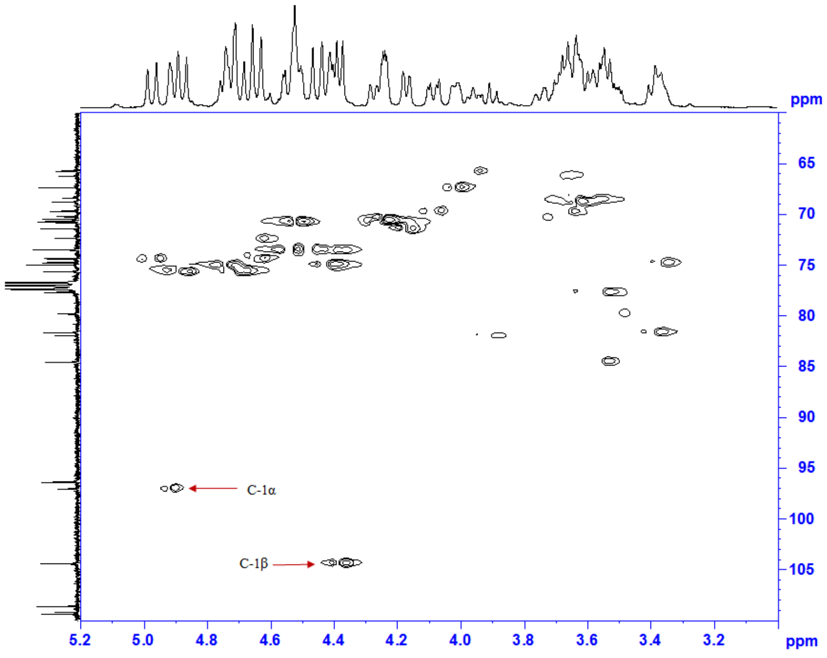

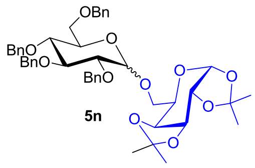

^{1}H NMR spectrum of compound **9** (400 MHz, $CDCl_3$)

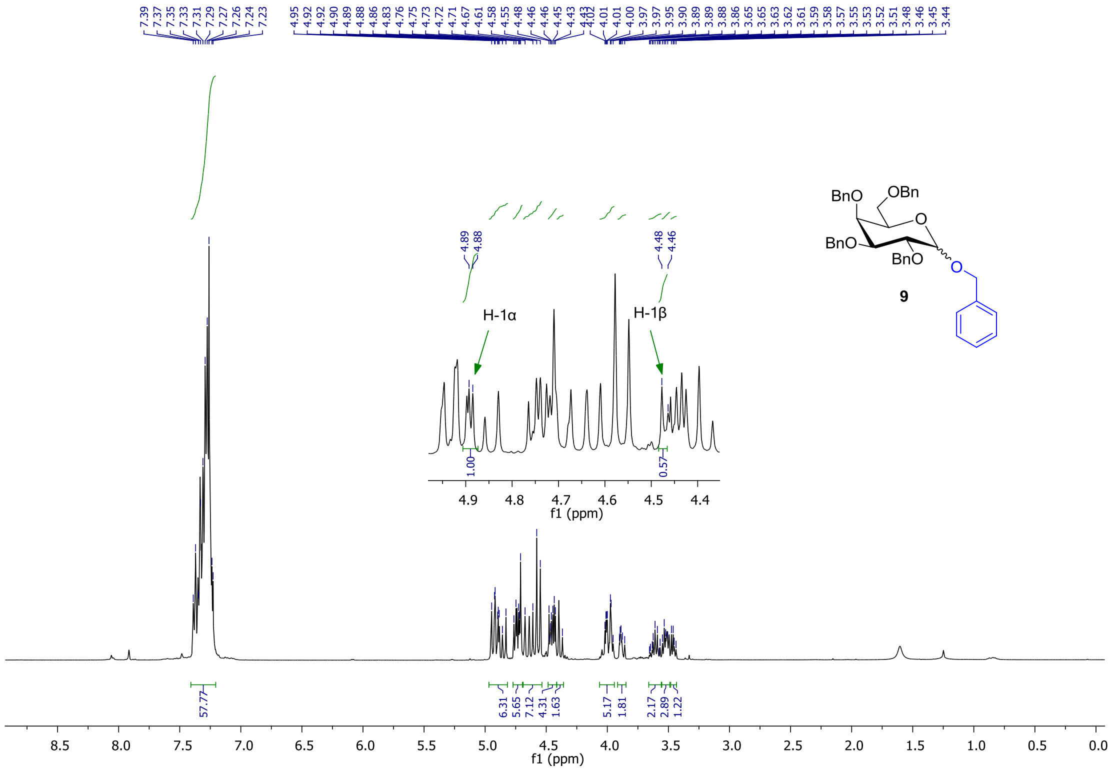

^{13}C NMR spectrum of compound **9** (100 MHz, $CDCl_3$)

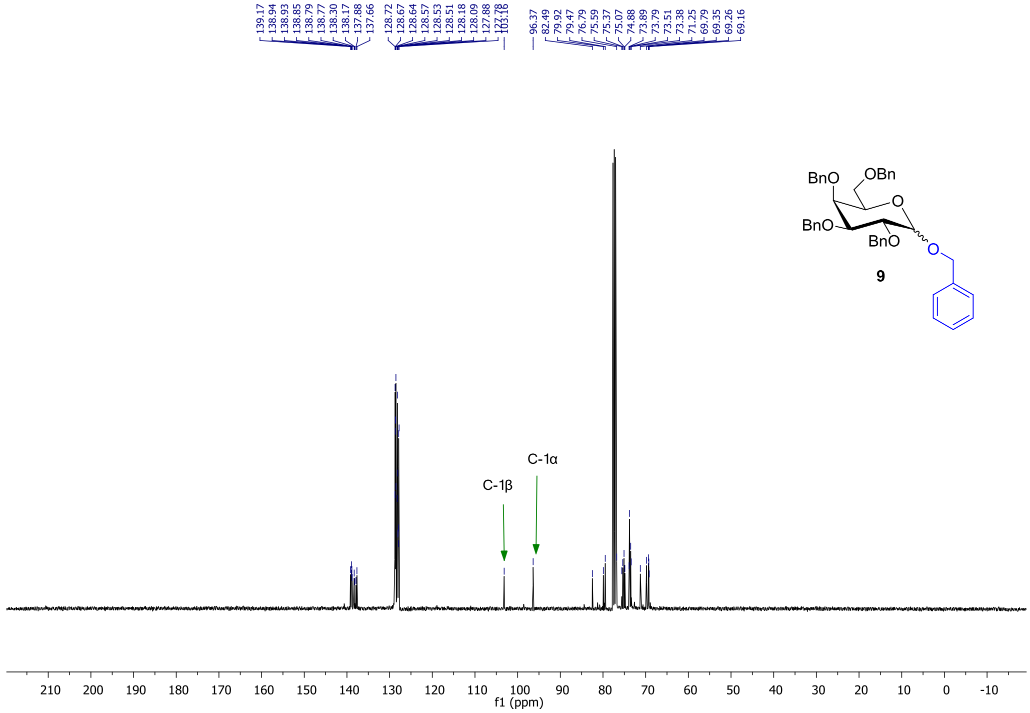

COSY spectrum of compound **9**

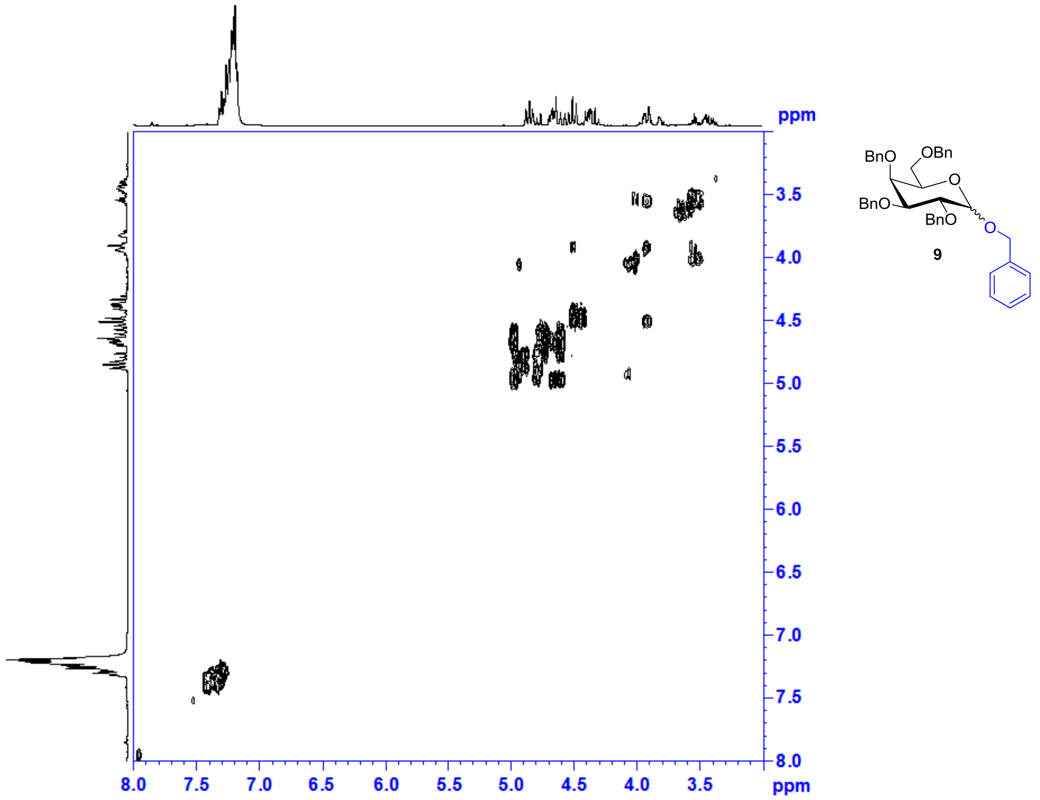

HSQC spectrum of compound **9**

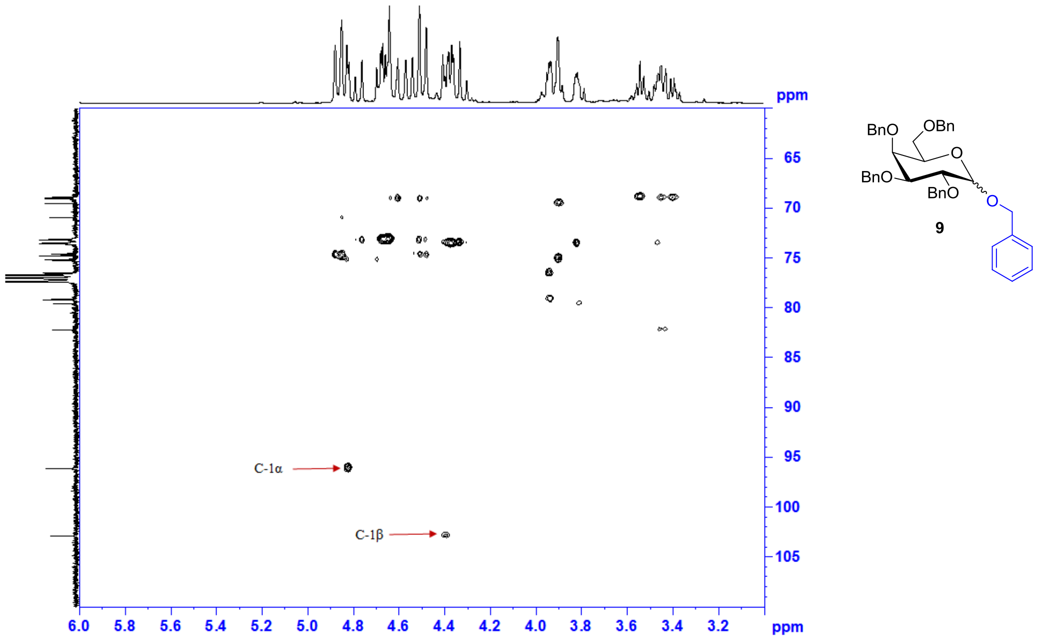

^{1}H NMR spectrum of compound **10** (400 MHz, $CDCl_3$)

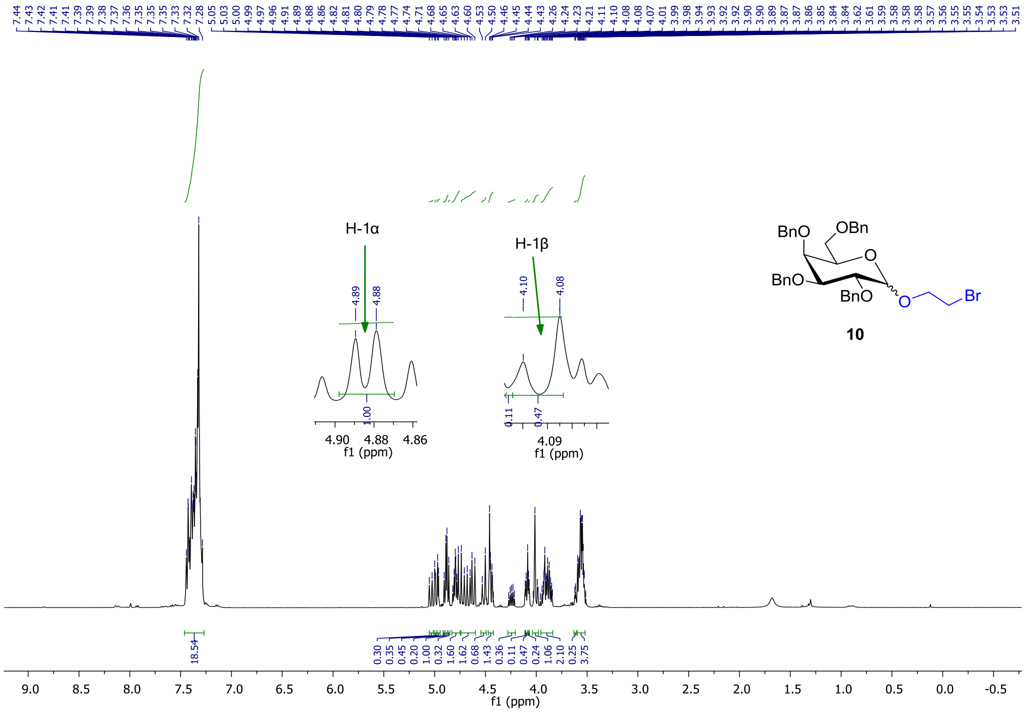

^{13}C NMR spectrum of compound **10** (100 MHz, $CDCl_3$)

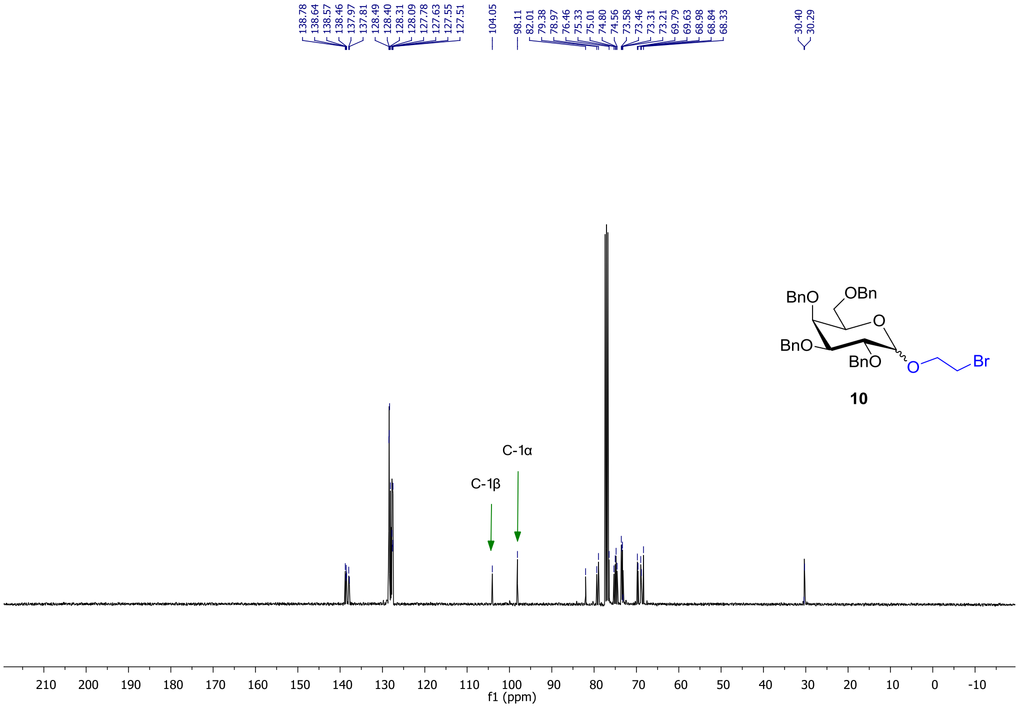

`COSY spectrum of compound **10**

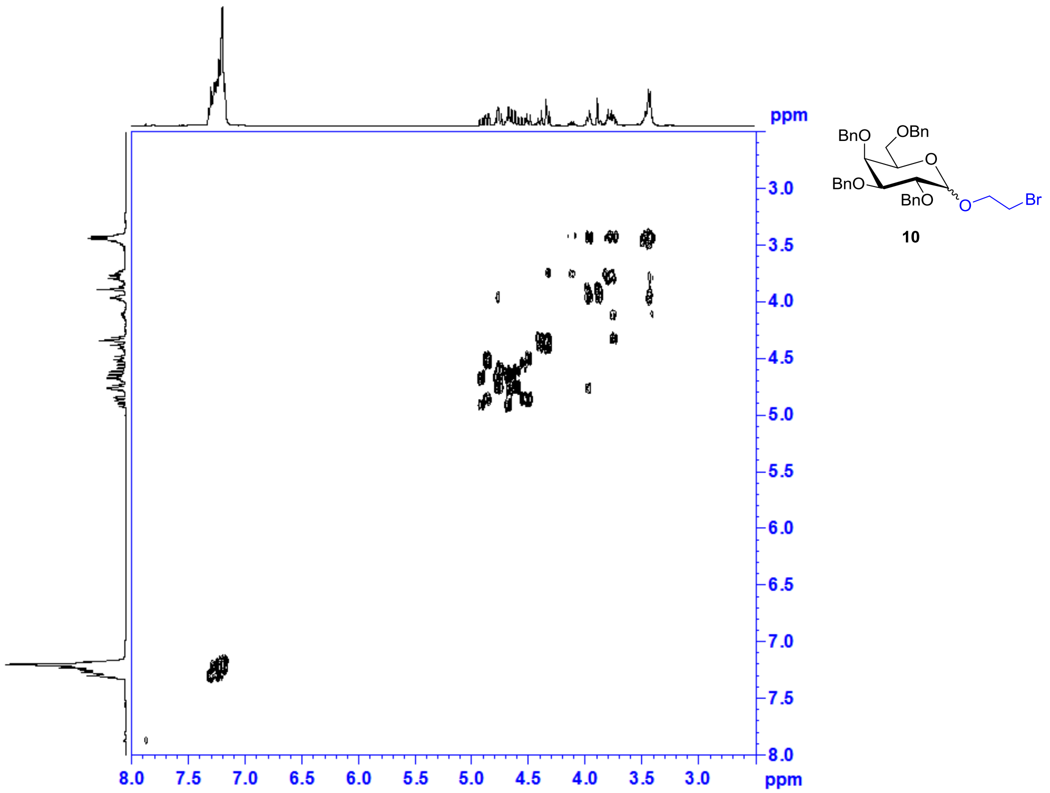

HSQC spectrum of compound **10**

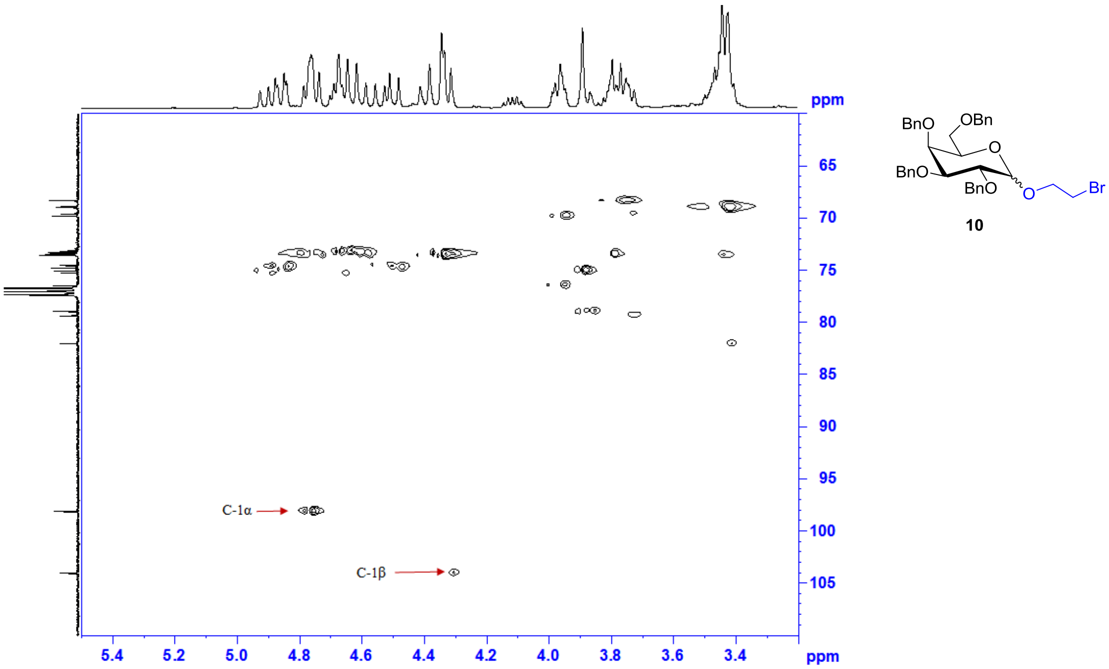

^{1}H NMR spectrum of compound **11** (400 MHz, $CDCl_3$)

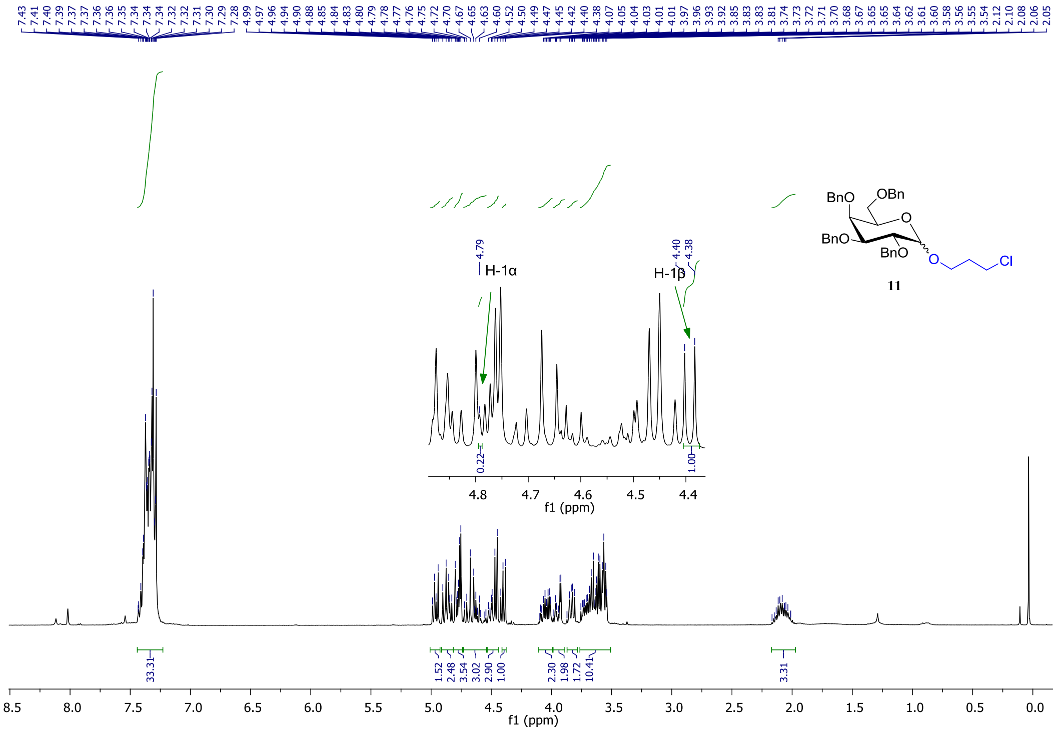

^{13}C NMR spectrum of compound **11** (100 MHz, $CDCl_3$)

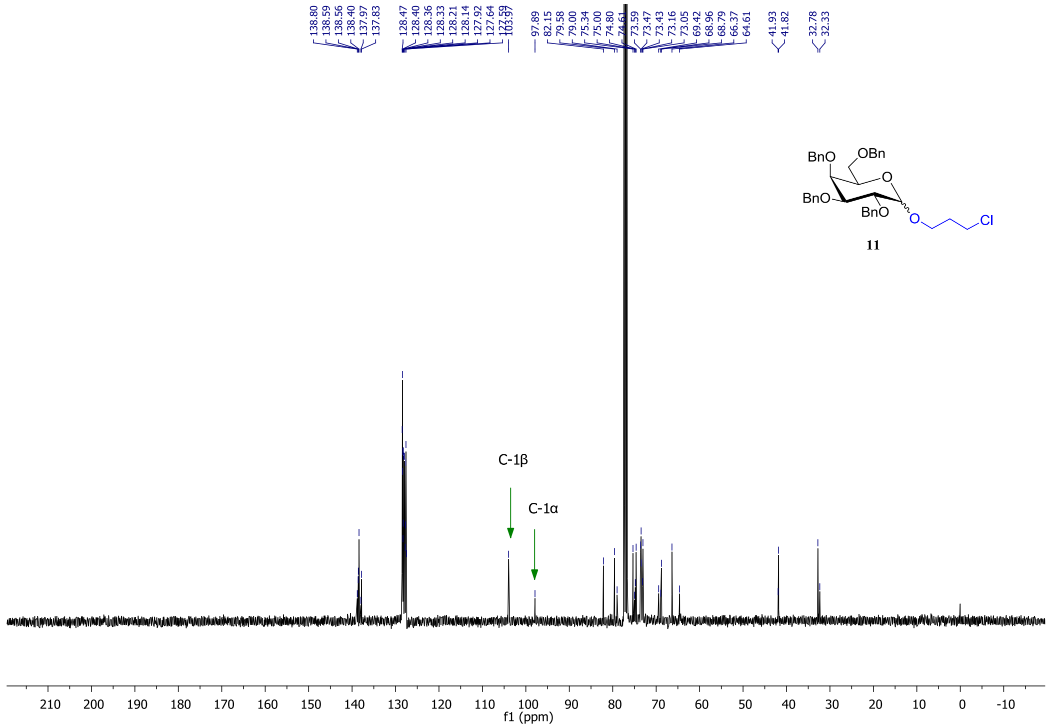

`COSY spectrum of compound **11**

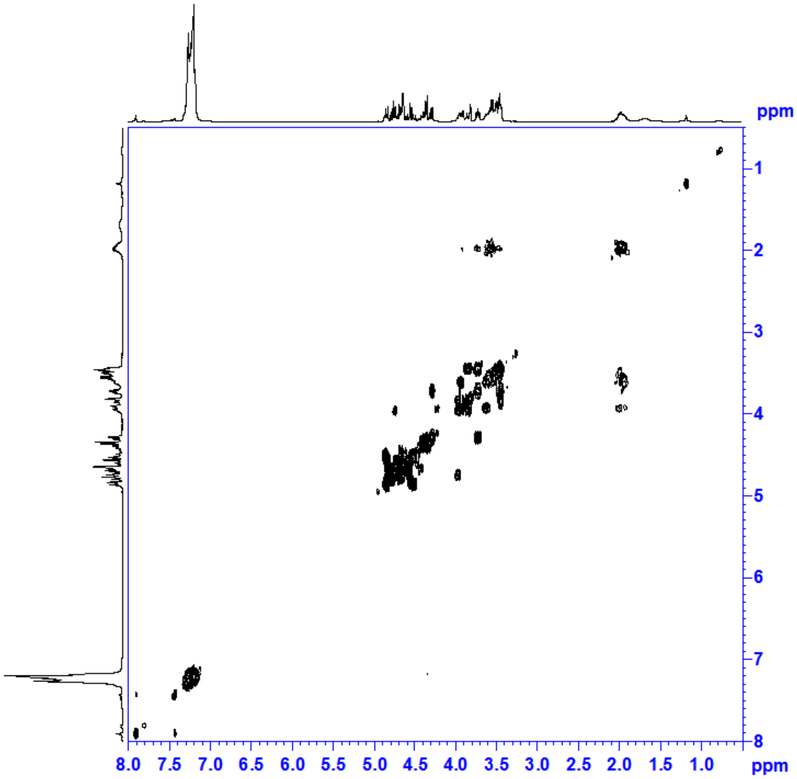

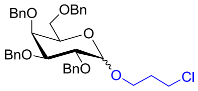

11

HSQC spectrum of compound **11**

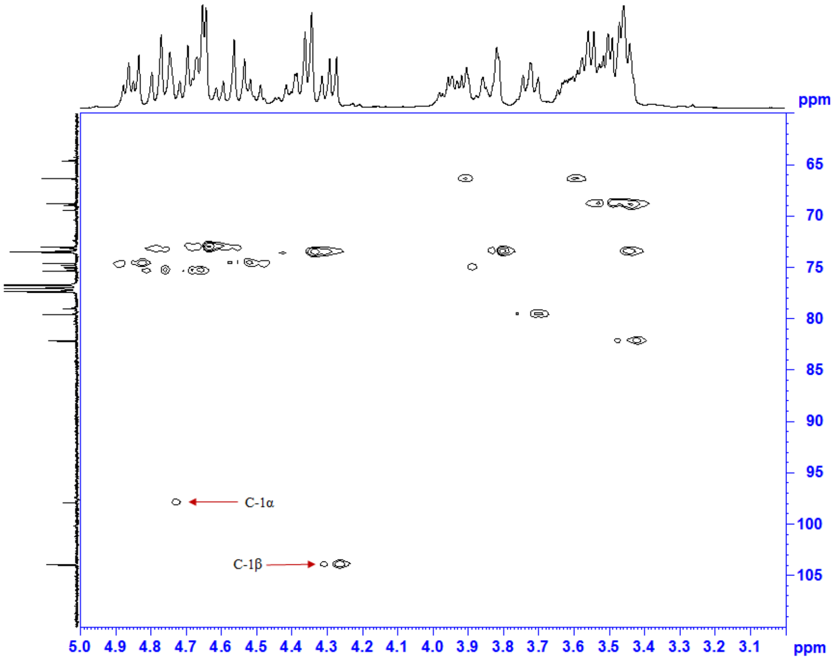

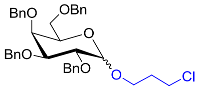

11

^{1}H NMR spectrum of compound **12** (400 MHz, $CDCl_3$)

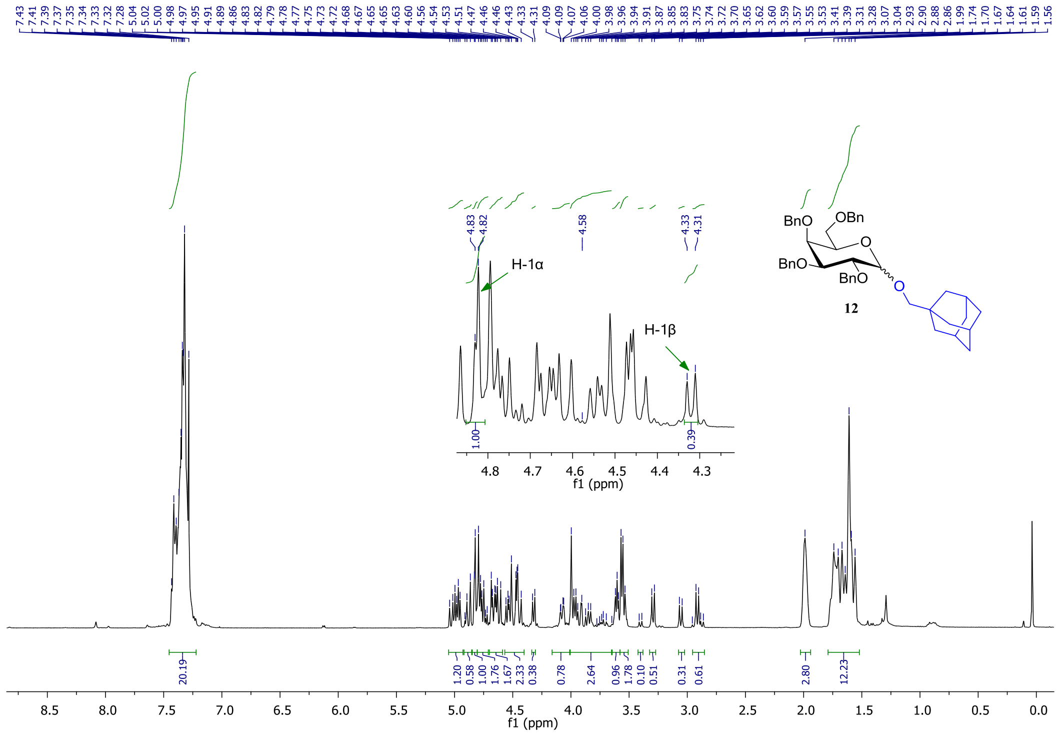

^{13}C NMR spectrum of compound **12** (100 MHz, $CDCl_3$)

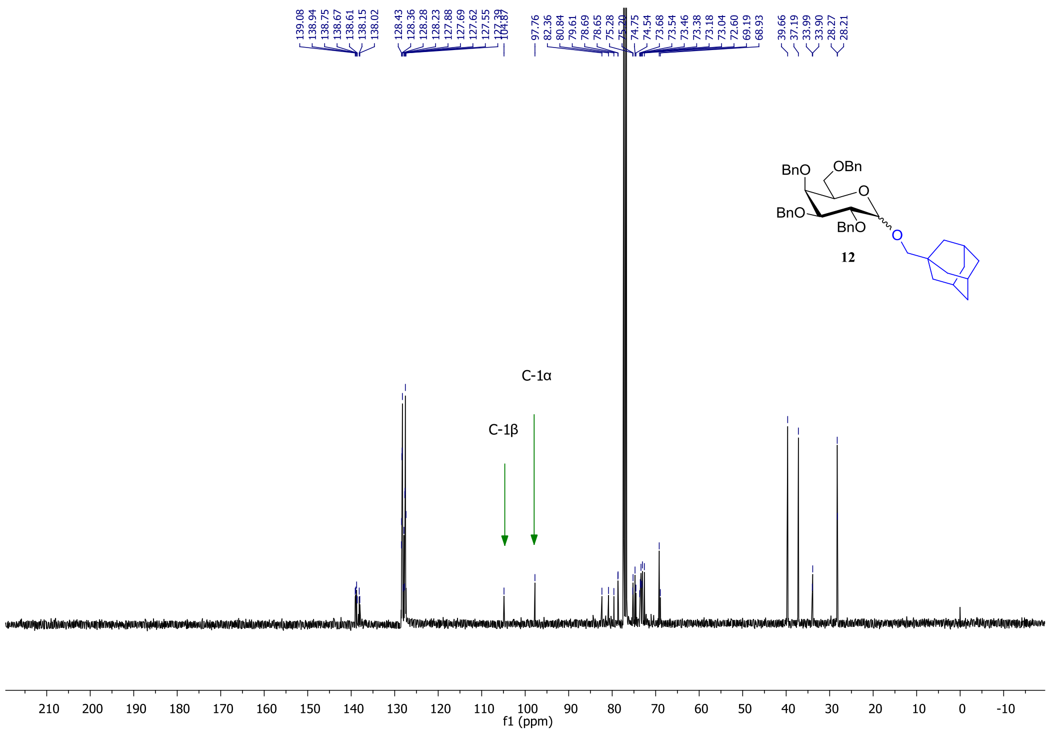

`COSY spectrum of compound **12**

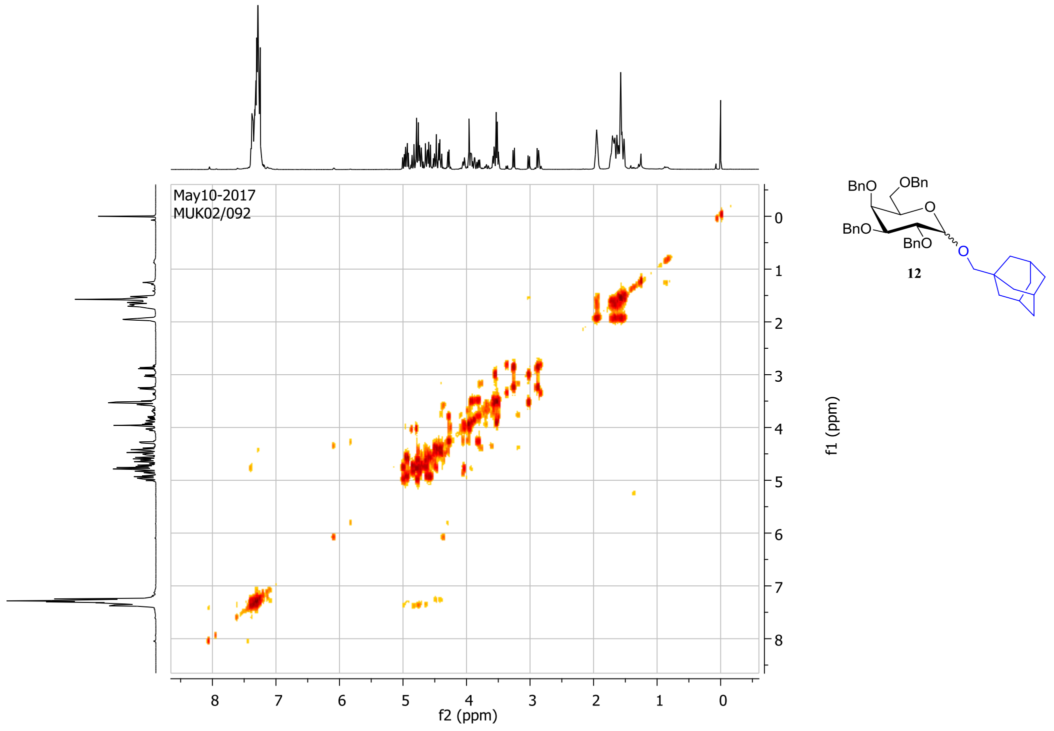

HSQC spectrum of compound **12**

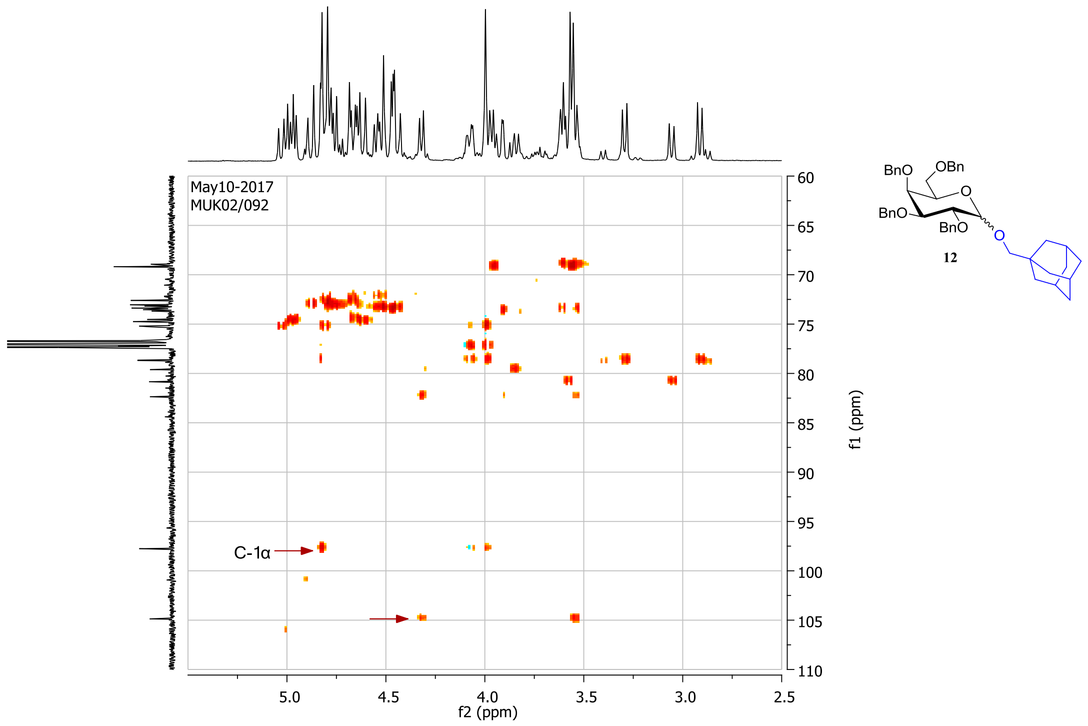

^{1}H NMR spectrum of compound **13** (400 MHz, $CDCl_3$)

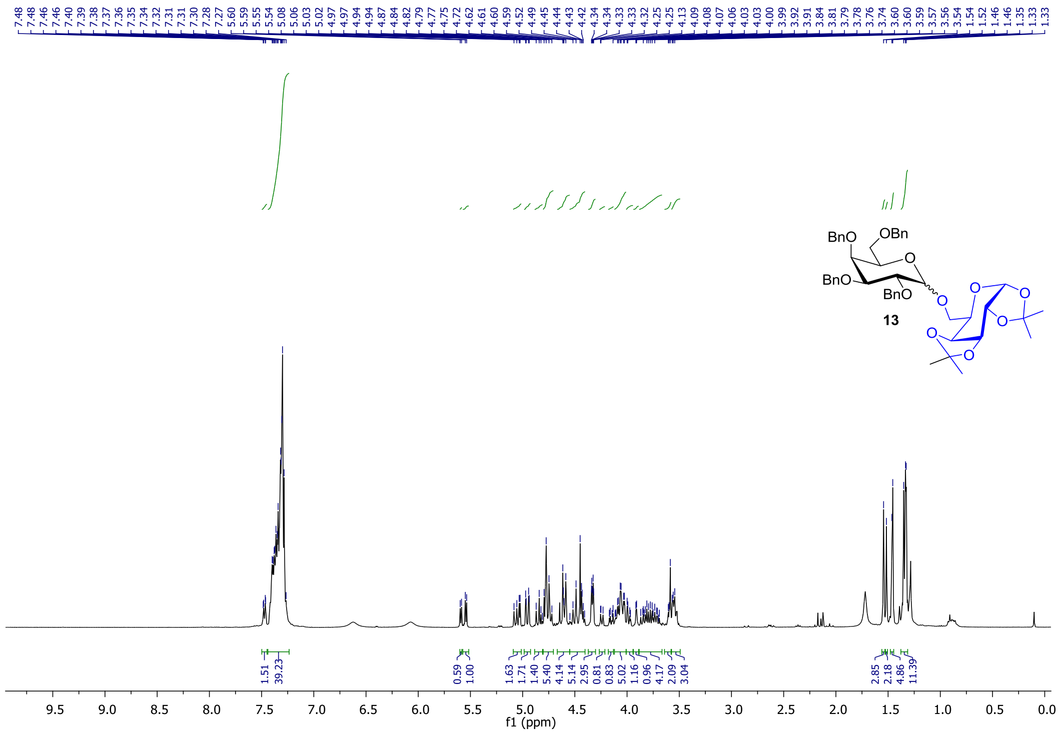

^{13}C NMR spectrum of compound **13** (100 MHz, $CDCl_3$)

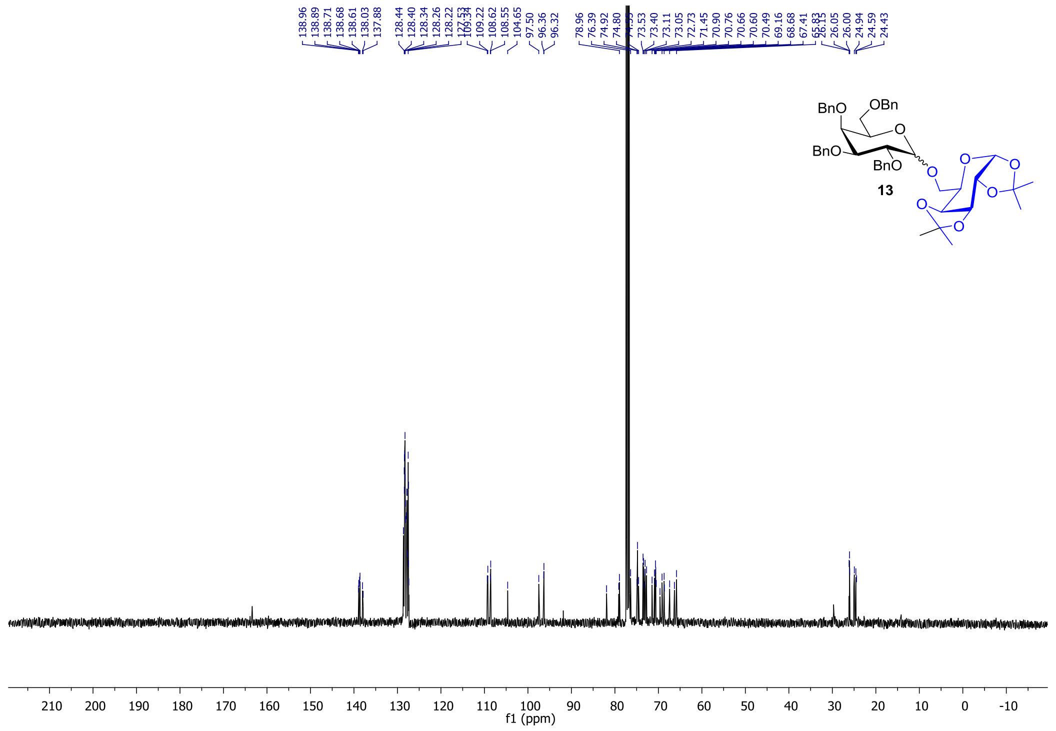

^{1}H NMR spectrum of compound **14** (400 MHz, $CDCl_3$)

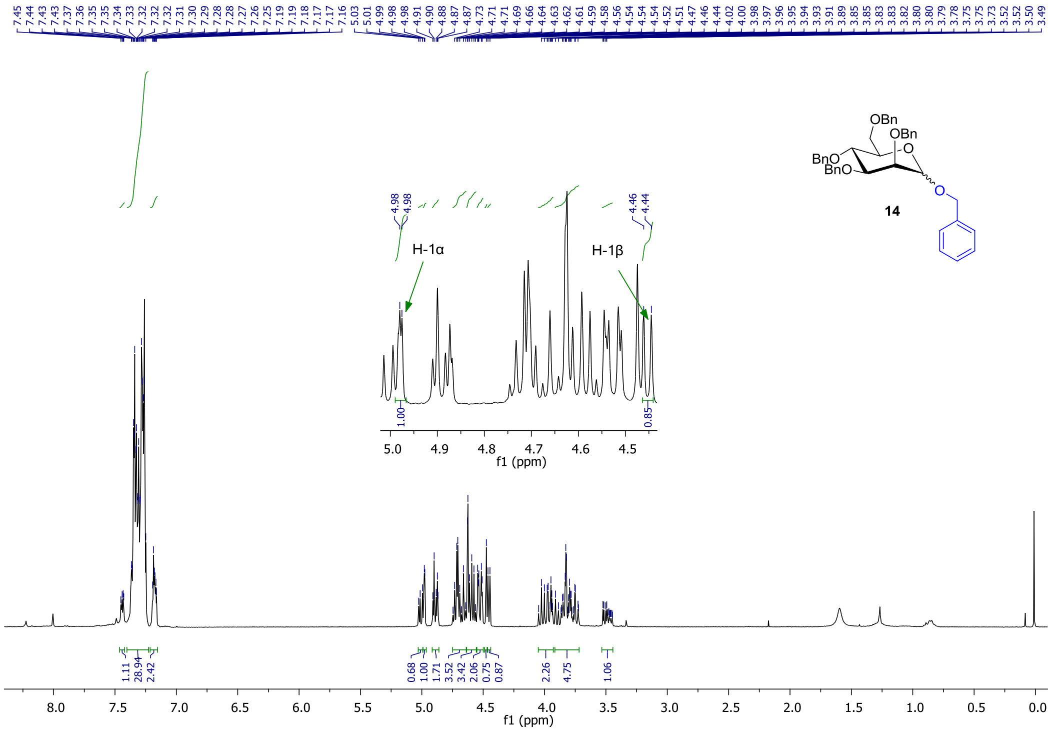

^{13}C NMR spectrum of compound **14** (100 MHz, $CDCl_3$)

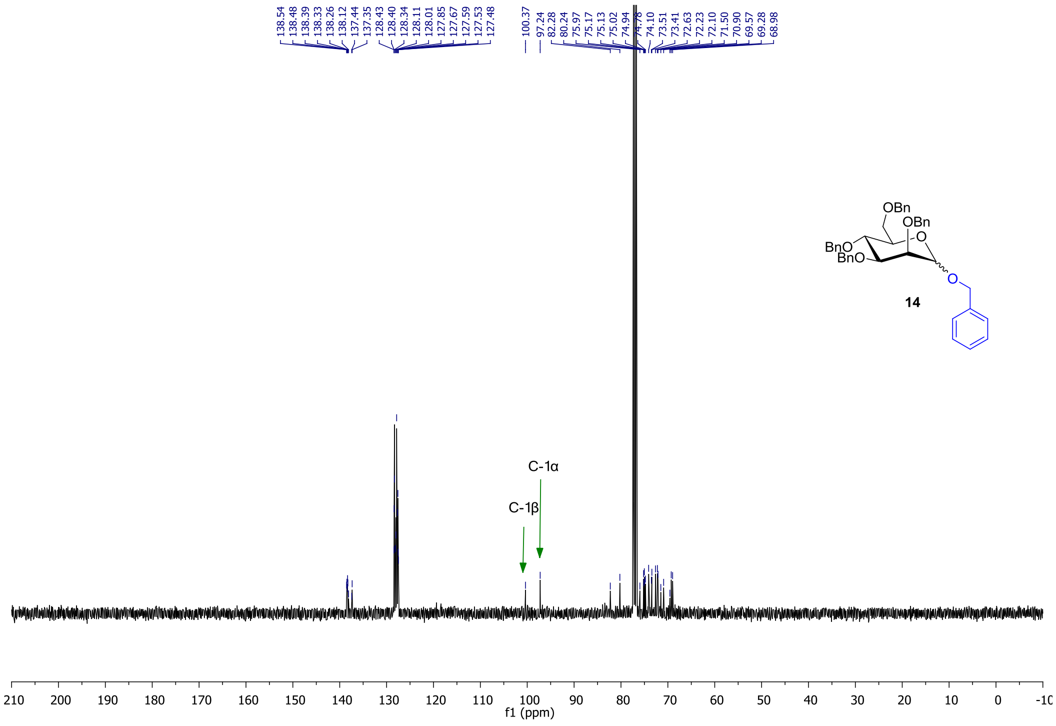

COSY spectrum of compound **14**

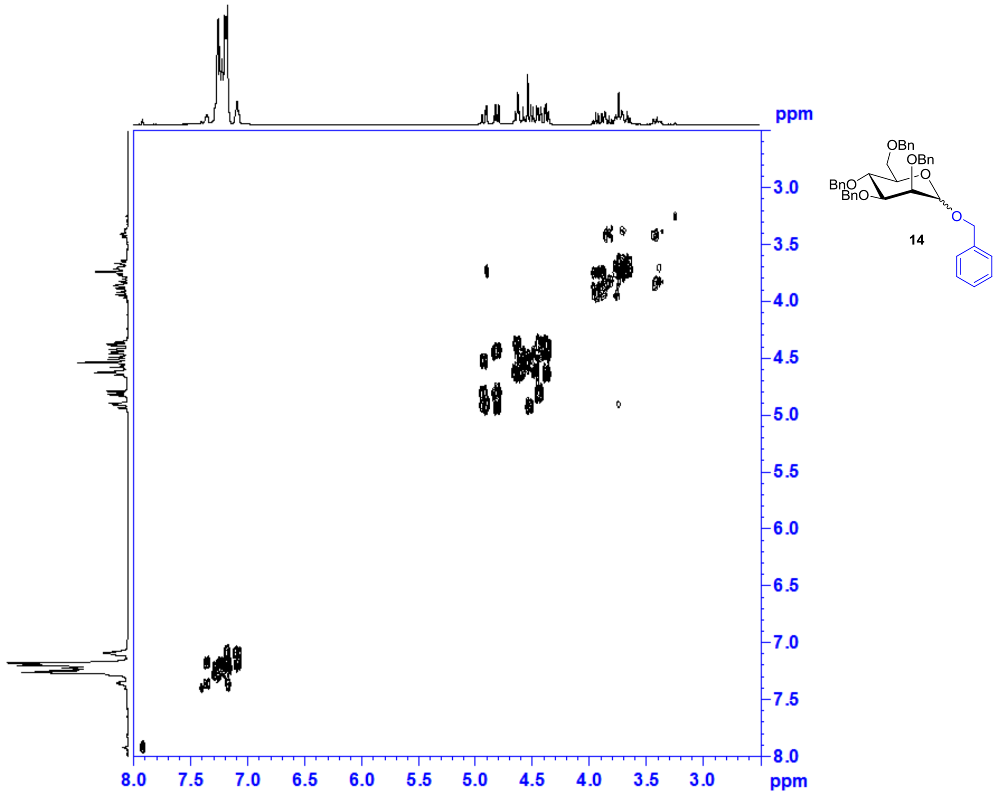

HSQC spectrum of compound **14**

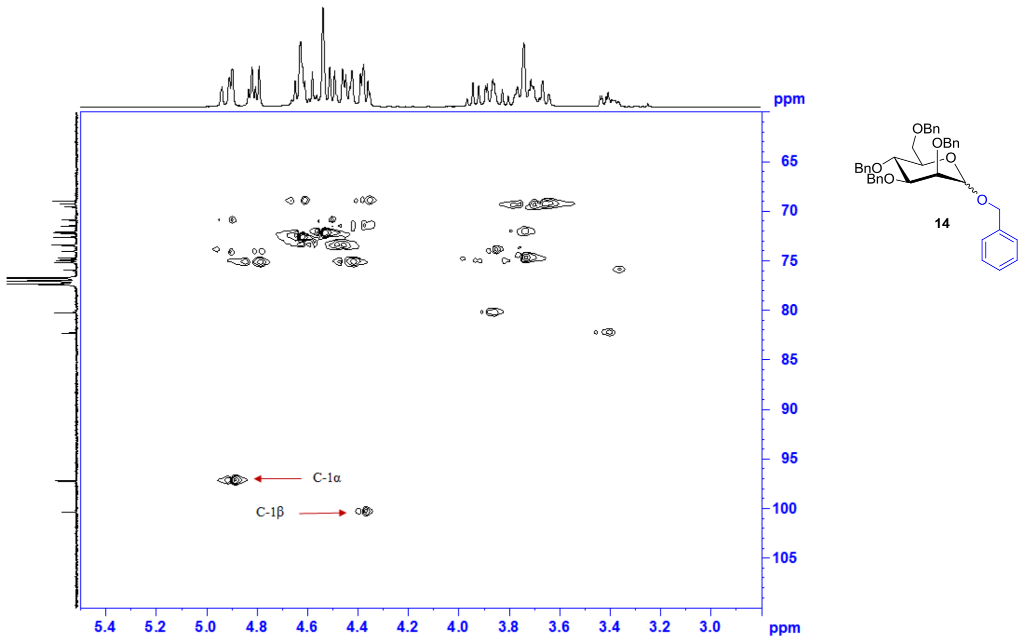

^{1}H NMR spectrum of compound **15** (400 MHz, $CDCl_3$)

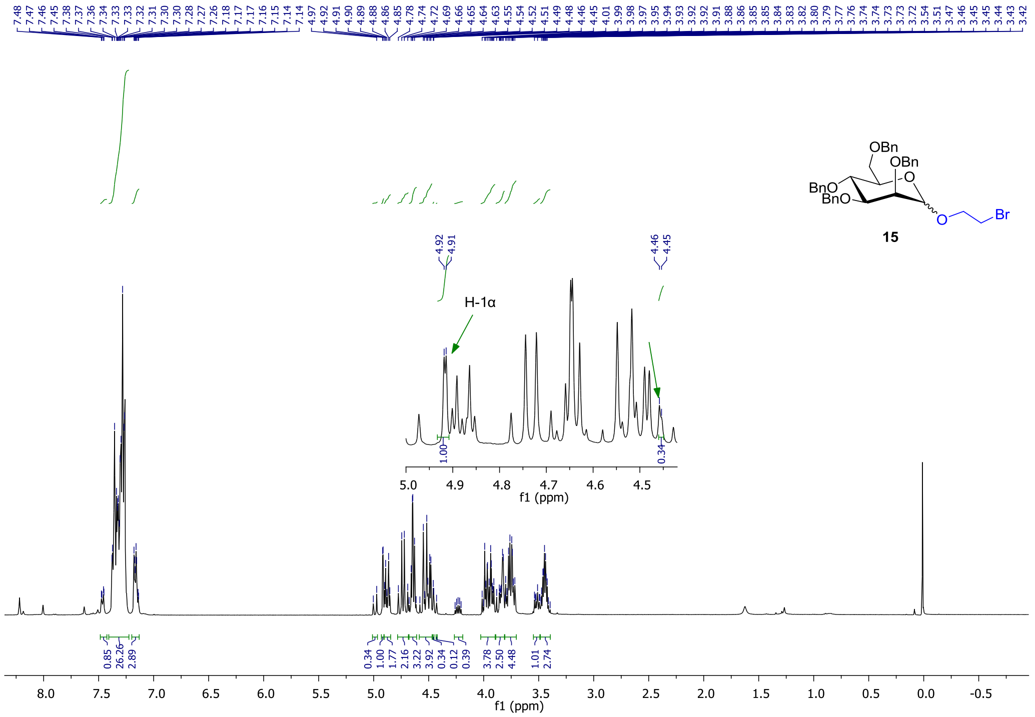

^{13}C NMR spectrum of compound **15** (100 MHz, $CDCl_3$)

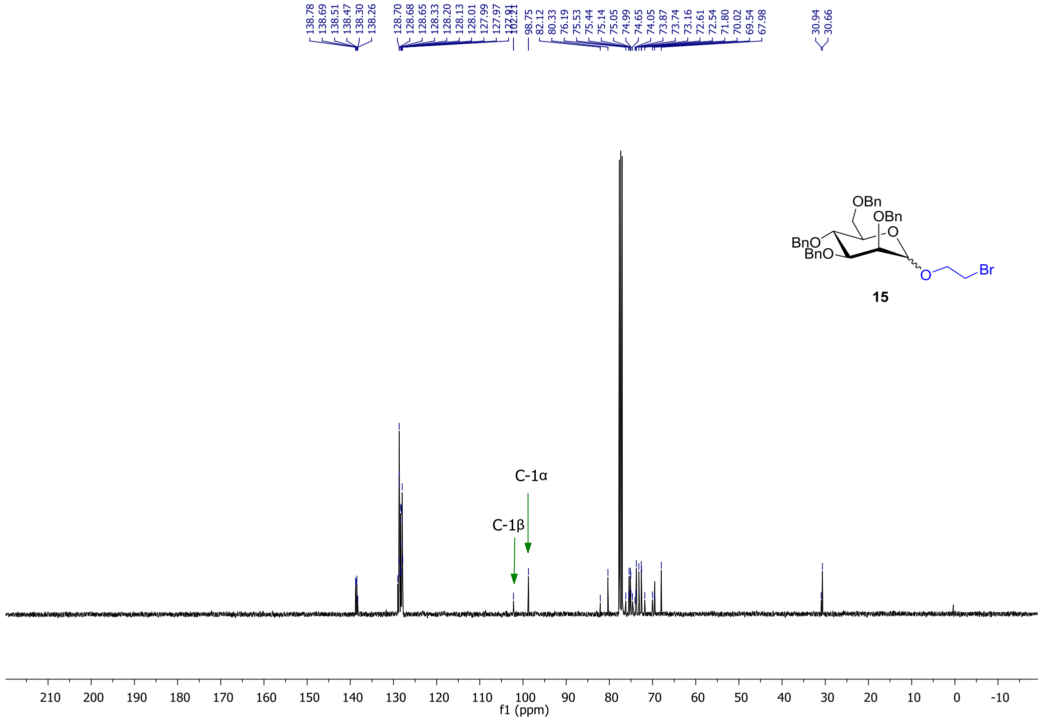

COSY spectrum of compound **15**

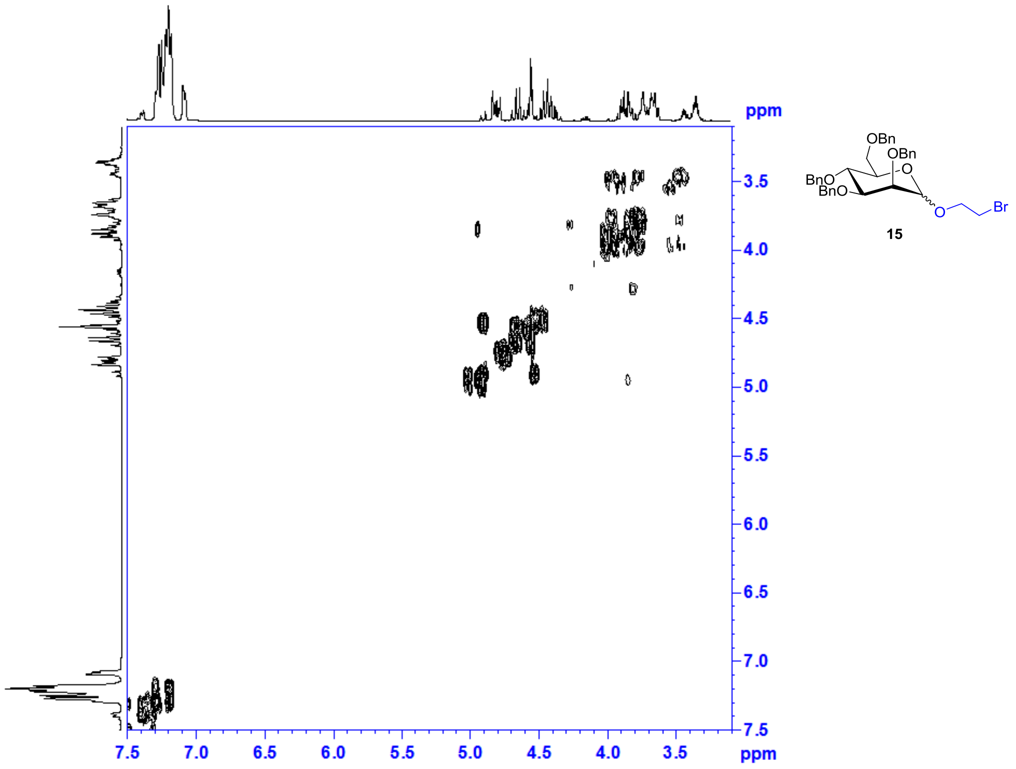

HSQC spectrum of compound **15**

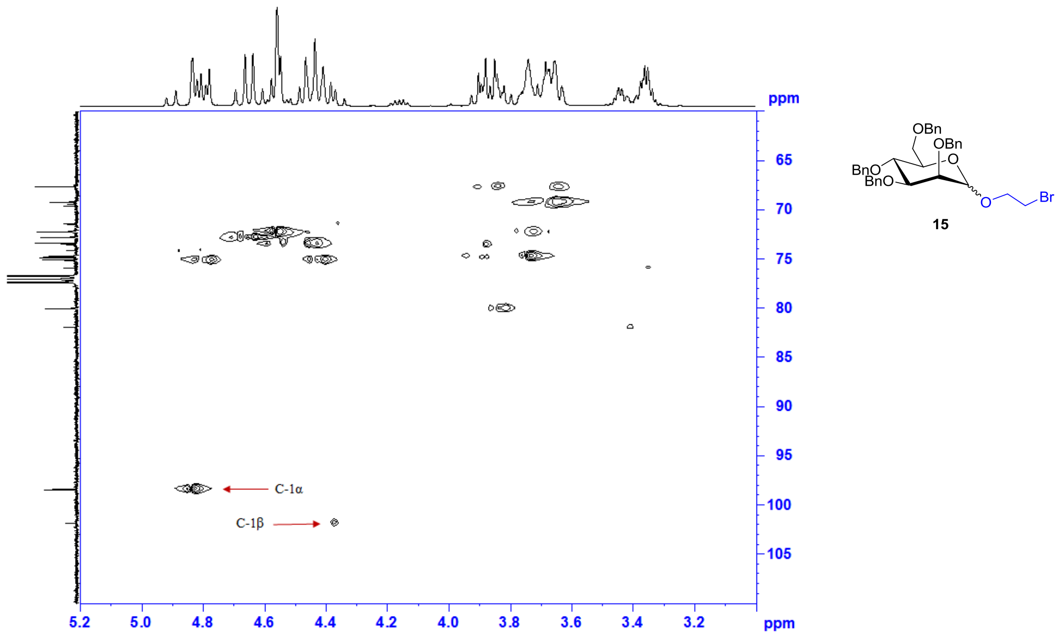

^{1}H NMR spectrum of compound **16** (400 MHz, $CDCl_3$)

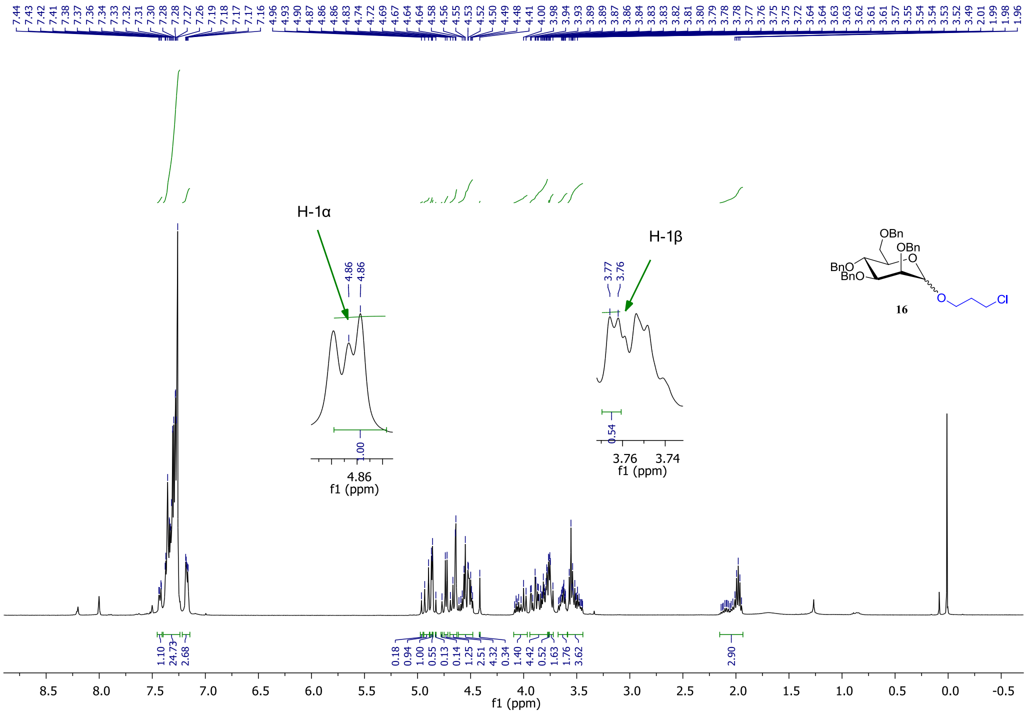

^{13}C NMR spectrum of compound **16** (100 MHz, $CDCl_3$)

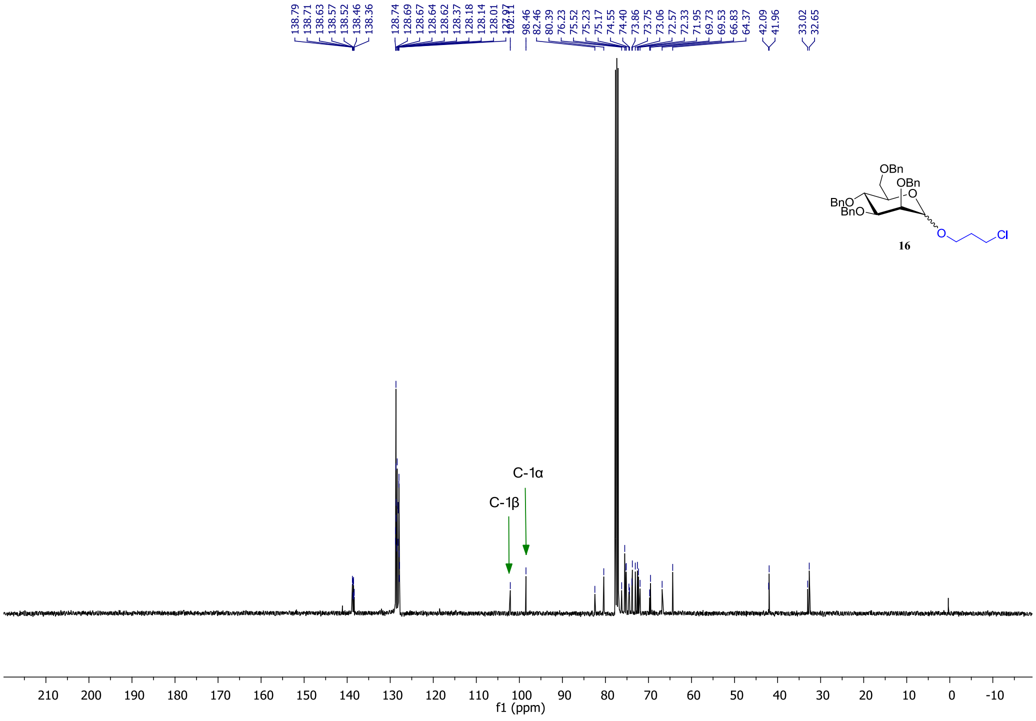

COSY spectrum of compound **16**

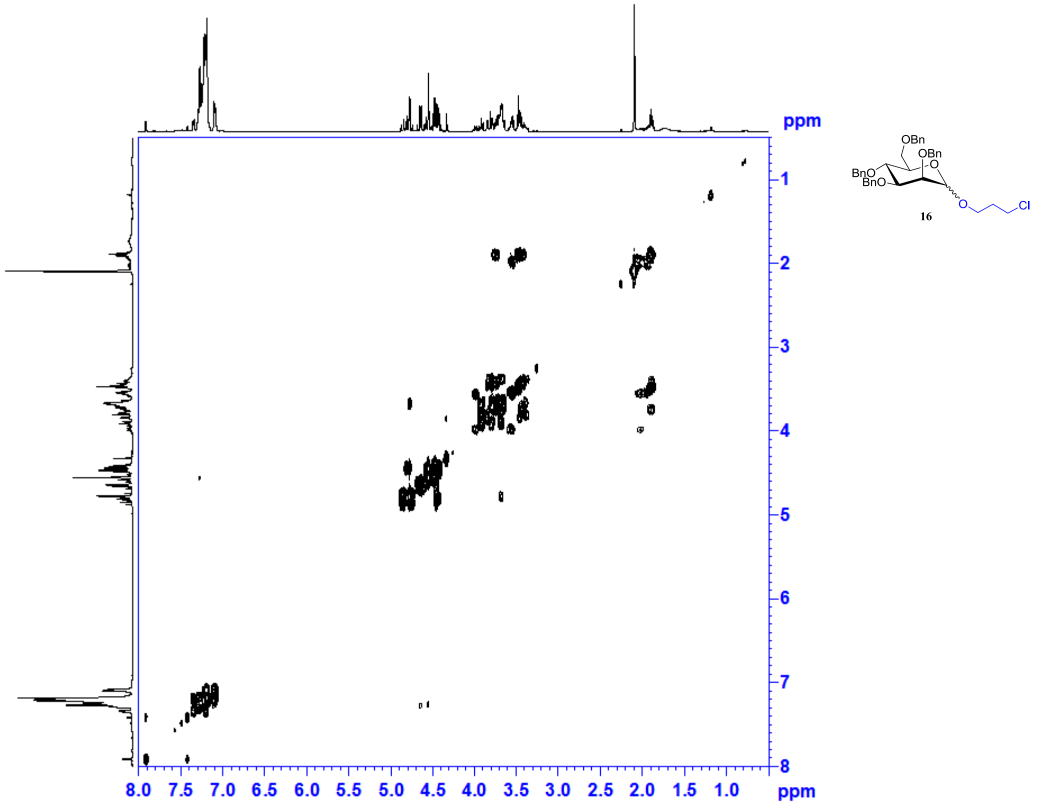

HSQC spectrum of compound **16**

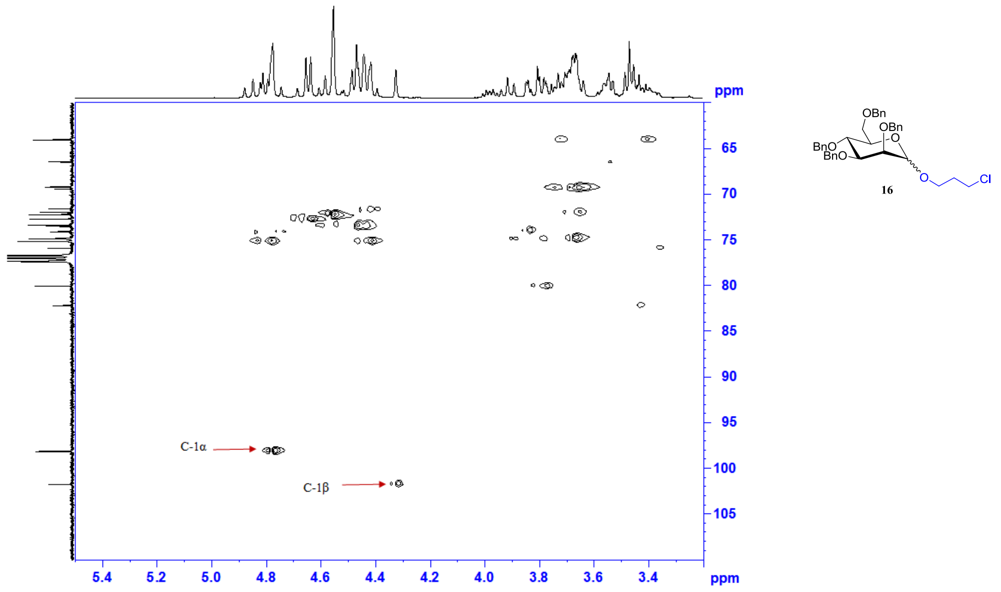

;

^{1}H NMR spectrum of compound **17** (400 MHz, $CDCl_3$)

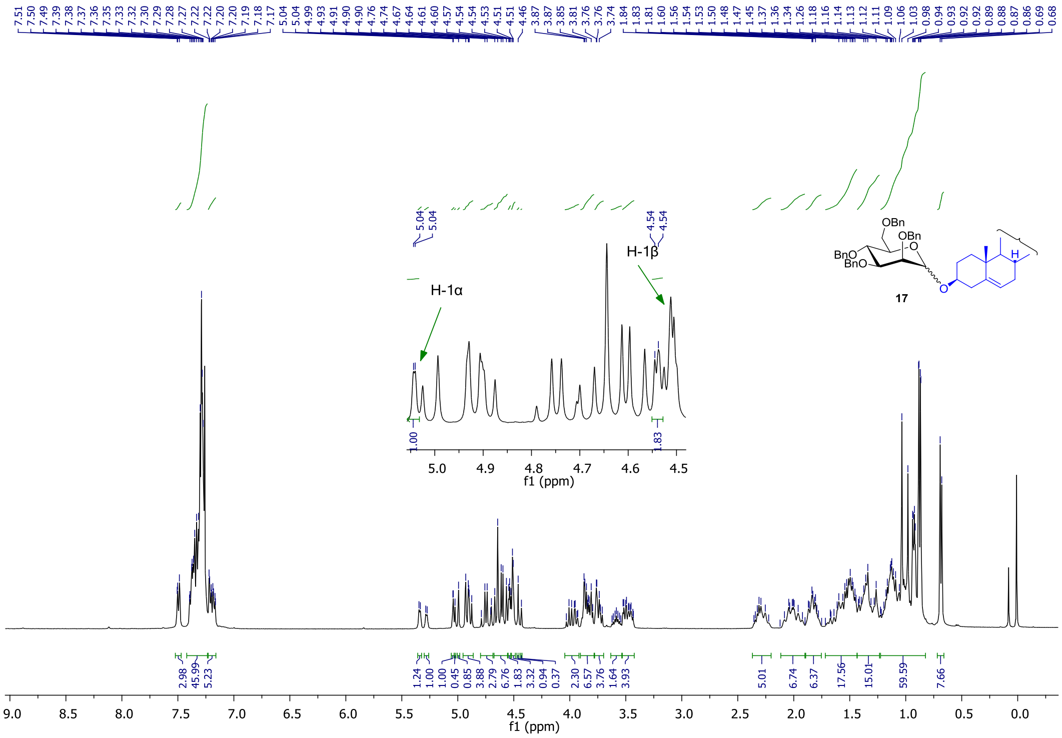

^{13}C NMR spectrum of compound **17** (100 MHz, $CDCl_3$)

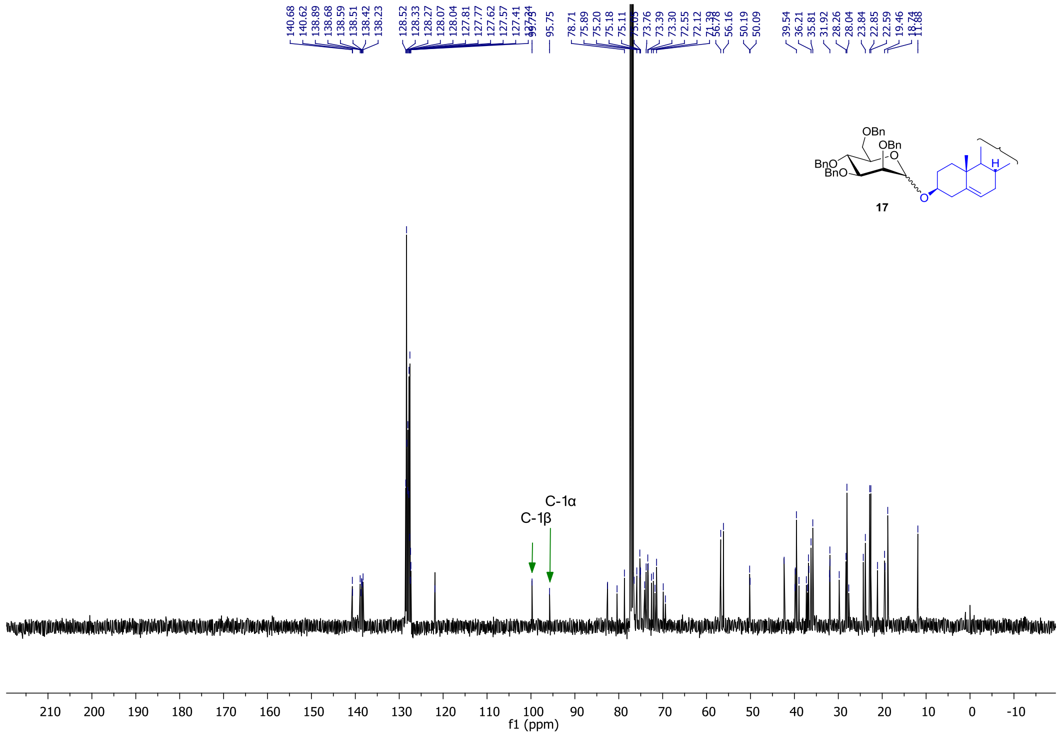

COSY spectrum of compound **17**

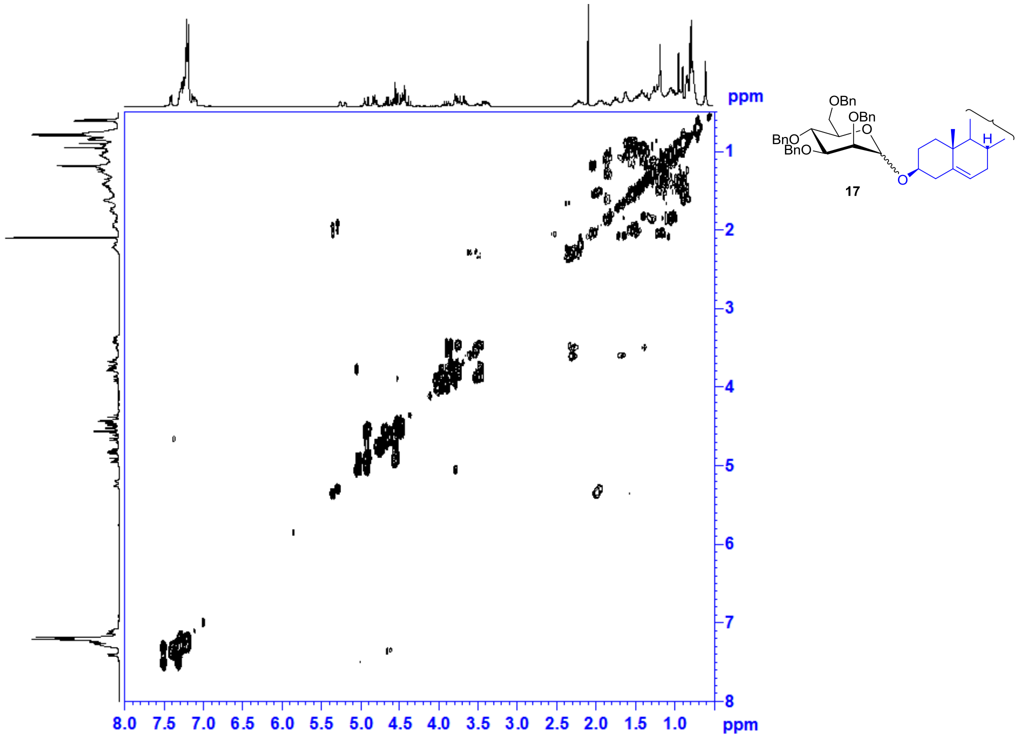

HSQC spectrum of compound **17**

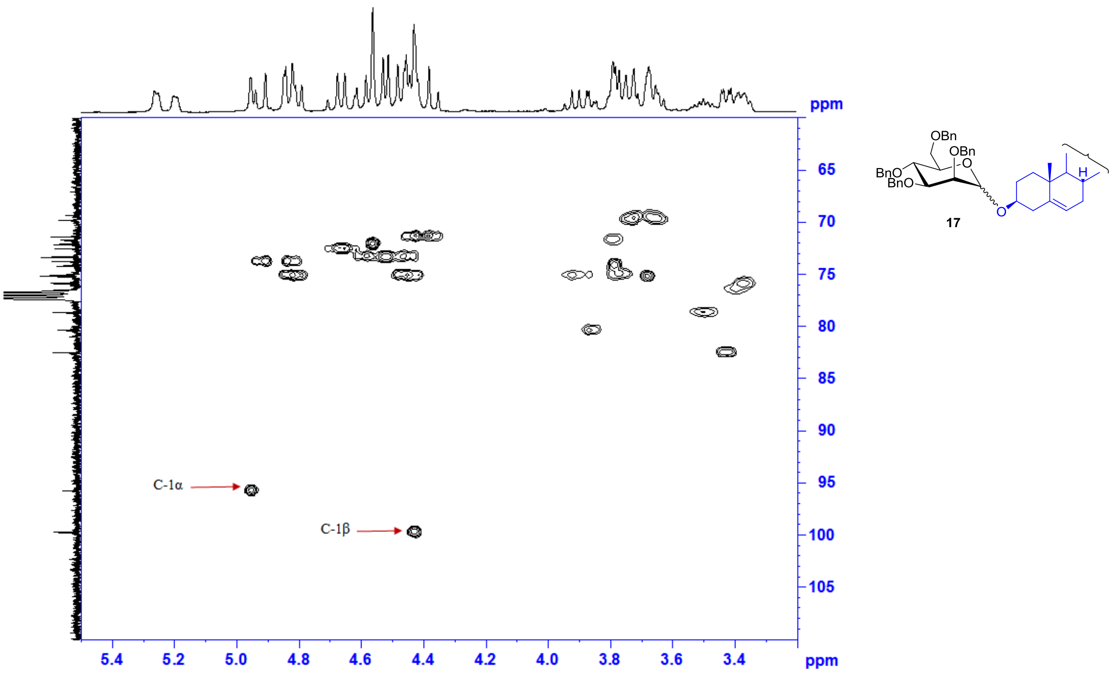

^{1}H NMR spectrum of compound **18** (400 MHz, $CDCl_3$)

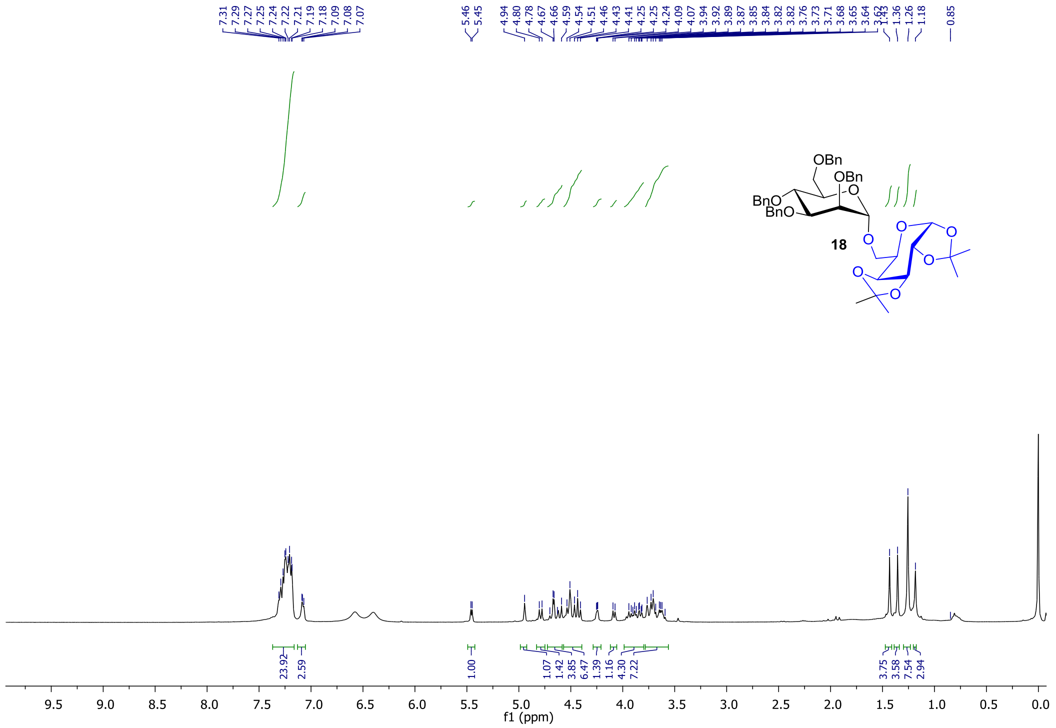

^{13}C NMR spectrum of compound **18** (100 MHz, $CDCl_3$)

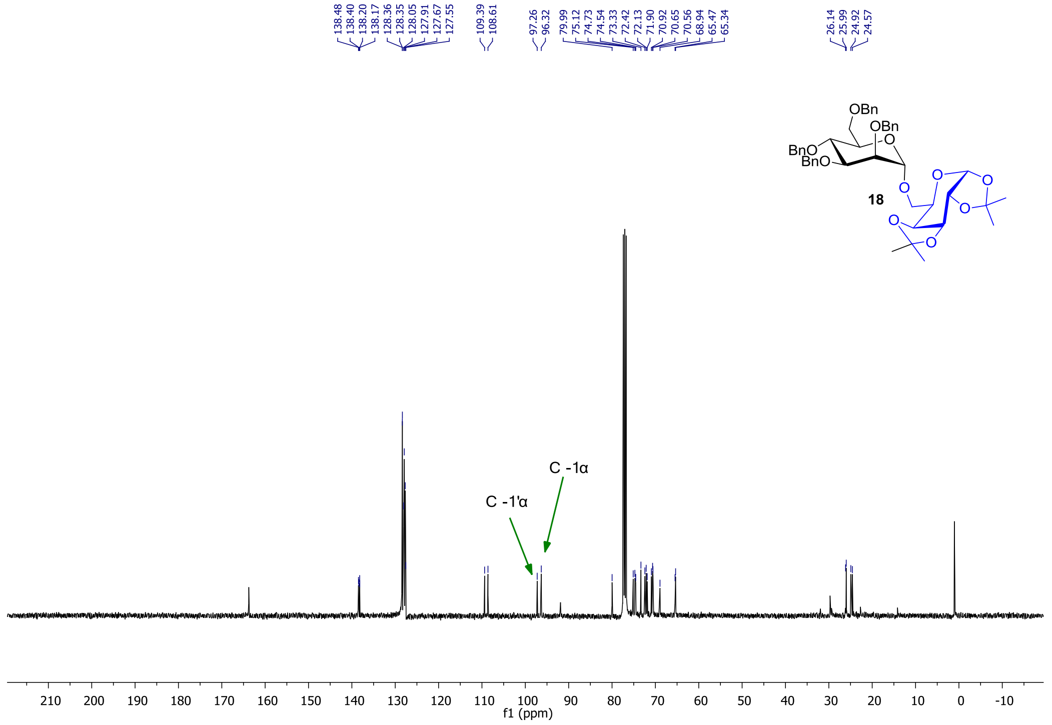

^{1}H NMR spectrum of compound **19** (400 MHz, $CDCl_3$)

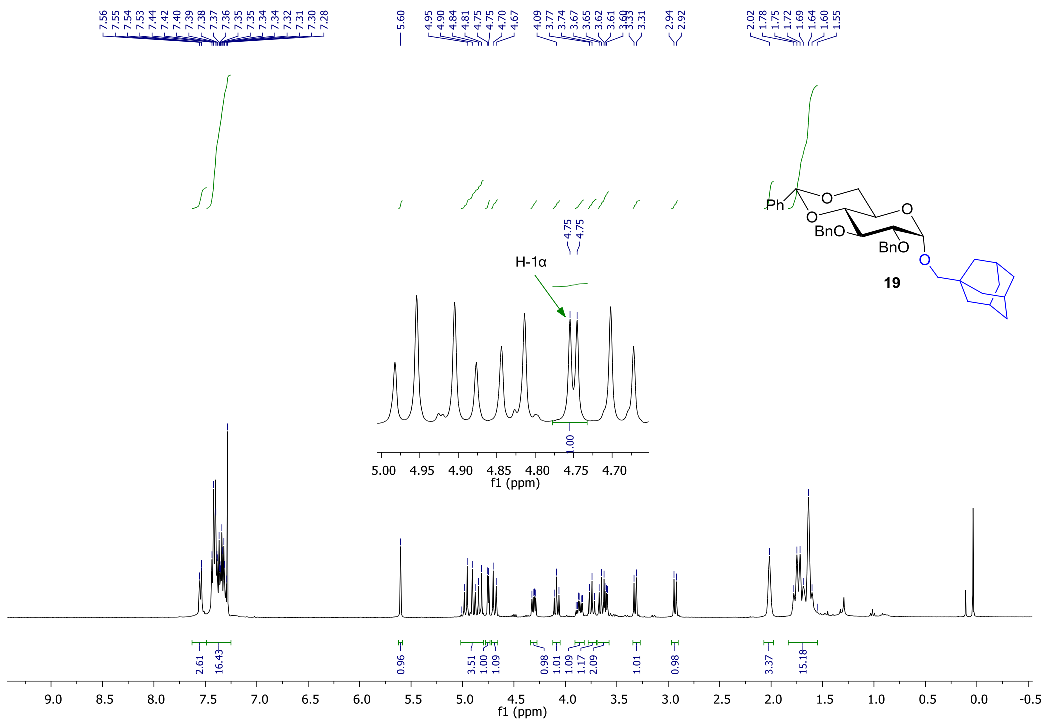

^{13}C NMR spectrum of compound **19** (100 MHz, $CDCl_3$)

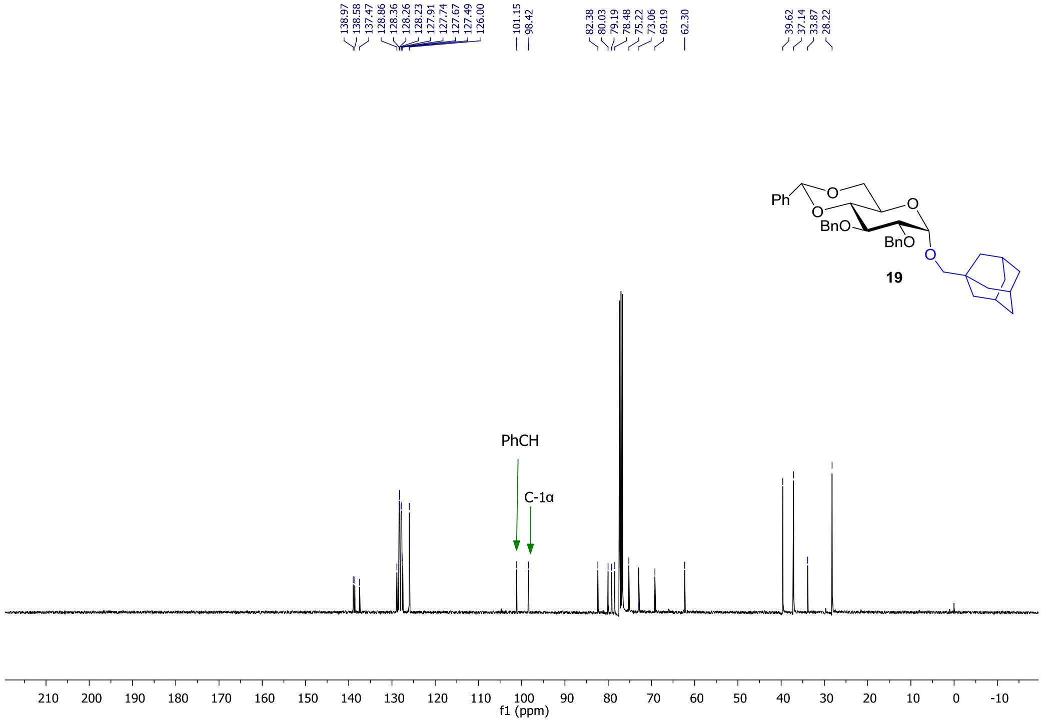

COSY spectrum of compound **19**

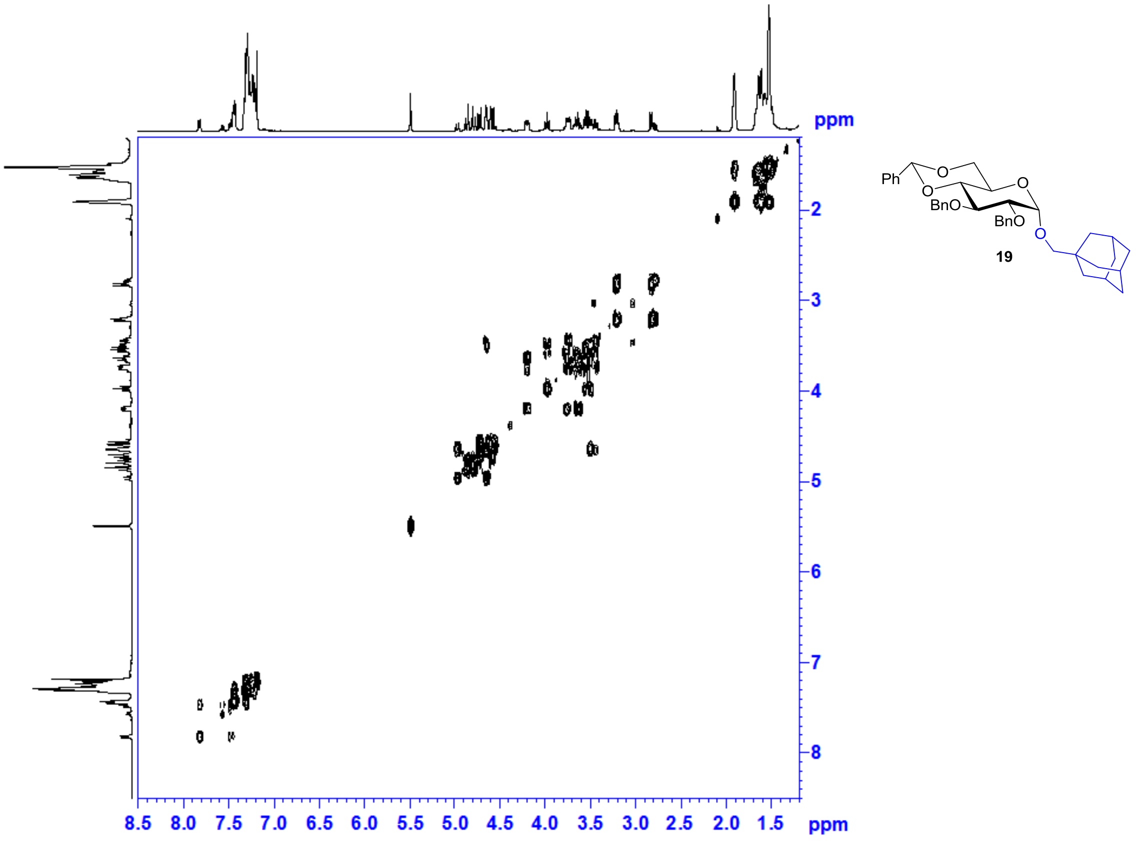

HSQC spectrum of compound **19**

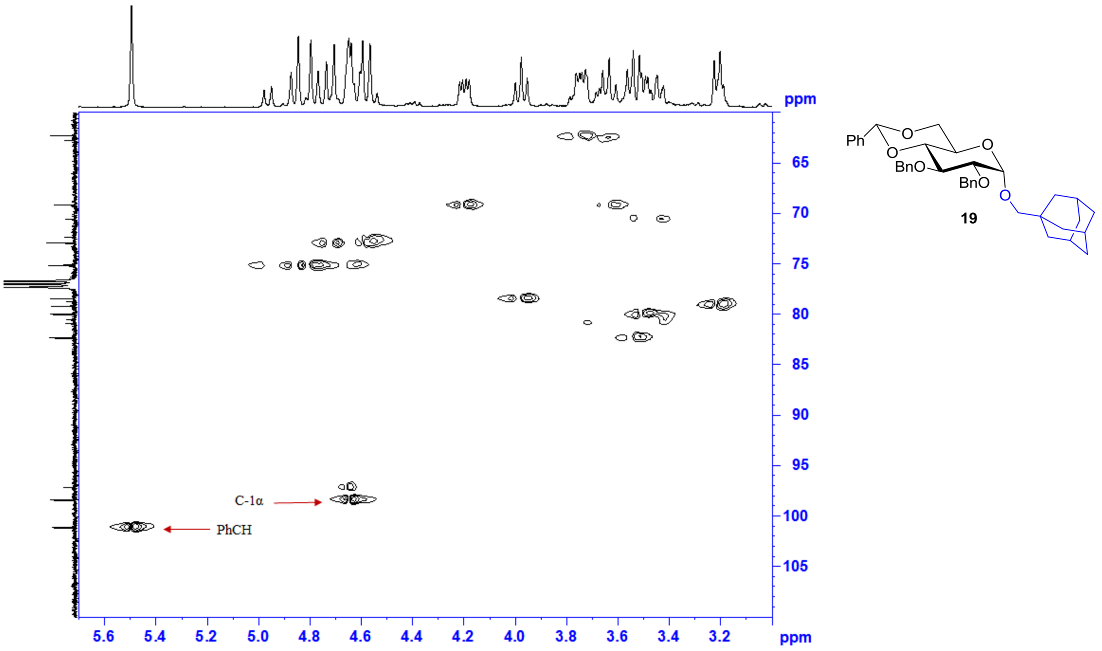

^{1}H NMR spectrum of compound **20** (400 MHz, $CDCl_3$)

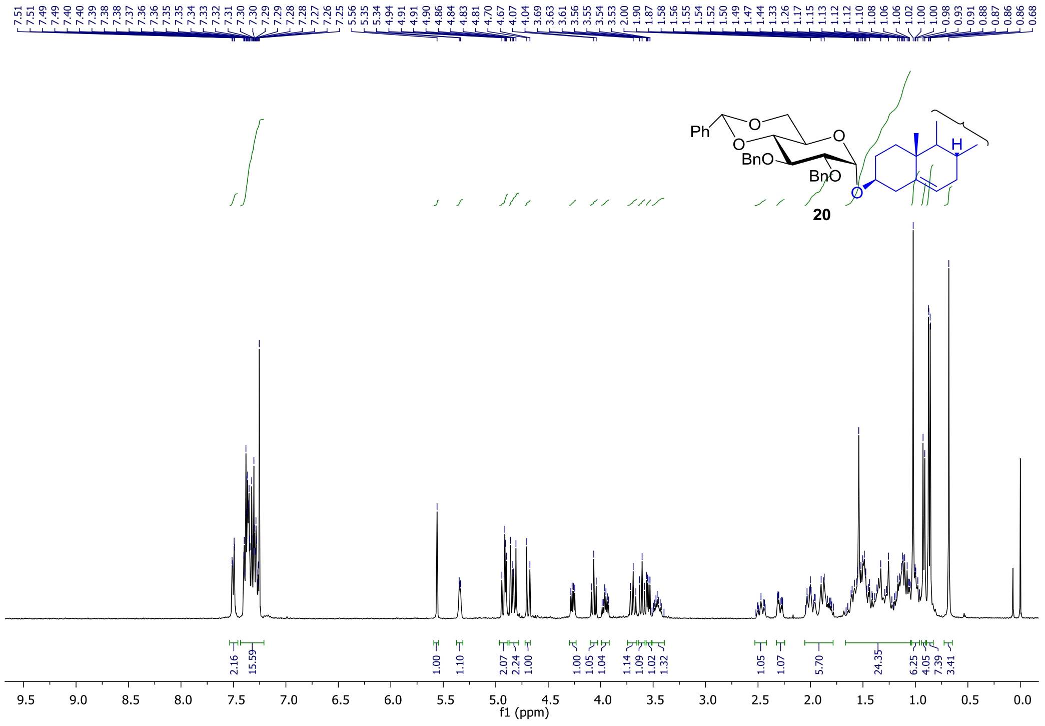

^{13}C NMR spectrum of compound **20** (100 MHz, $CDCl_3$)

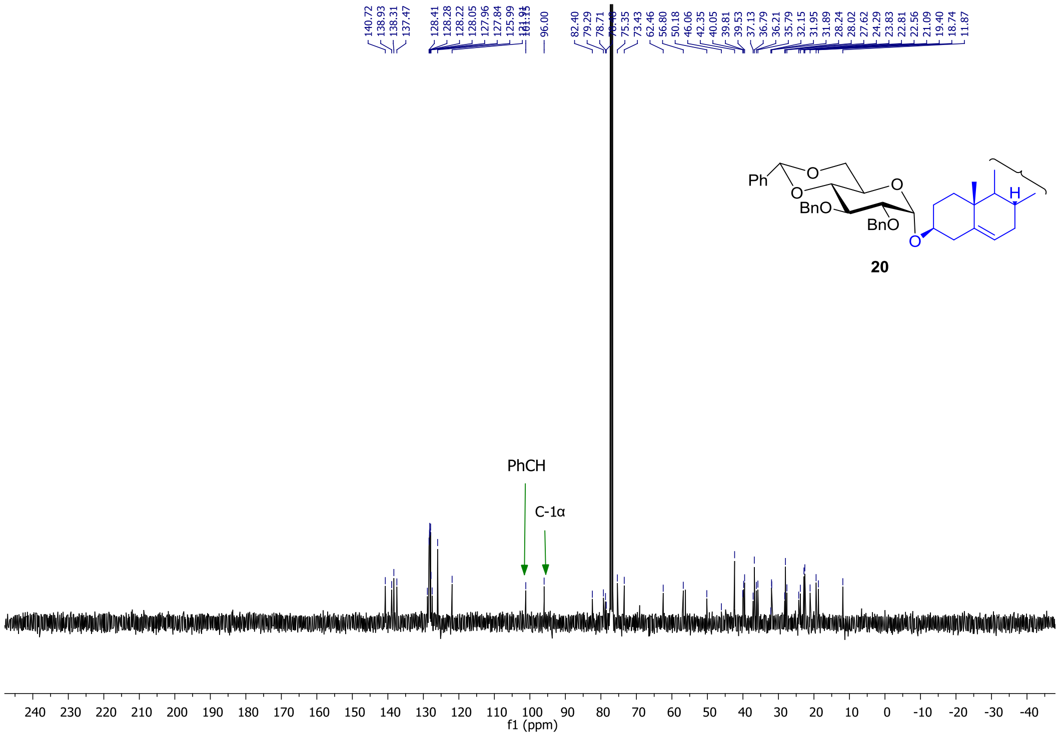

COSY spectrum of compound **20**

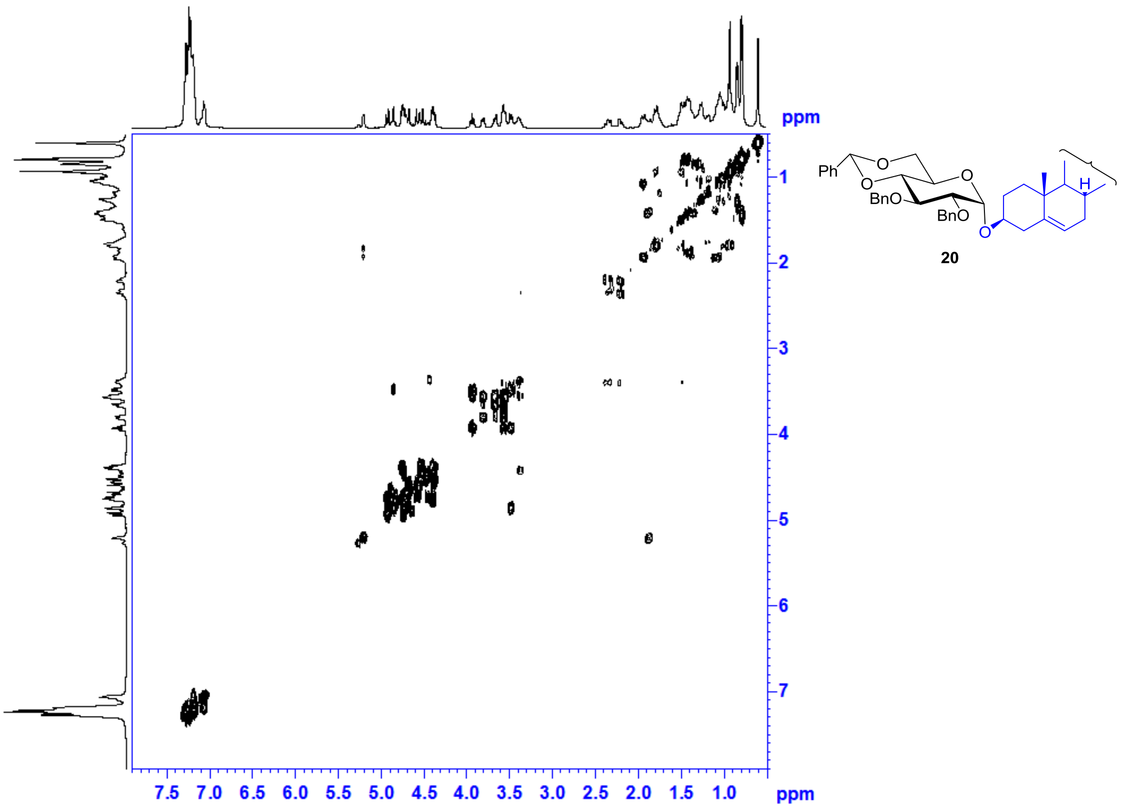

HSQC spectrum of compound **20**

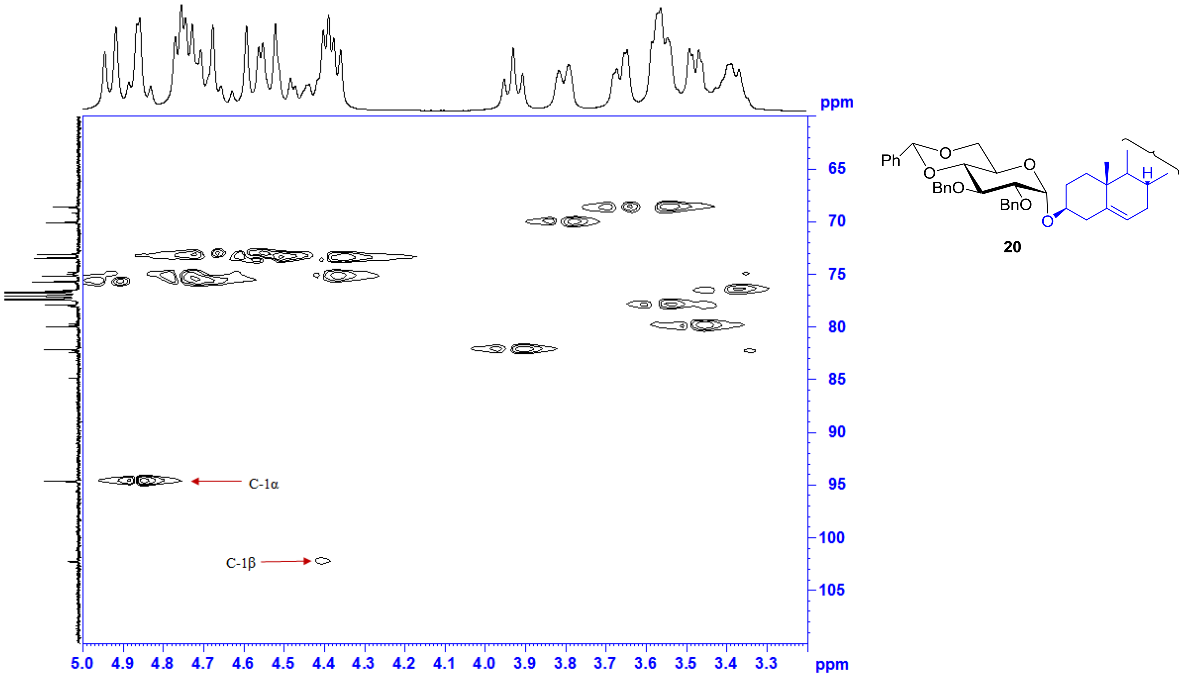

^{1}H NMR spectrum of compound **21** (400 MHz, $CDCl_3$)

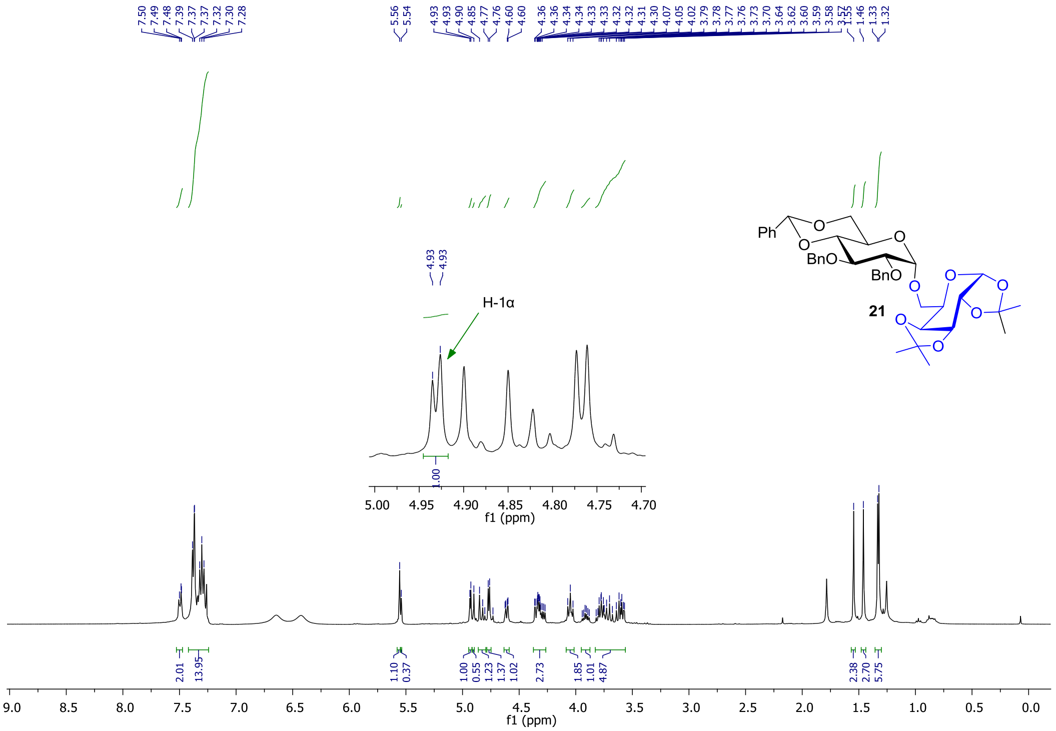

^{13}C NMR spectrum of compound **21** (100 MHz, $CDCl_3$)

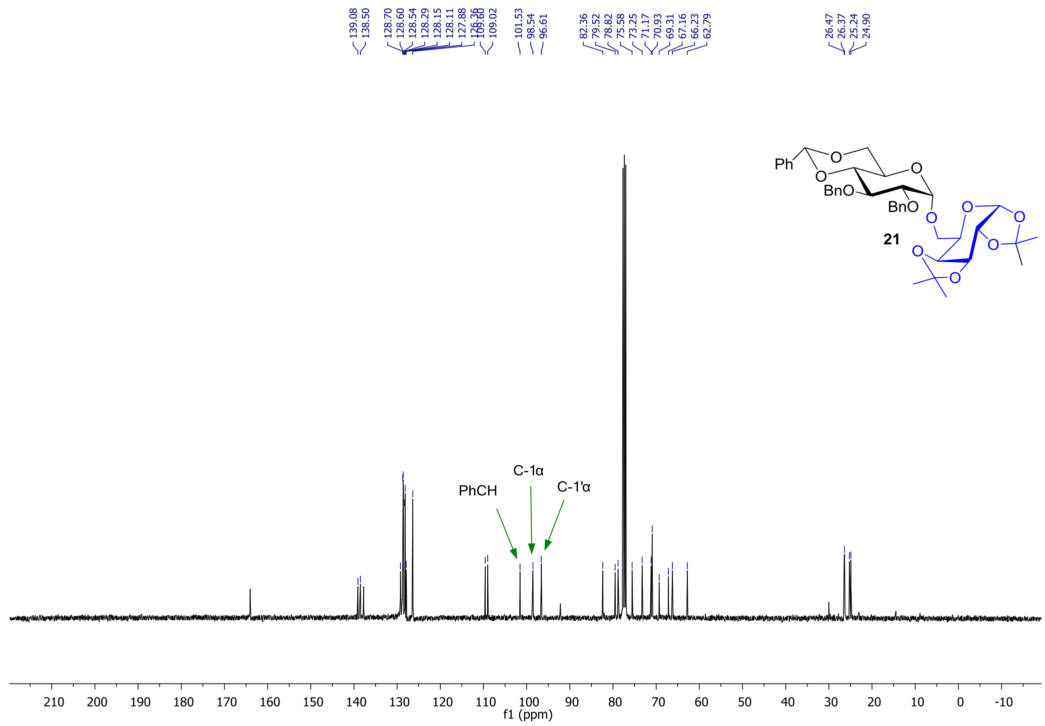

COSY spectrum of compound **21**

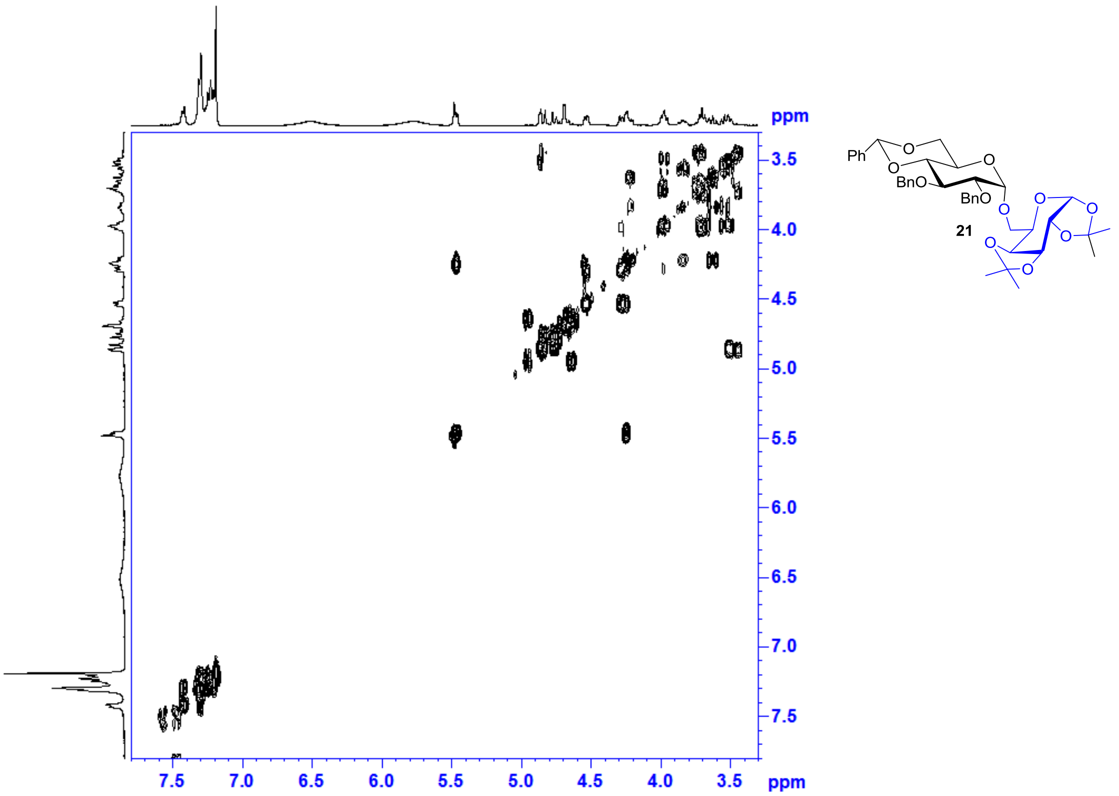

HSQC spectrum of compound **21**

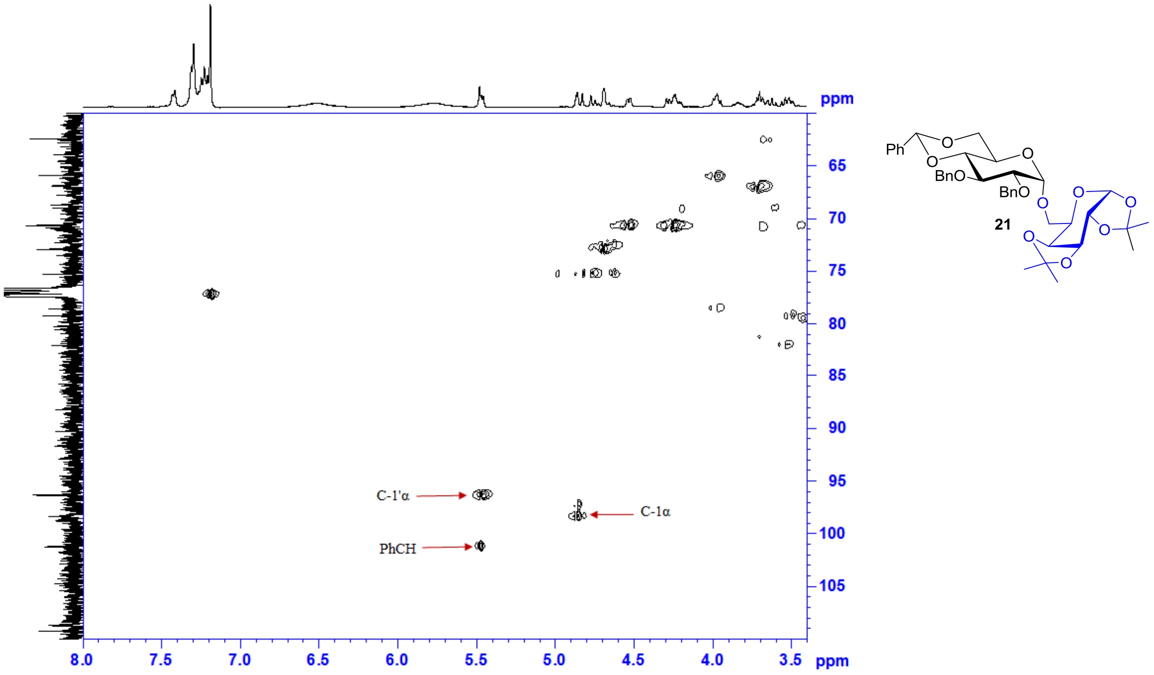

^{1}H NMR spectrum of compound **23** (400 MHz, $CDCl_3$)

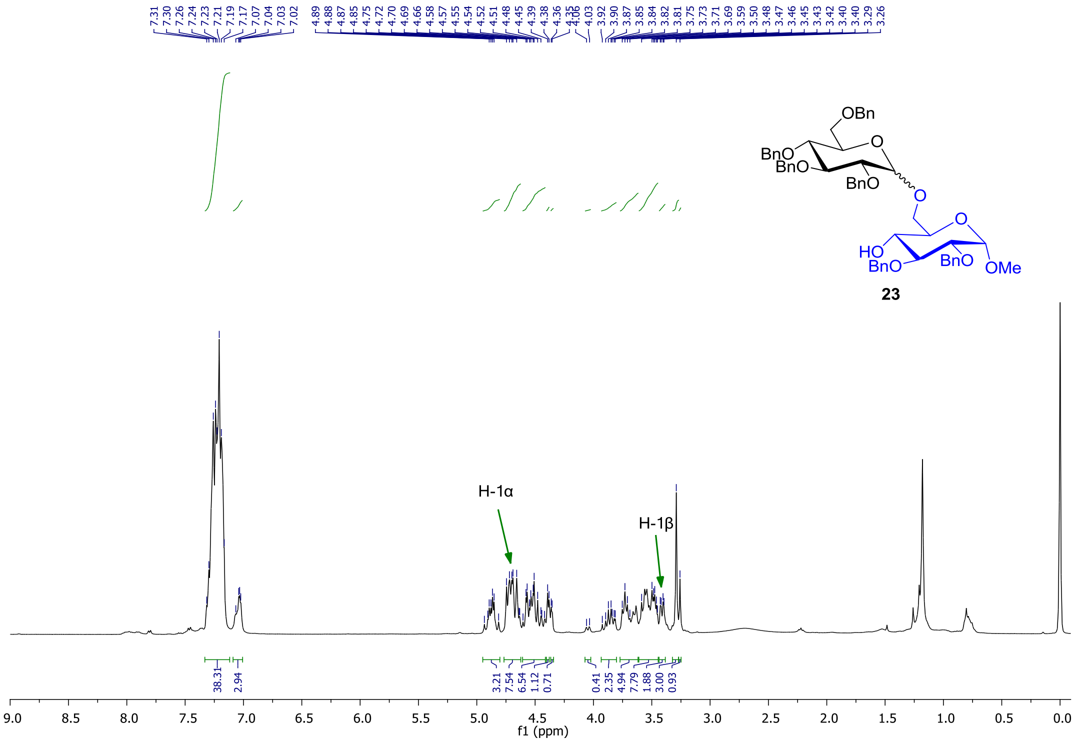

^{13}C NMR spectrum of compound **23** (100 MHz, $CDCl_3$)

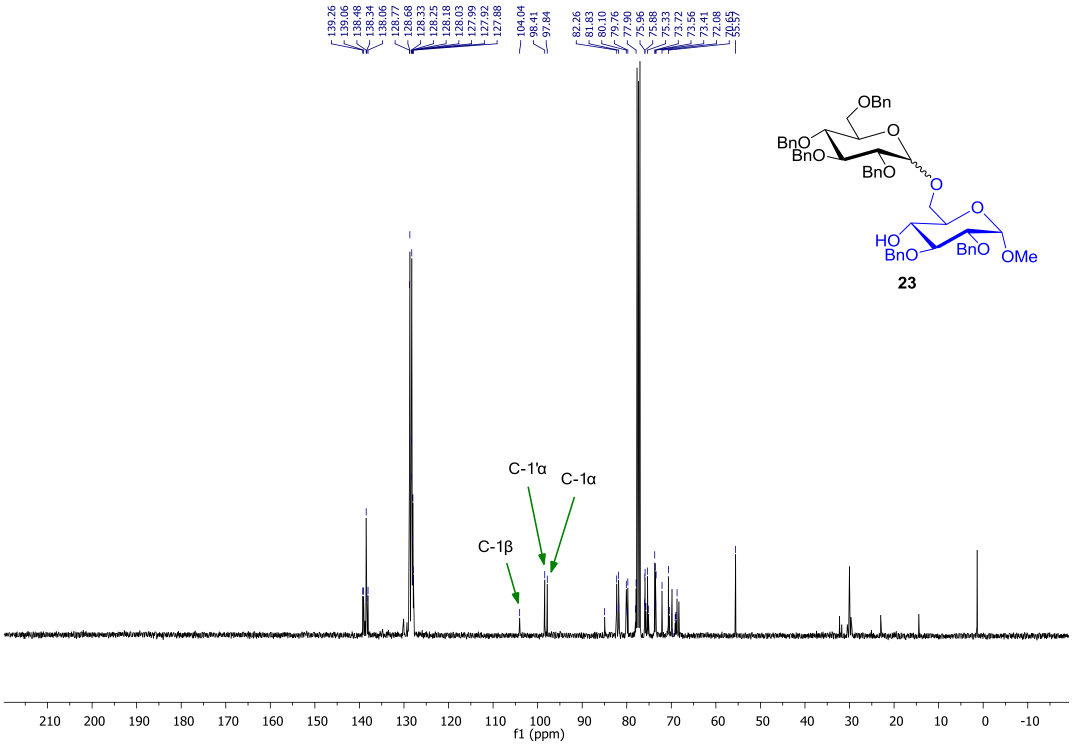

COSY spectrum of compound **23**

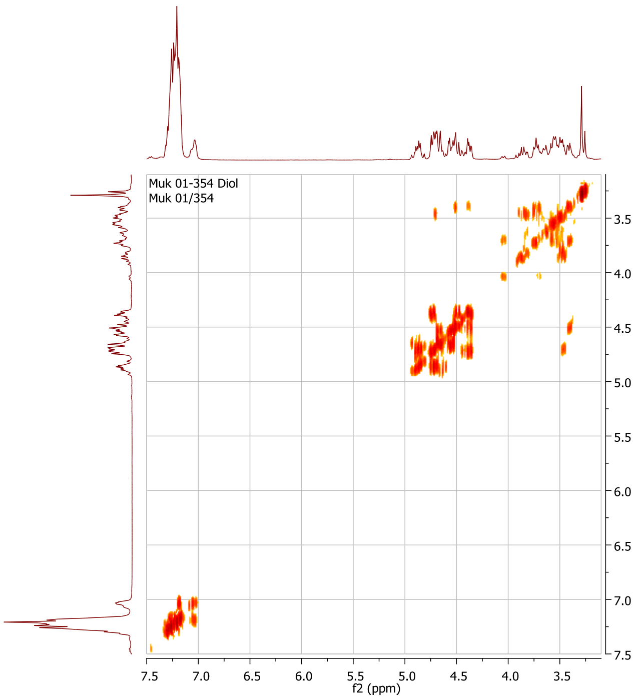

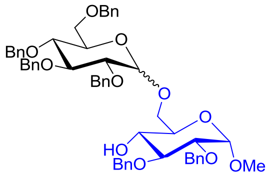

23

HSQC spectrum of compound **23**

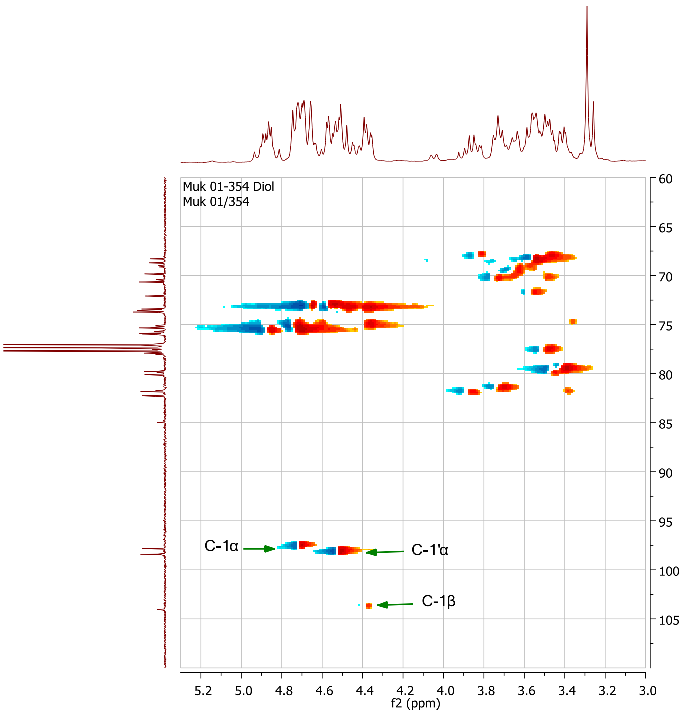

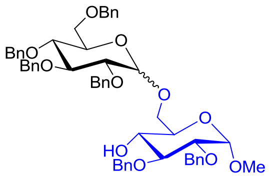

23

^{1}H NMR spectrum of compound **24** (400 MHz, $CDCl_3$)

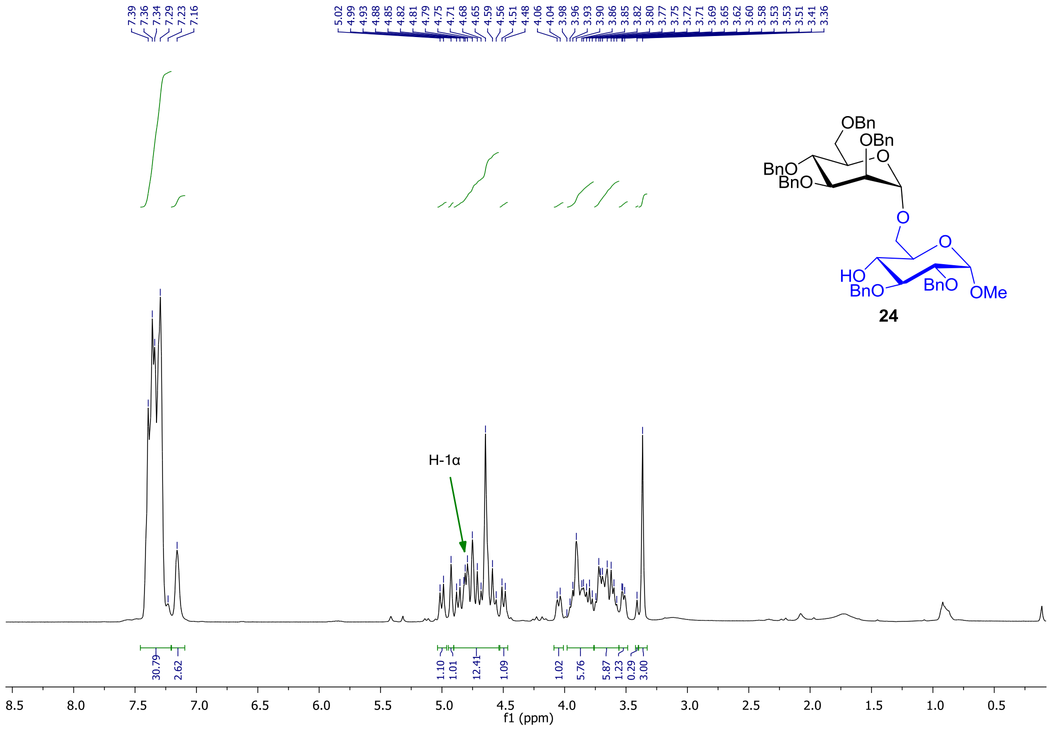

^{13}C NMR spectrum of compound **24** (100 MHz, $CDCl_3$)

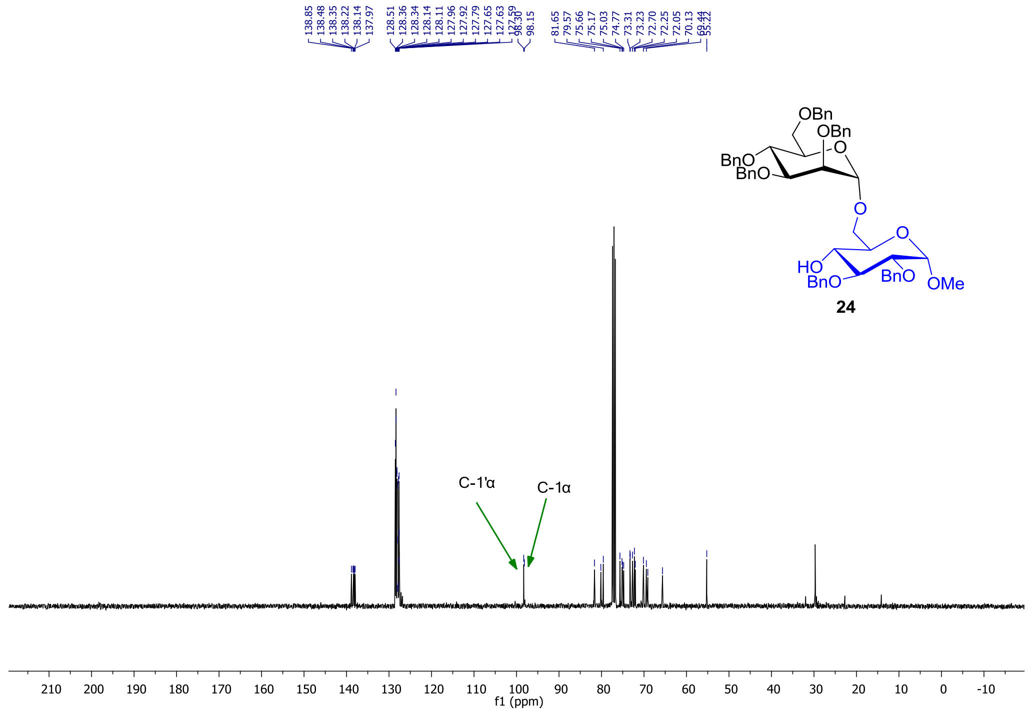

COSY spectrum of compound **24**

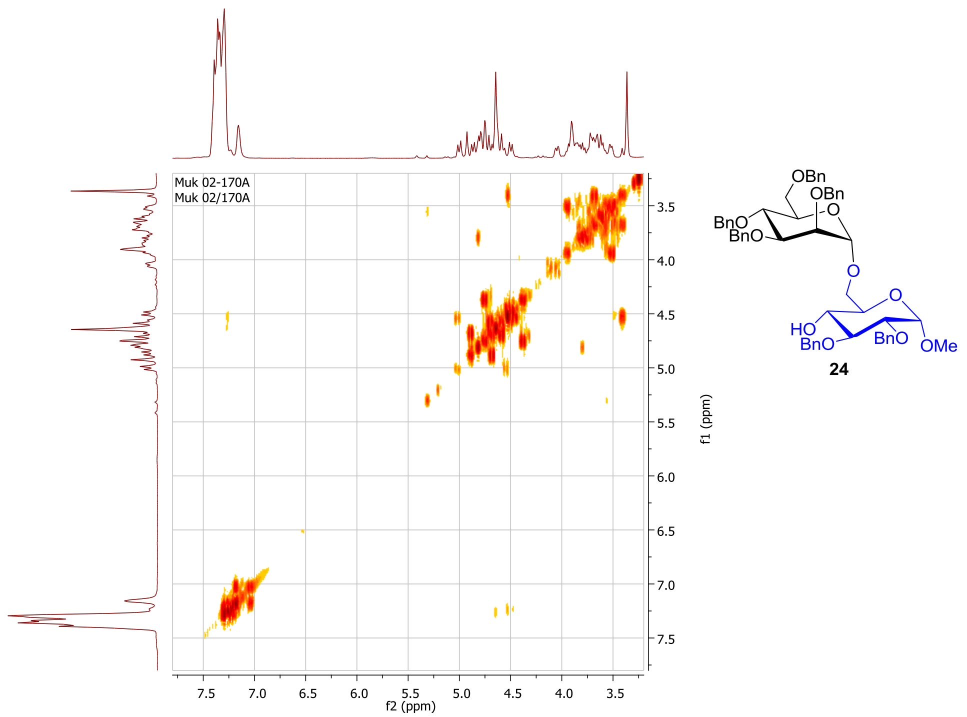

HSQC spectrum of compound **24**

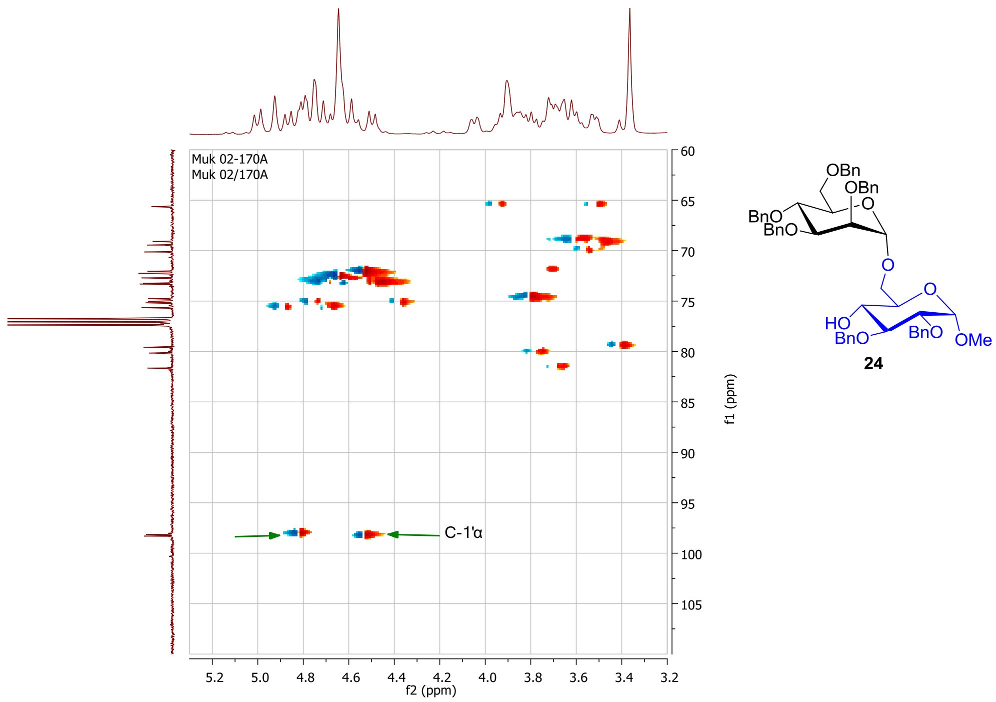

Supplement: File 1 — Experimental procedures and analytical data. [file Beilstein_J_Org_Chem-13-2385-s001.pdf]
